# Supplementary material for: Dual recognition drives site-directed G-quadruplex stabilization: Oligonucleotide design in G4 ligand-oligonucleotide conjugates
Source: Mol Ther Nucleic Acids. 2026 Aug 14;37(3):103061. doi: 10.1016/j.omtn.2026.103061 (PMC13551894; doi:10.1016/j.omtn.2026.103061)
Supplement: Supplementary file 1 [file mmc1.pdf]

OMTN, Volume *37*

Supplemental information

Dual recognition drives site-directed G-quadruplex stabilization: Oligonucleotide design in G4 ligand-oligonucleotide conjugates

Alva Abrahamsson, Sakina Khwaja, Namrata Chaudhari, Steven Vertueux, Andreas Berner, Koit Aasumets, Chandan Kumar, Lotte Stietz, Tom Baladi, Anders Dahlén, Sjoerd Wanrooij, and Erik Chorell

Oligonucleotide conjugations

DNA Oligonucleotide conjugations

All oligonucleotides received through a collaboration with the Oligonucleotides and Targeted Delivery group, Discovery Sciences at AstraZeneca R&D (Gothenburg, Sweden) or purchased from IDT (Integrated DNA Technologies, Sweden). 5´-hexylamine oligonucleotide (Na^+ salt, 50 nmol) was dissolved in 0.1 M $NaHCO_3$ (aq) (50 µL, 1 mM). A solution of (1R,8S,9s)-bicyclo[6.1.0]non-4-yn-9-ylmethyl N-succinimidyl carbonate (BCN-NHS ester) (20 equiv., 5 µL, 0.2 M) was freshly prepared and added to the oligonucleotide solution. The reaction solution was vortexed and shaken for 24 h. Followed by precipitation of oligonucleotide conjugate using 10% v/v of 3 M NaOAc and 4 times the volume EtOH and kept in freezer for 30 min. Then the solution was centrifuged for 15 min followed by removing the supernatant. The precipitate pellet was resuspended in the same volume of NaOAc and EtOH and repeated the precipitation. After this, the pellet was washed with only EtOH followed by drying the pellet by gently adding N_2 gas and then resuspended in 0.1 M $NaHCO_3$ (aq) (50 µL, 1 mM). The G4-ligand baring an azide functionality (previously described)[1] (2.5 equiv., 25 µL, 10 mM in DMSO), was added to the dissolved oligonucleotide and vortexed and shaken for 24 h. The reaction mixture was dissolved in more water and DMSO and filtered before purified by RP-HPLC. Purification was done using a C18 semi-preparative column and 2 mL/min flowrate and a linear gradient of 5 to 60% solvent B (100% ACN). Solvent A was a 50 mM triethylammonium acetate buffer. The conjugation products were confirmed with HRMS (ESI^-).

Peptide Nucleic Acid synthesis and conjugations

All reagents and solvents including resins, piperidine, hexafluorophosphate azabenzotriazole tetramethyl uronium (HATU), *N*,*N*-Diisopropylethylamine (DIPEA), trifluoro acetic acid (TFA), m-cresol, *N*-Methyl-2-pyrrolidone (NMP), acetonitrile (ACN), formic acid, diethyl ether was purchased from *Merck Sigma Aldrich*. The Fmoc-PNA(Bhoc)-OH monomers were purchased from *Iris Biotech GmbH*, Fmoc-6-Ahx-OH was purchased from *Chemtronica,* Peptide Grade dimethylformamide (DMF) was purchased from *CARLO ERBA Reagents*. The PP-reactors were purchased from *Biotage*. RP-HPLC was carried out on a Hitachi HPLC system using a Phenomenex Luna® Omega 5 µm Polar C18 100Å (250x 10 mm) column.

Peptide grade DMF was used for PNA synthesis. The reported equivalents are relative to the amount of resin, and all reactions were performed at room temperature.

The PNAs were synthesized on a 5-20 µmol scale on a Syro I automated peptide synthesizer (Biotage) using preloaded resin Fmoc-Lys(Boc)-Wang resin (loading 0.61 mmol/g) for **P6-P10** and Fmoc-Lys(Boc)-Wang LL (loading 0.2 – 0.4 mmol/g) for **P12-P15**. The PNA synthesis was performed in PP reactors with PE frit, and deprotection and cleavage were performed in PP reactors with PTFE frit.

The resin was allowed to swell in DCM for 30 min under agitation. Fmoc deprotection cycle was performed with 20% (v/v) piperidine in DMF for 15 min. The Fmoc-PNA(Bhoc)-OH monomers (0.5 M in DMF, 5 eq) were coupled for 1 h using HATU (0.5M in NMP, 5 eq), DIPEA (2.0 M in DMF) for 60 minutes. A capping step was performed after each coupling using Ac_2O 50% (v/v) in DCM. After PNA elongation, the N-terminal was coupled with Fmoc-6-Ahx-OH, followed by Fmoc deprotection according to the aforementioned procedures. Conjugation to the G4-ligand was performed on a 5 µmol scale. The N-terminal amine of the monomers was coupled with G4-ligand (0.25 M in NMP, 2.5 eq), HATU (0.25 M in NMP, 5 eq), DIPEA (1.0 M in DMF), and the coupling time was extended to 24 h (Figure S16).

After the synthesis was completed, the resin was suspended in a mixture of TFA:m-cresol (4:1) and vortexed for 1.5 h, resulting in simultaneous deprotection and cleavage from the resin. The solution was filtered into ice-cold Et_2O and centrifuged. The white precipitate was purified using RP-HPLC. Purification was carried out at room temperature using detection at 260 nm on a C18 semi-preparative column with a 2.5 mL/min flow rate in a gradient system of 0-20% B over 24 min then 20-100% B over 2 min (B 100% ACN and (A 0.1 Formic acid in Milli-Q water) for **P6-P10** and gradient system of 0-30%

over 11 min B then 30-100% B over 15 min (B 0.1% TFA in ACN and A 0.1% TFA in Milli-Q water). The conjugated products were confirmed with HRMS (ESI$^+$).

In vitro assays

Microscale thermophoresis (MST). The G4 template consisting of the G4-forming sequence and the flanking sequence was purchased with a Cy5 5´-label. The G4 DNA was annealed in MST buffer (10 mM potassium phosphate, 100 mM KCl, 0.05% Tween 20, pH 7.4) by heating at 96 °C for 5 min followed by cooling down to room temperature before storing in the fridge overnight. All MST experiments were done on a Monolith NT.115 (Nanotemper, Germany) instrument and performed in MST buffer with standard Monolith capillaries. The G4 DNA concentration was kept constant at 20 nM, and the GL-Os were serial diluted (1:1) with highest concentration of 5-40 µM. For PNA-based GL-Os, binding experiments were conducted both in the presence and absence of DMSO, and no differences in thermophoresis traces or derived binding affinities were observed. MST traces and binding affinity constants (K_D) were obtained using the Monolith analysis software and plotted and visualized in GraphPad Prism 10.

Proton nuclear magnetic resonance spectroscopy (^{1}H NMR). G4 DNA was prepared by annealing in 10 mM potassium phosphate buffer (3 mM KCl, 110 µM G4 DNA, pH 7.4). The solution was heated to 96 °C for 5 minutes, then gradually cooled to room temperature and stored overnight at 4 °C. Prior to NMR analysis, 10% D_2O was added to the G4 DNA solution to achieve a final concentration of 100 µM, and the sample was transferred to a 3 mm NMR tube. GL-O (1 mM) was added to the DNA solution in two steps: first, 2 µL (0.5 equivalents) was added, and after 10 minutes, a ^{1}H NMR spectrum was recorded. A second aliquot (0.5 equivalents) was then added to achieve a 1:1 molar ratio with G4 DNA, and another spectrum was recorded. For DNA-based GL-Os, samples were prepared in aqueous buffer, whereas PNA-based constructs required the addition of DMSO to a final concentration of 4.5% (v/v) to ensure complete solubility. NMR experiments were conducted on an 850 MHz Bruker AVANCE III HD spectrometer equipped with a 5 mm TCI cryoprobe at 298 K. The transmitter frequency offset (O1P) was set at 4.7 ppm, with a spectral width of 22 ppm. One-dimensional ^{1}H NMR spectra were acquired using excitation sculpting with 512 scans per spectrum. For thermal stability studies, samples were equilibrated at temperatures ranging from 313 K to 338 K in 2.5–10 K intervals. Spectra were recorded after a 5-minute equilibration at each temperature. Data were processed using MestreNova version 10.0.2.
NMR experiments of PNA GL-Os **P6**-**P15** were done in 4.5% DMSO by adding DMSO-d_6 (20 µL) and D_2O (10 µL) to a G4 DNA solution (170 µL), to achieve a final concentration of 100 µM G4 DNA.

Taq polymerase STOP assay. Taq Polymerase stop assay was adapted from Berner et al.[2] DNA templates were annealed to fluorescent labelled primers in 100 mM KCl by heating to 95 °C for 5 minutes followed by slow cooling to room temperature. The indicated compound concentrations were added to 40 nM annealed template in 1x Taq Buffer (10 mM Tris-HCl pH 8.8, 50 mM KCl, Thermo Fisher Scientific), 1.5 mM $MgCl_2$, and 0.05 U/µL Taq Polymerase (Thermo Fisher Scientific). For PNA-based GL-Os, assays were performed both in the presence and absence of DMSO, and no differences in polymerase stalling or full-length product formation were observed. Samples were preincubated on ice (10 minutes) and reactions initiated with the addition of dNTPS (100 µM) and transferring the samples to 37 °C. After 15 min at 37 °C, reactions were stopped by addition of equal volume of 2x stop solution (0.5% SDS, 25 mM EDTA, XC-Dye in Formamide) and separated on a 12% polyacrylamide Tris-Borate-EDTA (TBE) gel containing 25% formamide and 8 M urea. Fluorescent signal was detected with a Typhoon Scanner (Amersham Biosciences). The intensity of the full-length band was quantified using Image Quant TL 10.2 software (GE Healthcare Life Sciences) and compared to sample without compound.
The PNA GL-Os **P6**-**P15** were run in a final concentration of 0.45% DMSO in the assay.

***In vitro* endonuclease digestion.** Stability of oligos against Mung bean nuclease digestion was assessed. 4 µM oligos were incubated with 3 units of enzyme (MBN, NEB) in 1X MBN buffer (MBN) at

30 degrees. At indicated time points, reactions were quenched by adding DNA Gel loading dye (50 mM EDTA, 50 mM HEPES, 30% Glycerol, 0.001% Bromophenol Blue) and heating at 95 degrees. Digestion products were analysed by polyacrylamide gel electrophoresis (PAGE). DNA samples were resolved on 16% TBE gels for 1 h at 100 V in 1X TBE buffer. PNA samples were loaded on 4-20% SDS PAGE gels (Mini-PROTEAN TGX Gels, Bio-Rad) and run for 1 h at 120 V in 1X SDS running buffer (Tris Glycine). TBE gels were stained with Gel red (Biotium), while SDS PAGE gels were stained with Instant Blue (Abcam). Gels were imaged using a ChemiDoc Imaging system (Bio-Rad).

CD melting temperature assay. G4 DNA (3 µM) was folded in K-phosphate buffer (10 mM, pH 7.4) containing KCl (3 mM) by heating for 5 minutes at 95°C, then allowed to cool to room temperature. PNA in DMSO was added in a 1:1 ratio, resulting in a final DMSO concentration of 0.3%. CD measurements were performed on a J-1700 Circular Dichroism Spectrophotometer (Jasco International Co. Ltd.). A quartz cuvette with a path length of 1 mm was used for measurements. CD spectra were recorded between λ = 200 – 350 nm with an interval of 0.5 nm and a scan rate of 100 nm/min. Thermal melting was recorded between 25°C and 95°C with increments of 10°C at a speed of 1 °C/min.

Cell line generation. To enable cellular uptake and tracking of GL-O internalization, a trivalent GalNAc moiety and a Cy3 fluorophore were incorporated at the 3′-end of an oligonucleotide, which was subsequently conjugated to the G4-ligand as described above. Flp-In™ T-REx™ 293 cells (Thermo Fisher Scientific) were engineered for doxycycline-inducible expression of the asialoglycoprotein receptor 1 (ASGR1). The ASGR1 coding sequence (CDS), flanked by restriction sites, was synthesized by Eurofins and subcloned into the pcDNA5/FRT/TO vector. The Flp-In™ system enables site-specific integration of the gene of interest via Flp recombinase–mediated recombination; its key features are summarized in Figure S2. Briefly, Flp-In™ T-REx™ 293 cells were co-transfected with pcDNA5/FRT/TO-ASGR1 (600 ng) and pOG44 (6 µg) using TurboFect (Thermo Fisher Scientific). Hygromycin-resistant isogenic colonies were pooled and expanded. ASGR1 expression following doxycycline induction (25 ng/mL) was confirmed by Western blot using an anti-ASGR1 antibody (Proteintech; Figure S2).

Microscopy. Uptake and intracellular localization of the GalNAc- and Cy3-modified GL-O were assessed by live-cell confocal microscopy. ASGR1-expressing HEK293 cells were seeded onto glass-bottom dishes and induced overnight with doxycycline (25 ng/mL). Cells were then incubated with 0.5 µM Cy3-conjugated GalNAc oligonucleotide for 24 h prior to imaging. Live-cell imaging was performed at the Biochemical Imaging Centre Umeå on a Leica SP8 FALCON confocal microscope equipped with a 63× oil-immersion objective.

Computational assessment

Sequence alignment analysis. The specificity of 5′ G4 flanking sequences of varying lengths was assessed by aligning these DNA sequences to the human reference genome. The human reference genome (GRCh38) was retrieved from the UCSC Genome Browser,[3] and flanking sequence was obtained from NCBI nucleotide database. FASTA datasets containing flanking sequences of 5–20 bases were generated and each dataset was independently aligned to the human reference genome using Bowtie 2.0,[4] with no mismatch allowed. The aligned reads in SAM files were converted to BAM format and subsequently sorted and indexed using SAMtools[5] with default settings. The final BAM files were used for downstream sequence annotation and comparative analysis of mapping specificity across different flanking lengths.

Table S1. Protocol for PNA synthesis

Step	Reagent/solvent	Volume (µL)	Cycles x Time
Swelling	DCM	500	30 minutes
Wash	DMF	5 x 500	5 x 1 minute
Deprotection	20% (v/v) piperidine in DMF	500	2 x 3 minutes<br>1 x 9 minutes
Wash	DMF	5 x 500	5 x 1 minute
Coupling	Monomer in DMF<br>HATU in NMP<br>DIPEA in DMF	1 x 200<br>1 x 210<br>1 x 100	60 minutes
Wash	DMF	5 x 500	5 x 1 minute
Capping	Ac2O 50% (v/v) in DCM<br>DIPEA	1 x 200<br>1 x 100	40 minutes
Wash	DMF	5 x 500	5 x 1 minute
Conjugation of G4-Ligand	G4-Ligand in NMP<br>HATU in NMP<br>DIPEA in DMF	1 x 200<br>1 x 210<br>1 x 100	24 h

Table S2. G4 DNA templates

Used in	G4 DNA	Sequence (5´-3´)
MST assay	*c-MYC* Pu24T	[Cy5]-AGT CAC TGA ATT GGA CGT GA TGA GGG TGG TGA GGG TGG GGA AGG
NMR Assay	*c-MYC* Pu24T	AGT CAC TGA ATT GGA CGT GA TGA GGG TGG TGA GGG TGG GGA AGG
Taq Polymerase Stop Assay	c-MYC Pu24T	AGT CA CTG AAT TGG ACG TGA TGA GGG TGG TGA GGG TGG GGA AGG CAC GTG AGT TGA GTG GAG TTG GAA GTA GGC ATA CCC CTA T
Polymerase Stop assay	15-nt TET primer	[TET]-GCC TAC TTC CAA CTC
Polymerase Stop assay	25-nt TET primer	[TET]-ATA GGG GTA TGC CTA CTT CCA ACT C

G4 forming sequence (red), nucleotide gap (green), primer site (blue)

Table S3. GL-O conjugates synthesized for this study

GL-O name	Conjugation method	Oligo 5´modification	Sequence length	Sequence (5´-3´)
**GL-O D6**	SPAAC	Amine-C6	6-nt	*TmCAmCGT
**GL-O D8**	SPAAC	Amine-C6	8-nt	*TmCAmCGTmCmC
**GL-O D10**	SPAAC	Amine-C6	10-nt	*TmCAmCGTmCmCAA
**GL-O D12**	SPAAC	Amine-C6	12-nt	*TmCACGTmCmCAATT
**GL-O D14**	SPAAC	Amine-C6	14-nt	*TmCAmCGTmCmCAATTmCA
**GL-O D15**	SPAAC	Amine-C6	15-nt	*TmCAmCGTmCmCAATTmCAG
**GL-O D16**	SPAAC	Amine-C6	16-nt	*TmCAmCGTmCmCAATTmCAGT
**GL-O D18**	SPAAC	Amine-C6	18-nt	*TmCAmCGTmCmCAATTmCAGTGA
**GL-O D20**	SPAAC	Amine-C6	20-nt	*TmCAmCGTmCmCAATTmCAGTGAmCT
**GL-O P6**	Amide	Amine-C6	6-nt	TCACGT
**GL-O P8**	Amide	Amine-C6	8-nt	TCACGTCC
**GL-O P10**	Amide	Amine-C6	10-nt	TCACGTCCAA
**GL-O P12**	Amide	Amine-C6	12-nt	TCACGTCCAATT
**GL-O P15**	Amide	Amine-C6	15-nt	TCACGTCCAATTCAG
**GL-O TM1-3**	SPAAC	Amine-C6	15-nt	*mCTTmCGTmCmCAATTmCAG
**GL-O TM4-6**	SPAAC	Amine-C6	15-nt	*TmCATTmCmCmCAATTmCAG
**GL-O TM7-9**	SPAAC	Amine-C6	15-nt	*TmCAmCGTTTTATTmCAG
**GL-O TM10-12**	SPAAC	Amine-C6	15-nt	*TmCAmCGTmCmCATmCmCmCAG
**GL-O TM13-15**	SPAAC	Amine-C6	15-nt	*TmCAmCGTmCmCAATTTTT
**GL-O D15 PS-Cy3-GalNac**	SPAAC	Amine-C6	15-nt	*Tm*C*A*mC*G*T*mC*mC*A*A*T*T*mC*A*G-Cy3-3´-valent GalNAc

mC stands for methylated cytidine. * Phosphorothioate (PS) modification.

Table S4. Yields and mass of the GL-O conjugates

GL-O name	Yield (%)	Calc. MS (g/mol)	Found MS (m/z)
**GL-O D6**	66	2611,2446	2608,7248
**GL-O D8**	82	3217,6661	3215,8500
**GL-O D10**	57	3844,0856	3841,9774
**GL-O D12**	33	4452,4751	4452,0618
**GL-O D14**	43	5068,8957	5068,1141
**GL-O D15**	48	5398,1044	5397,2420
**GL-O D16**	30	5702,2992	5699,2611
**GL-O D18**	33	6344,7177	6344,3184
**GL-O D20**	54	6952,1232	6951,3930
**GL-O P6**	2	2289,0450	2290,0500
**GL-O P8**	6	2791,2788	2792,2542
**GL-O P10**	5	3341,4749	3342,4775
**GL-O P12**	5	3873.6779	3875,6694
**GL-O P15**	4	4691.0008	4692,9976
**GL-O TM1-3**	38	5389,0894	5373,1840
**GL-O TM4-6**	33	5373,0904	5390,1644
**GL-O TM7-9**	31	5391,0574	5388,2091
**GL-O TM10-12**	33	5361,1234	5386,1752
**GL-O TM13-15**	46	5365,0594	5364,1672
**GL-O D15 PS-Cy3-GalNAc**	55	7918.0527	7917.9102

Table S5. Three site complementarity of a 13-nt oligonucleotide binding sequence of *c-Myc* Pu27.

Chromosome	Matching site (gene)	Functions	Gene ID	Start-end position	Annotation	Sequence (flanking ±25)*
8	MYC proto-oncogene	-	ENSG00000136997	127735951 - 127735964	Promoter	CGCCCTCTGCTTTGGGAACCCGG GA**GGGGCGCTTATGG**GGAGGGT GGGGAGGGTGGGGAAGGT
1	LMX1A (LIM homeobox transcription factor 1 alpha)	Transcription factor regulates gene expression involved in the development of neurons and pancreas.	ENSG00000162761	165213724 - 165213737	Promoter	AAGGAACTGCTGAGGAAGGCAA GGA**CCATAAGCGCCCC**AAACGTC CGAGAACCATCTTGACAA (antisense) TTGTCAAGATGGTTCTCGGACGT TT**GGGGCGCTTATGG**TCCTTGCC TTCCTCAGCAGTTCCTT
12	ABTB3 (ankyrin repeat and BTB domain containing 3)	Specifically expressed in cortical and hippocampal inhibitory interneurons and support in synaptic functions.	ENSG00000151136	107523249 - 107523262	Intronic	AGTCAGTAGCTGATCCAAGCCAT AG**GGGGCGCTTATGG**CCACTAG AGAGCTACTTTCCTGGCA
2	CASC11 (LncRNA)	Cancer susceptibility candidate involved in gene regulation and found to be overexpressed in various cancers.	-	17657528-17657585	Intergenic	AAACGAAGTGAACTGTATCT**GGG GCGCTTATGG**AAAACAAACCCTC TAGCAGACTCAC

*Sequence highlighted in red and G4-forming sequence is underlined.

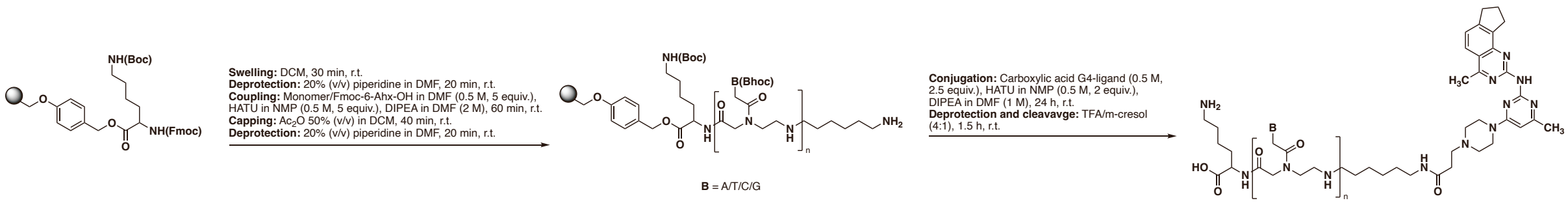

Figure S1. Scheme of PNA synthesis and G4-ligand conjugation.

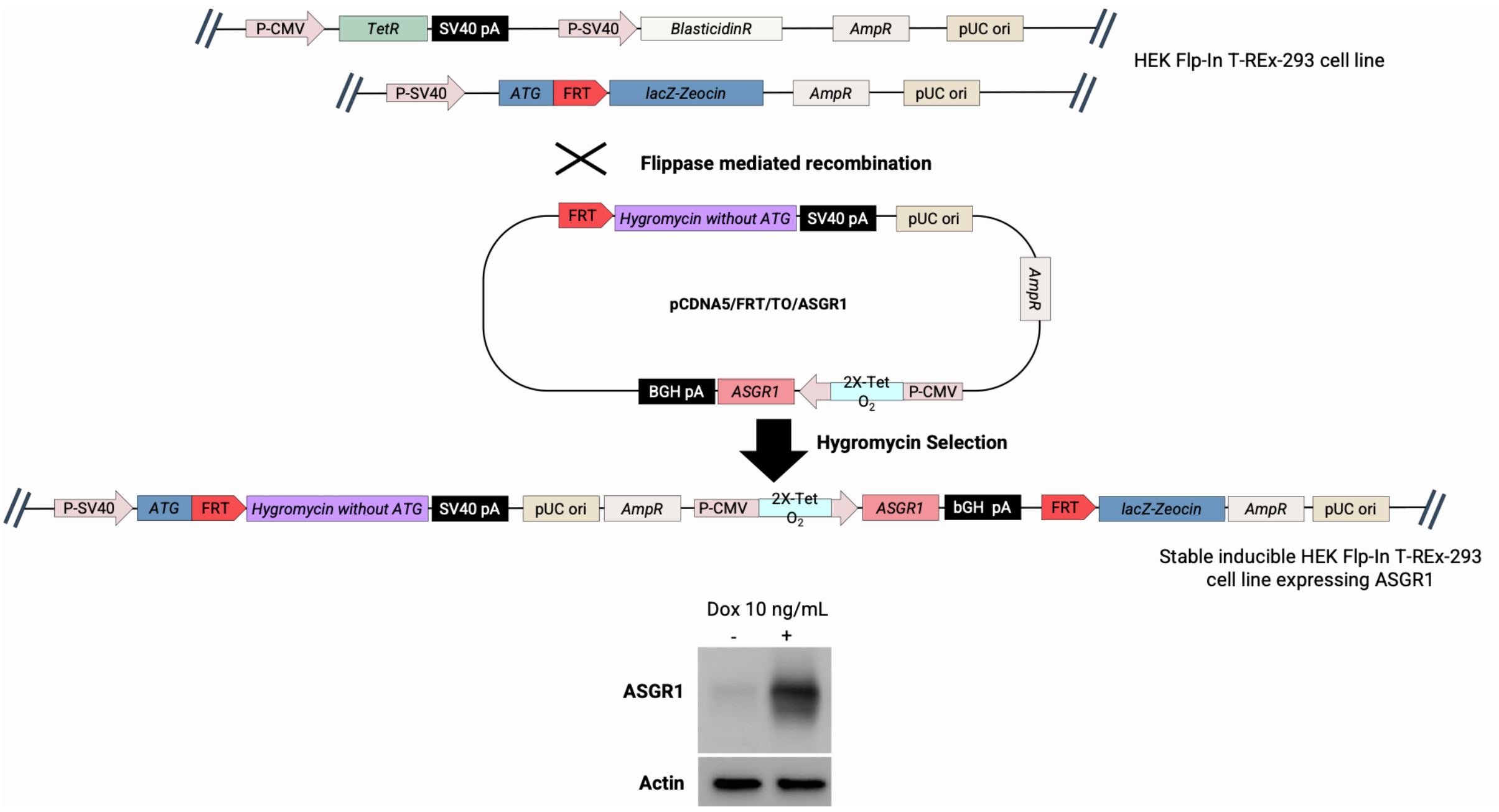

Figure S2. Schematic representation of the generation of a ASGR1-expressing cell line used to analyse GalNAc-mediated cellular uptake.

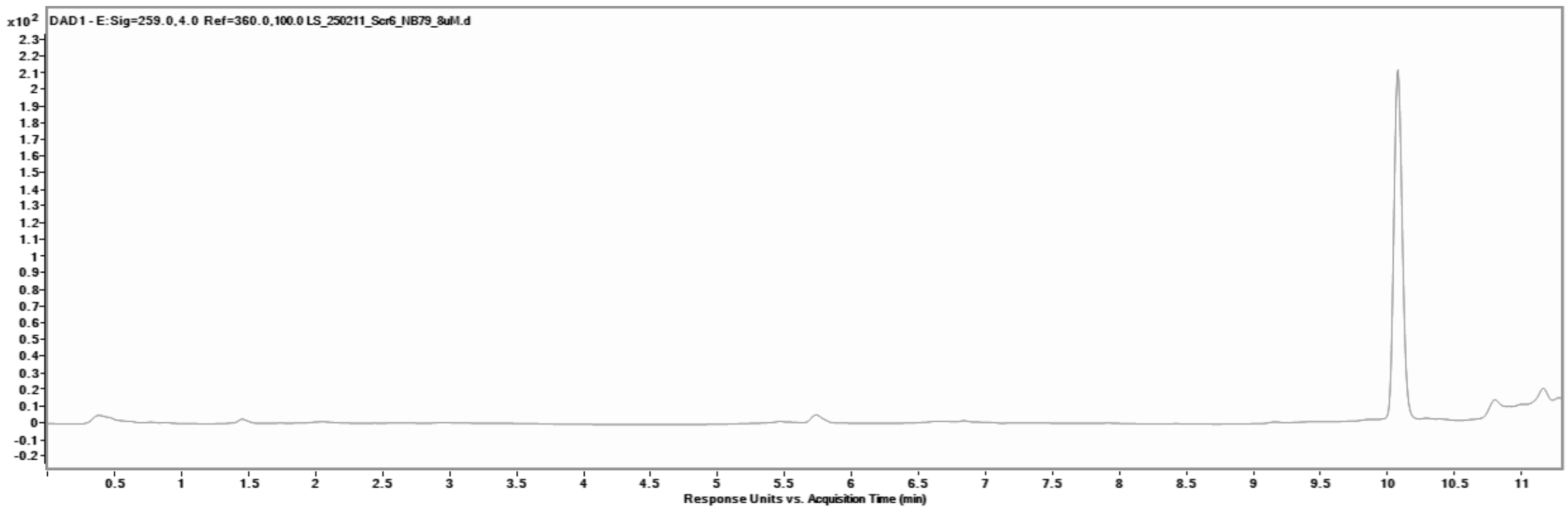

Figure S3. UV chromatogram at 259 nm of **GL-O D6**.

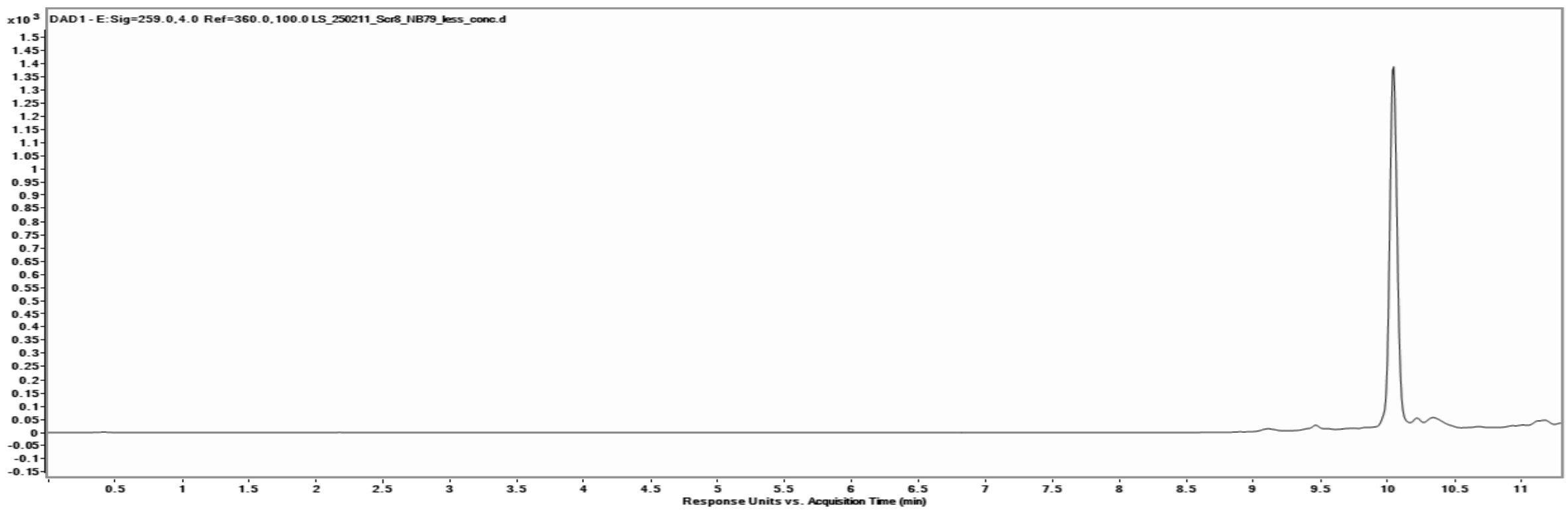

Figure S4. UV chromatogram at 259 nm of **GL-O D8**.

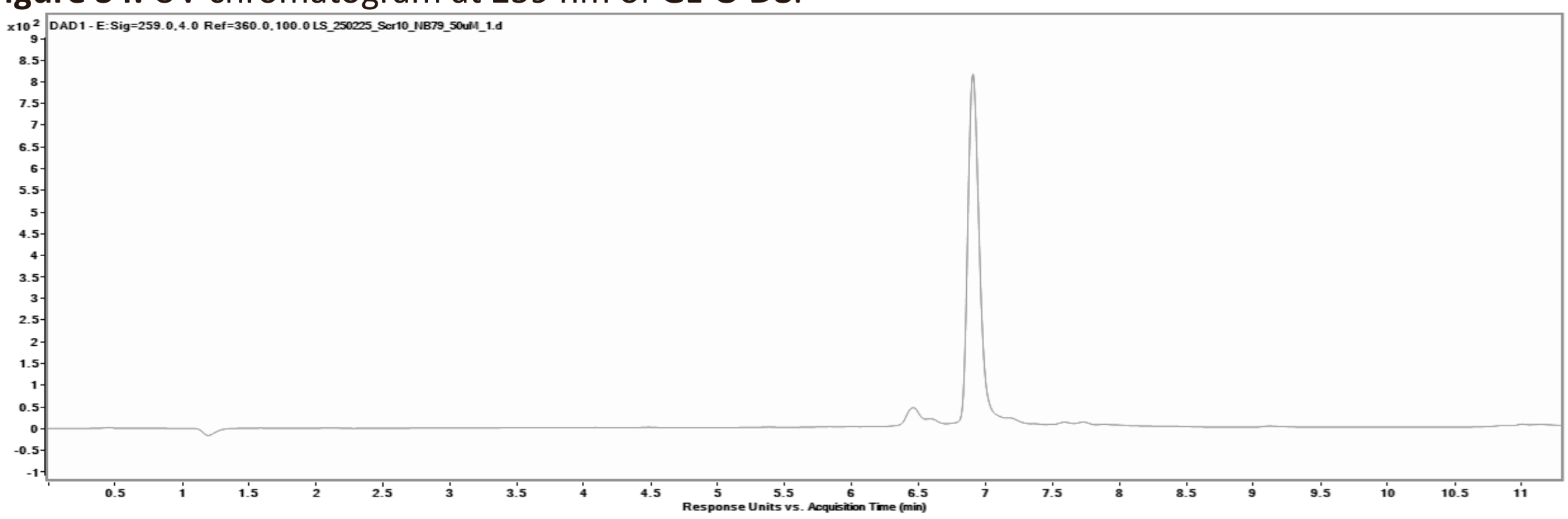

Figure S5. UV chromatogram at 259 nm of **GL-O D10**.

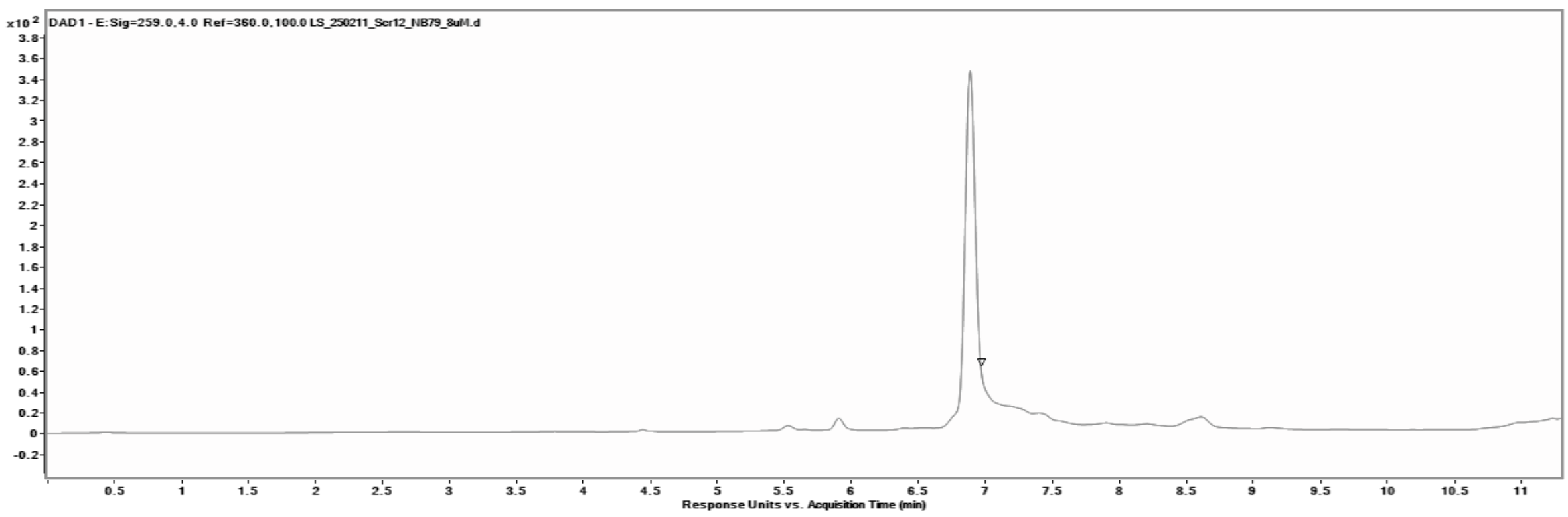

Figure S6. UV chromatogram at 259 nm of **GL-O D12**.

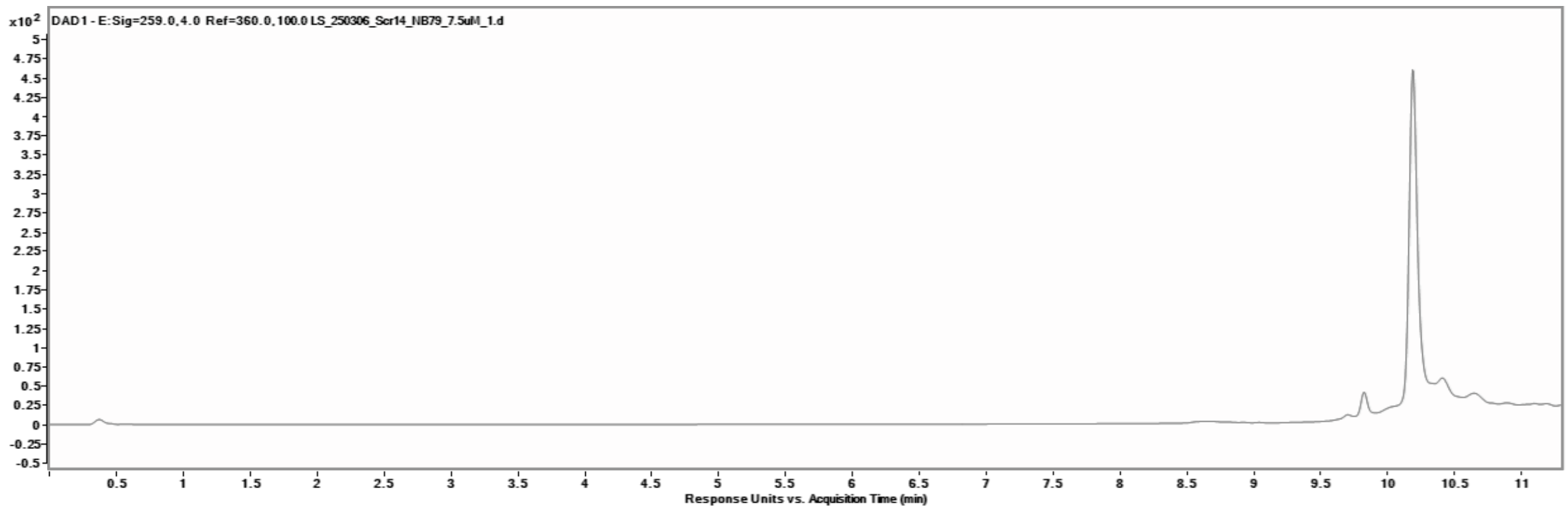

Figure S7. UV chromatogram at 259 nm of **GL-O D14**.

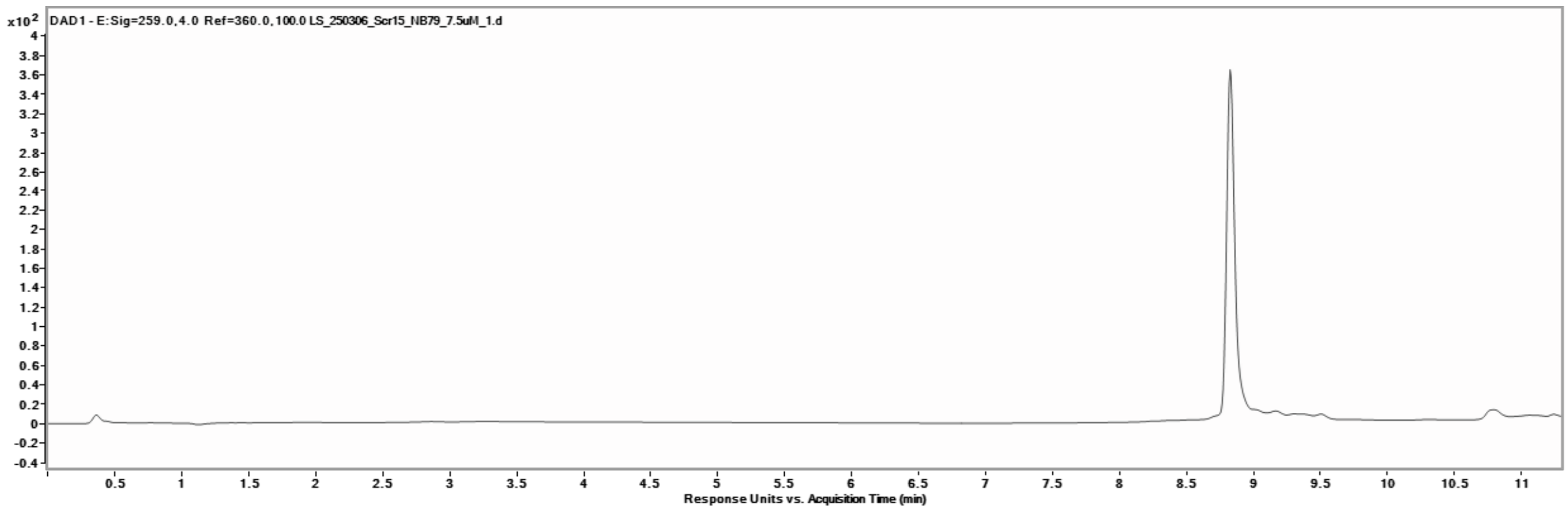

Figure S8. UV chromatogram at 259 nm of **GL-O D15**.

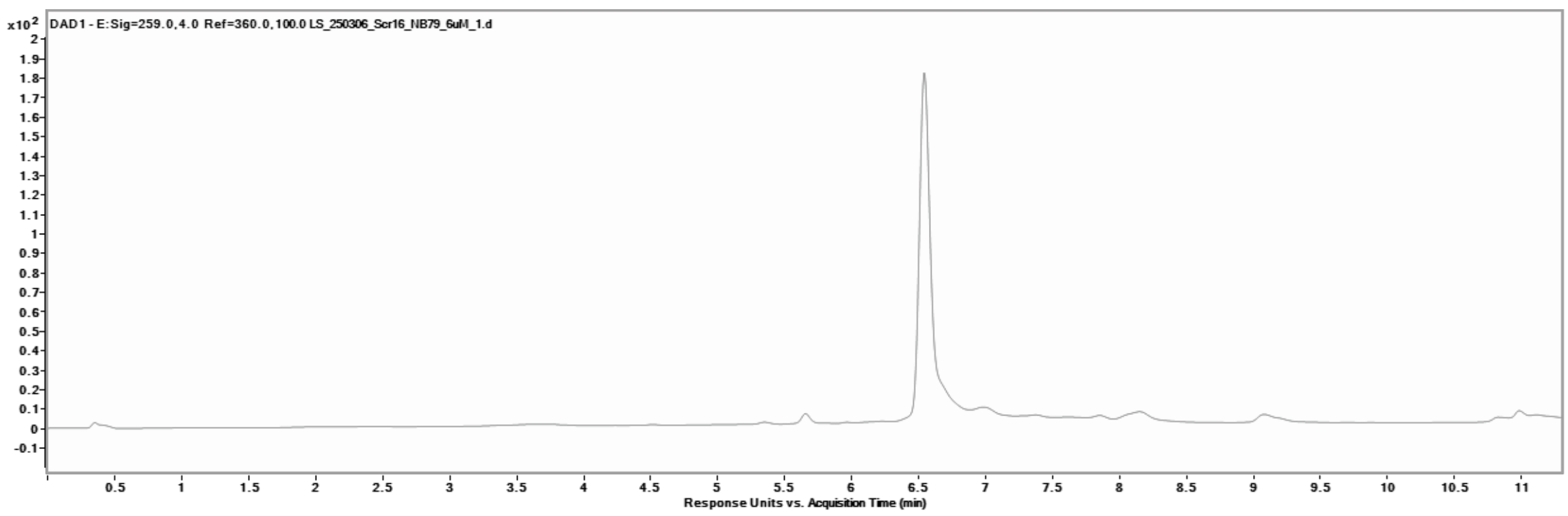

Figure S9. UV chromatogram at 259 nm of **GL-O D16**.

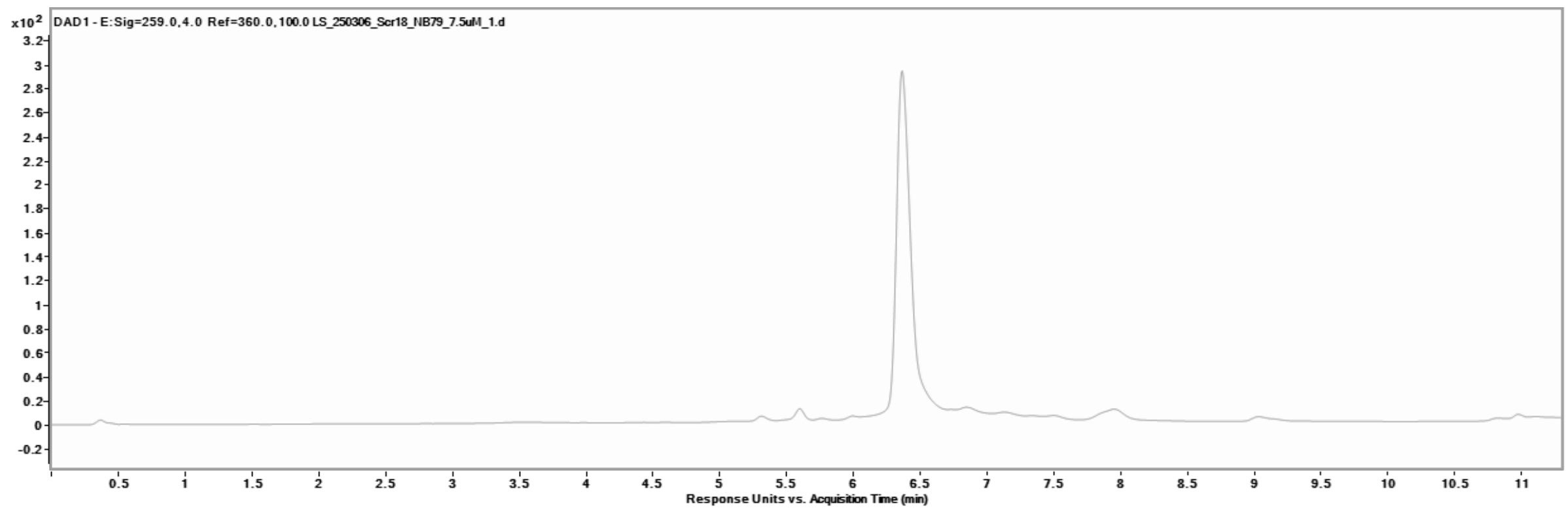

Figure S10. UV chromatogram at 259 nm of **GL-O D18**.

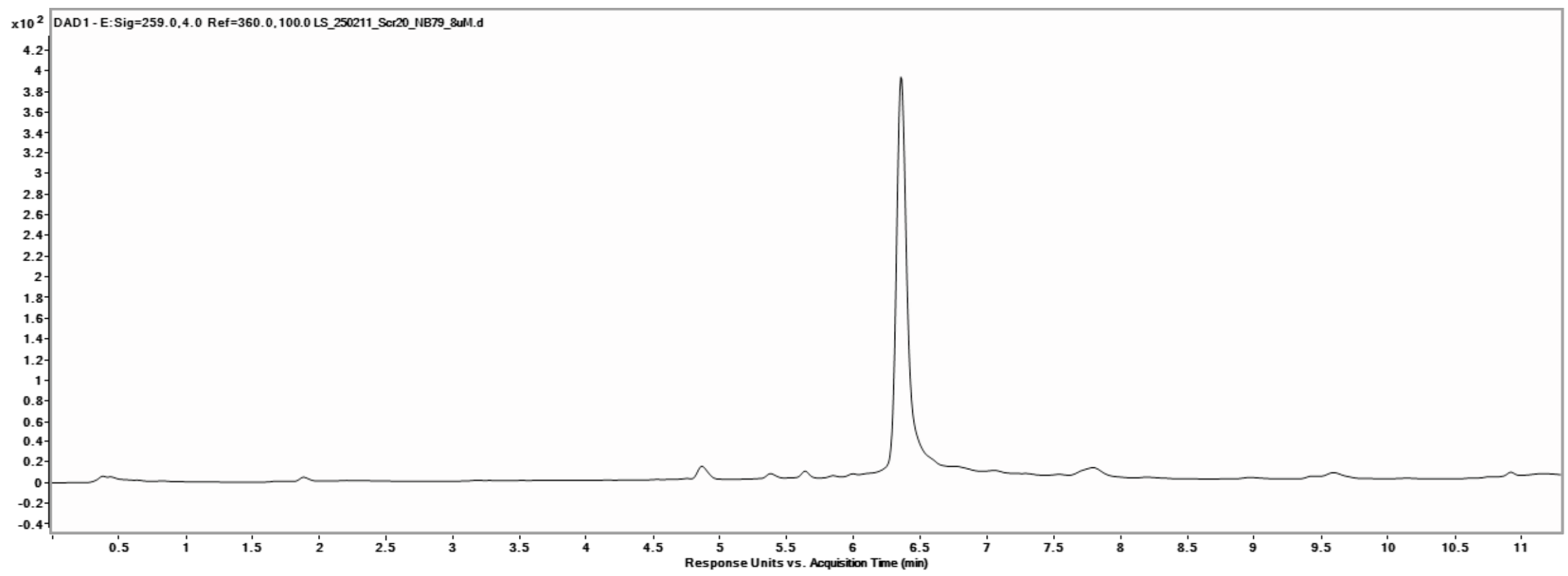

Figure S11. UV chromatogram at 259 nm of **GL-O D20**.

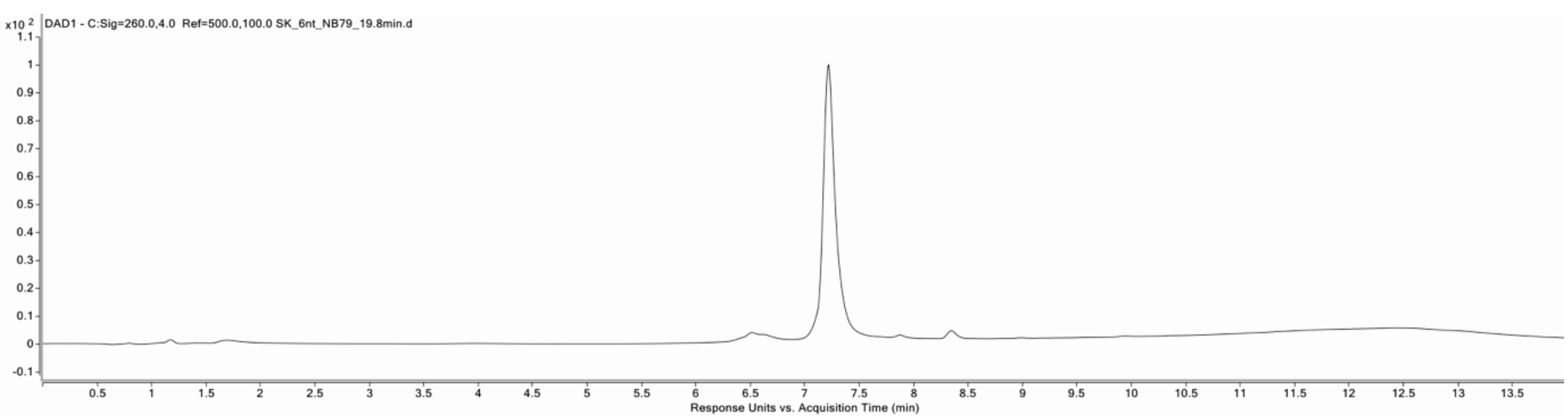

Figure S12. UV chromatogram at 259 nm of **GL-O P6**.

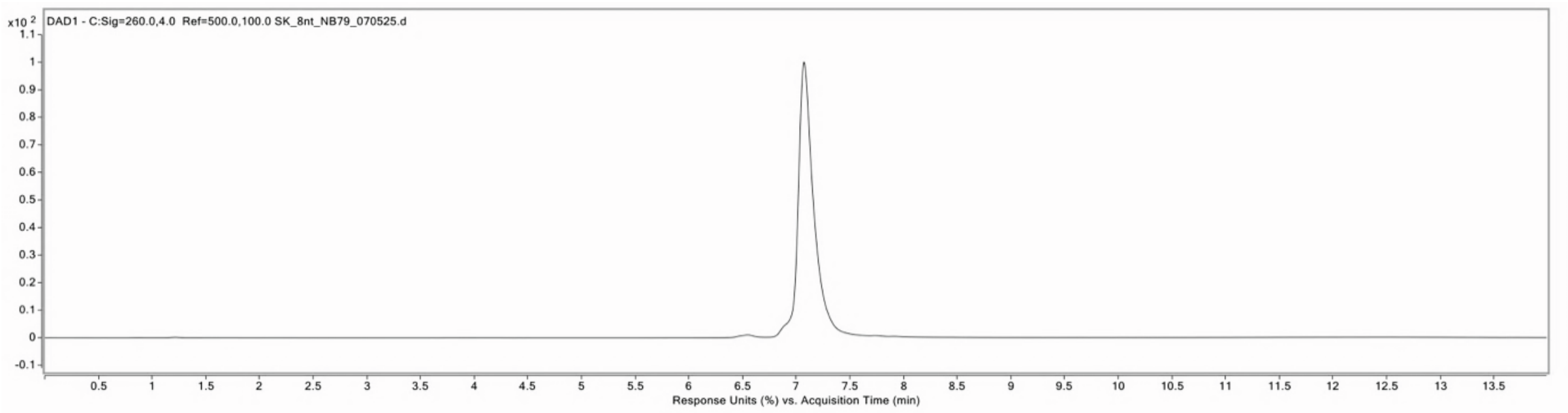

Figure S13. UV chromatogram at 259 nm of **GL-O P8**.

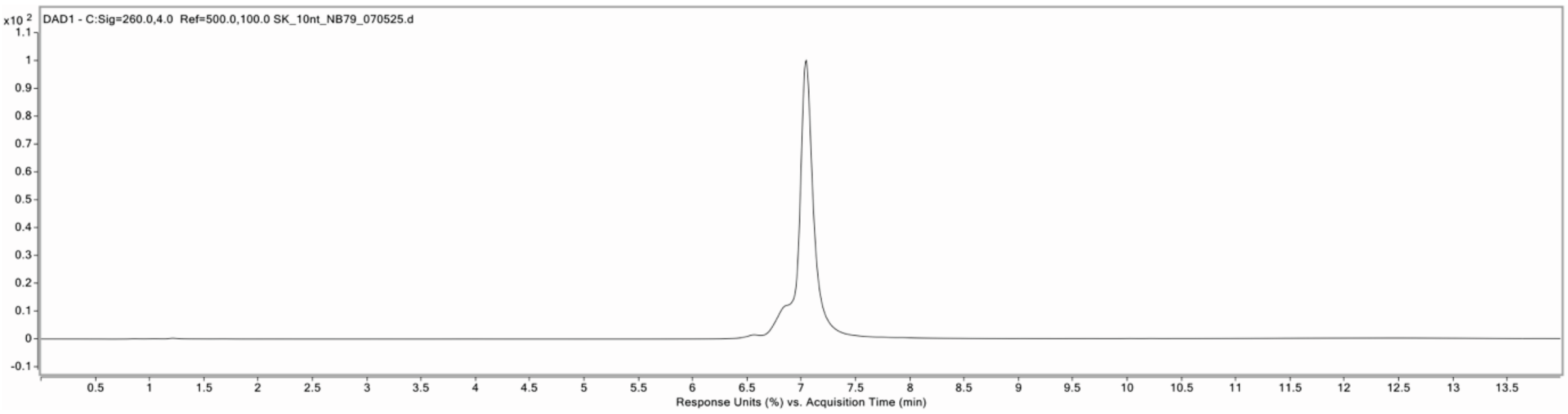

Figure S14. UV chromatogram at 259 nm of **GL-O P10**.

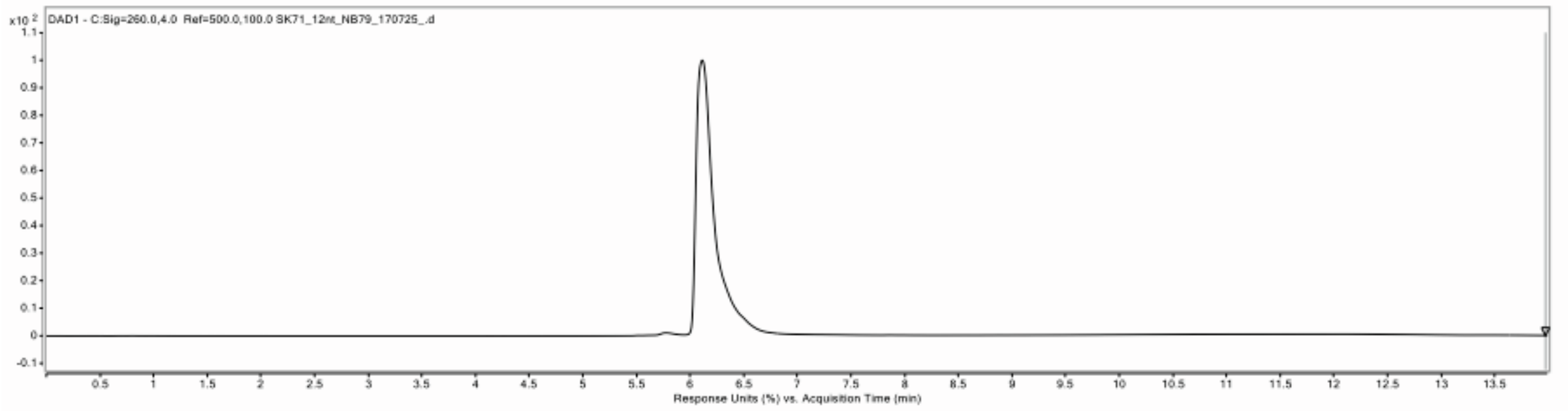

Figure S15. UV chromatogram at 259 nm of **GL-O P12**.

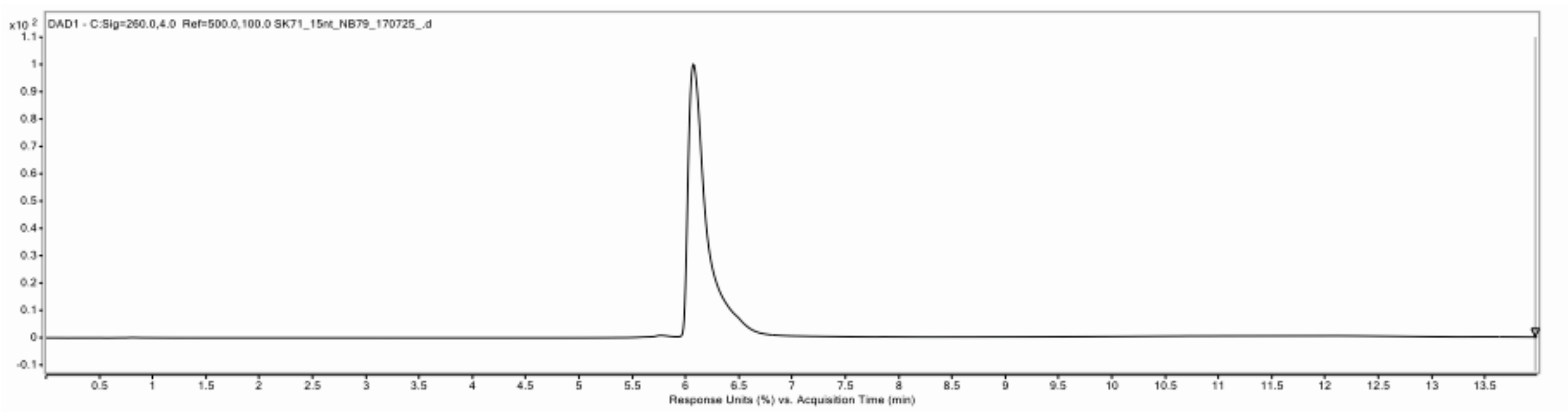

Figure S16. UV chromatogram at 259 nm of **GL-O P15**.

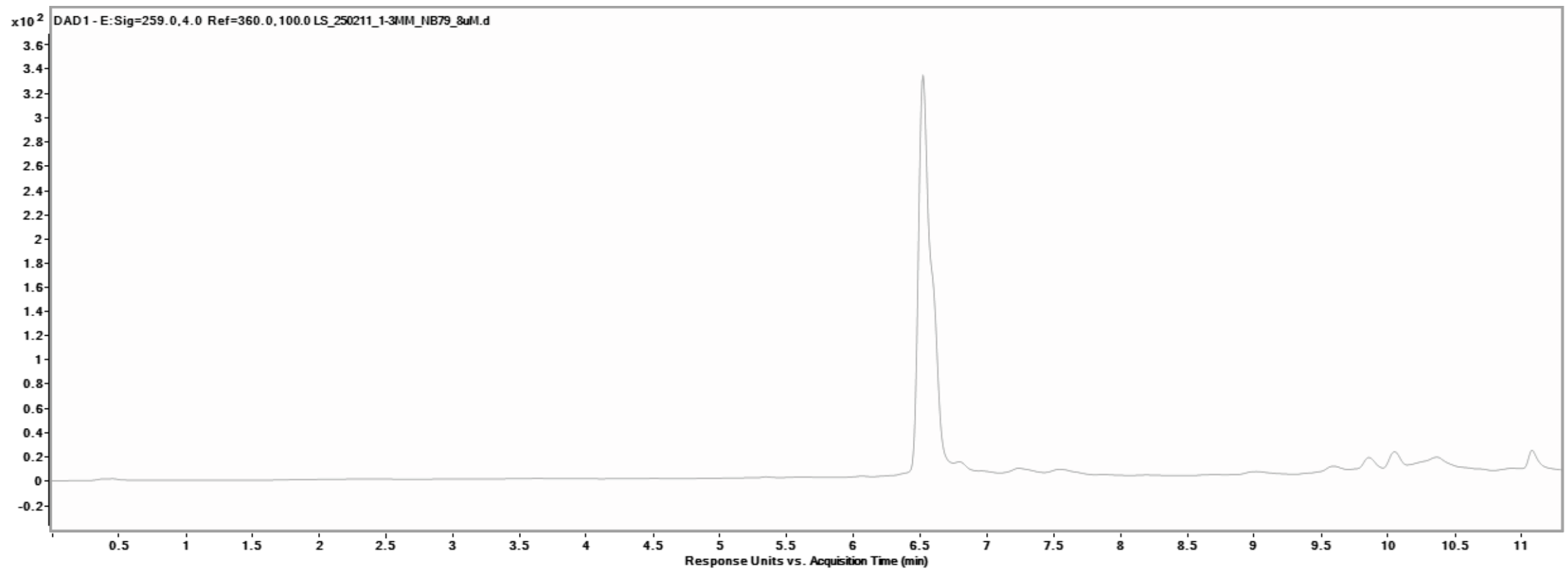

Figure S17. UV chromatogram at 259 nm of **GL-O TM1-3.**

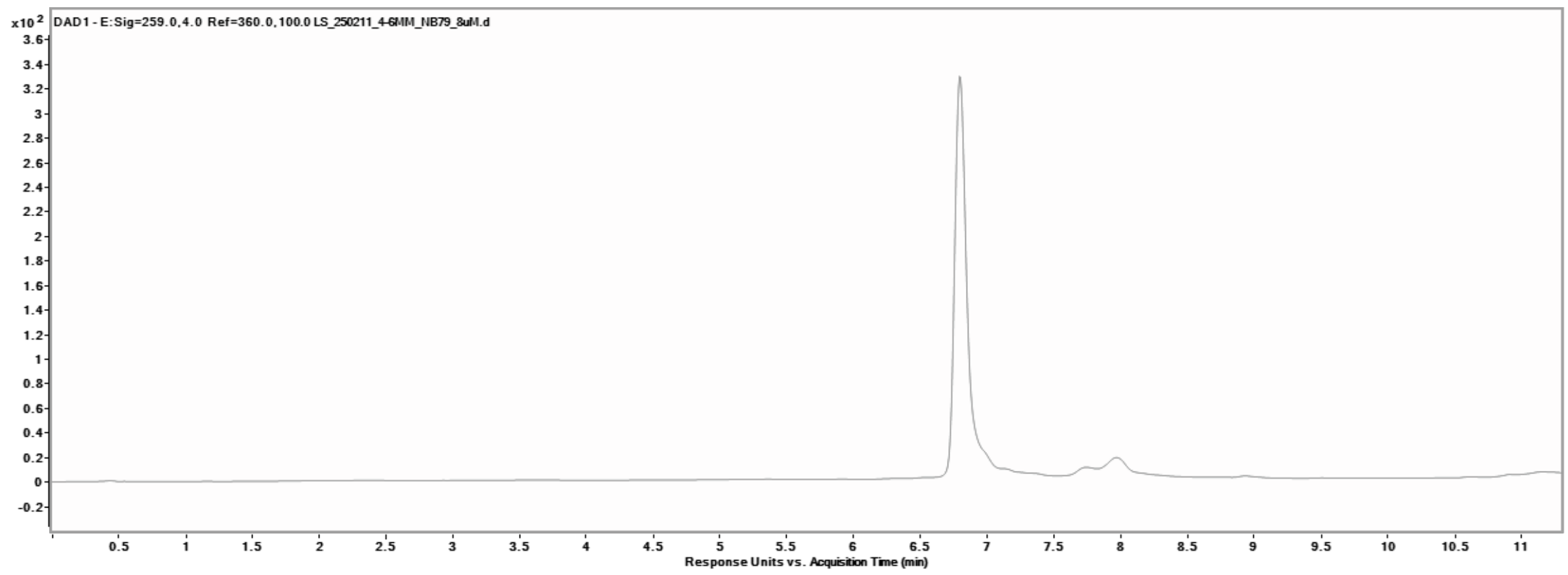

Figure S18. UV chromatogram at 259 nm of **GL-O TM4-6.**

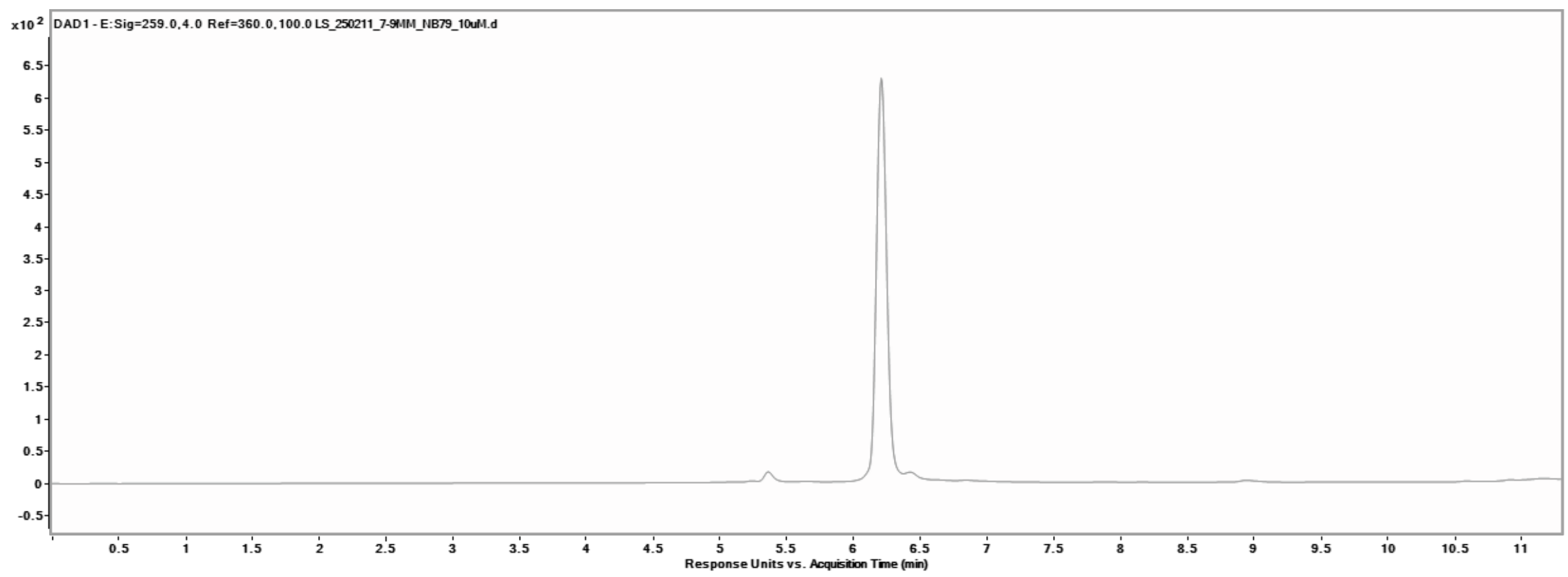

Figure S19. UV chromatogram at 259 nm of **GL-O TM7-9.**

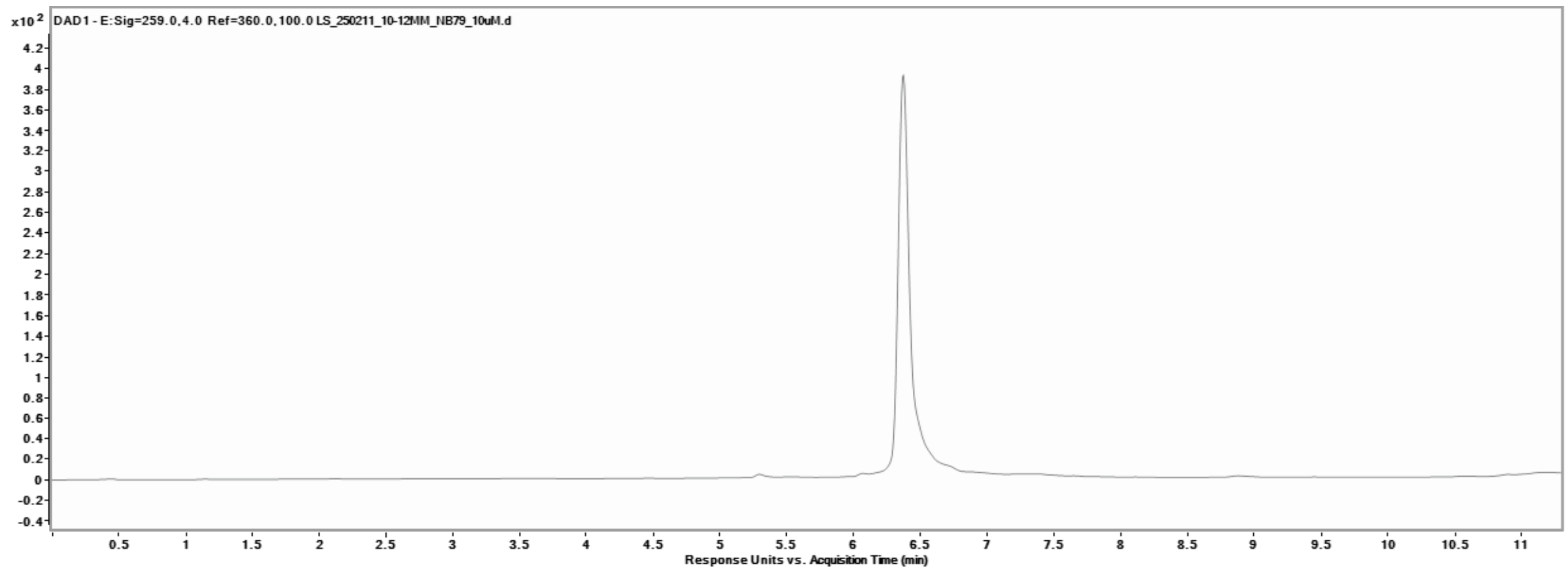

Figure S20. UV chromatogram at 259 nm of **GL-O TM10-12.**

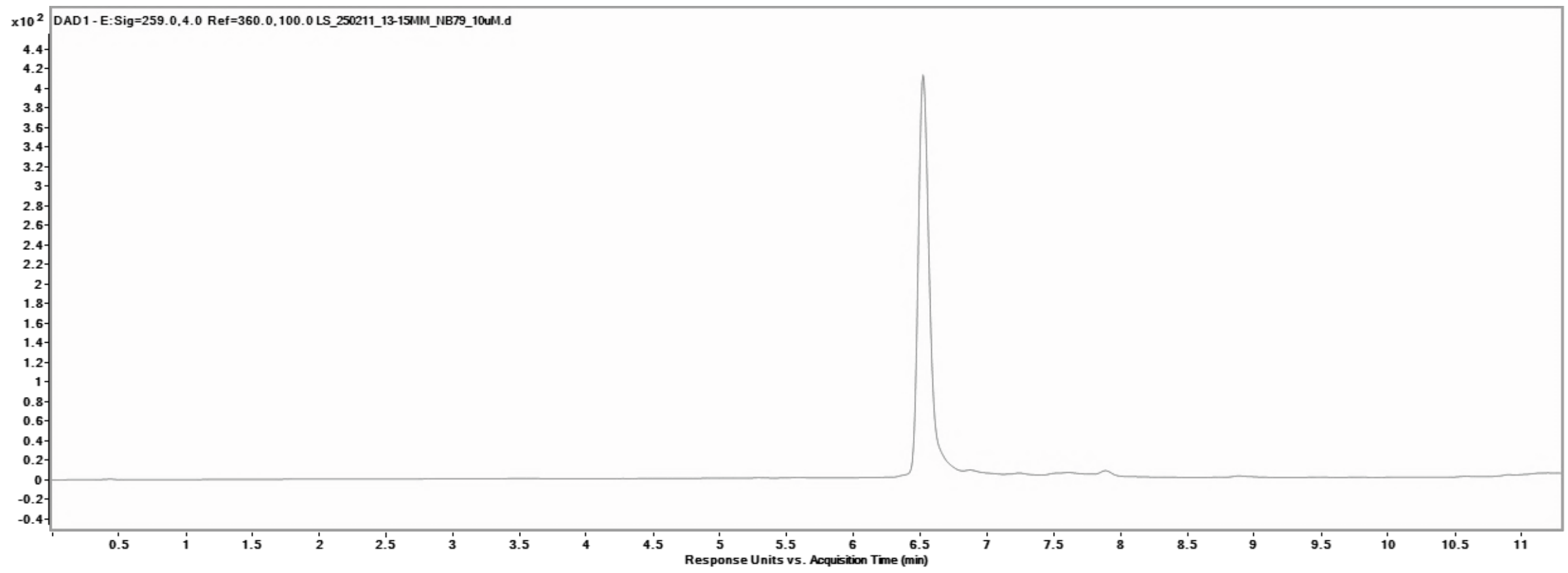

Figure S21. UV chromatogram at 259 nm of **GL-O TM13-15.**

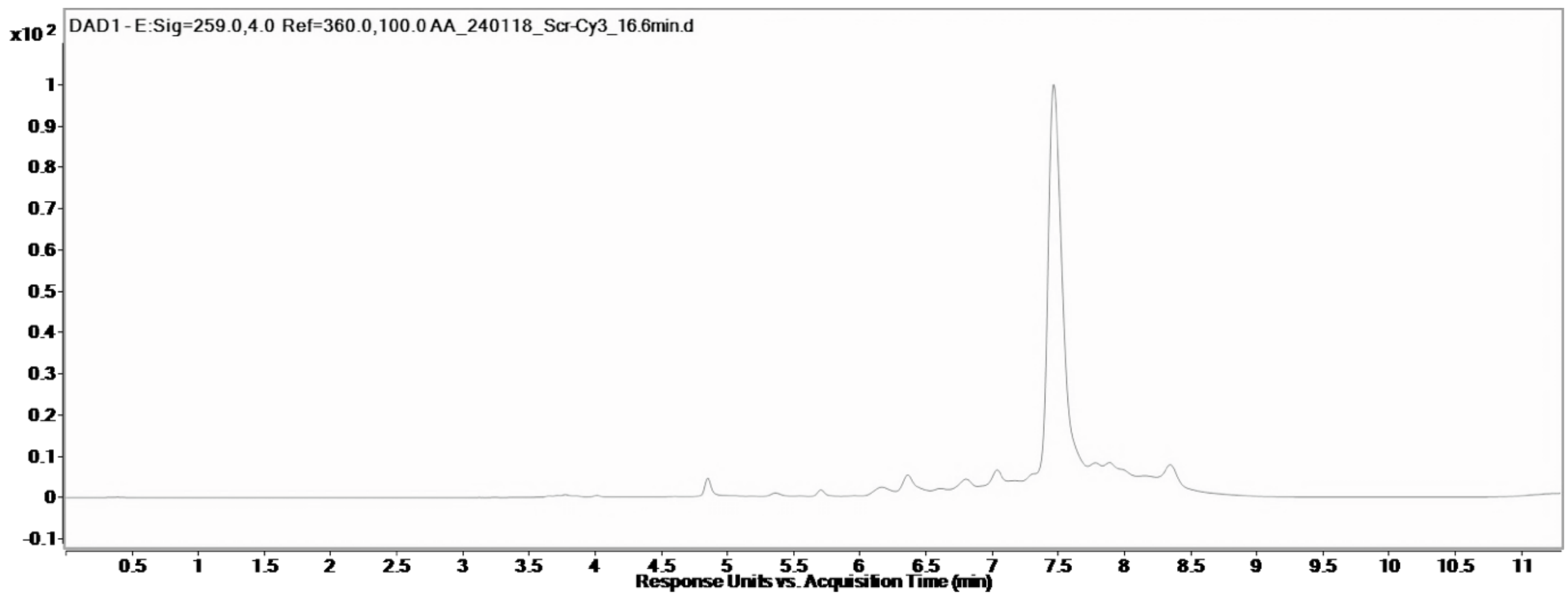

Figure S22. UV chromatogram at 259 nm of **GL-O D15-PS-Cy3-GalNAc.**

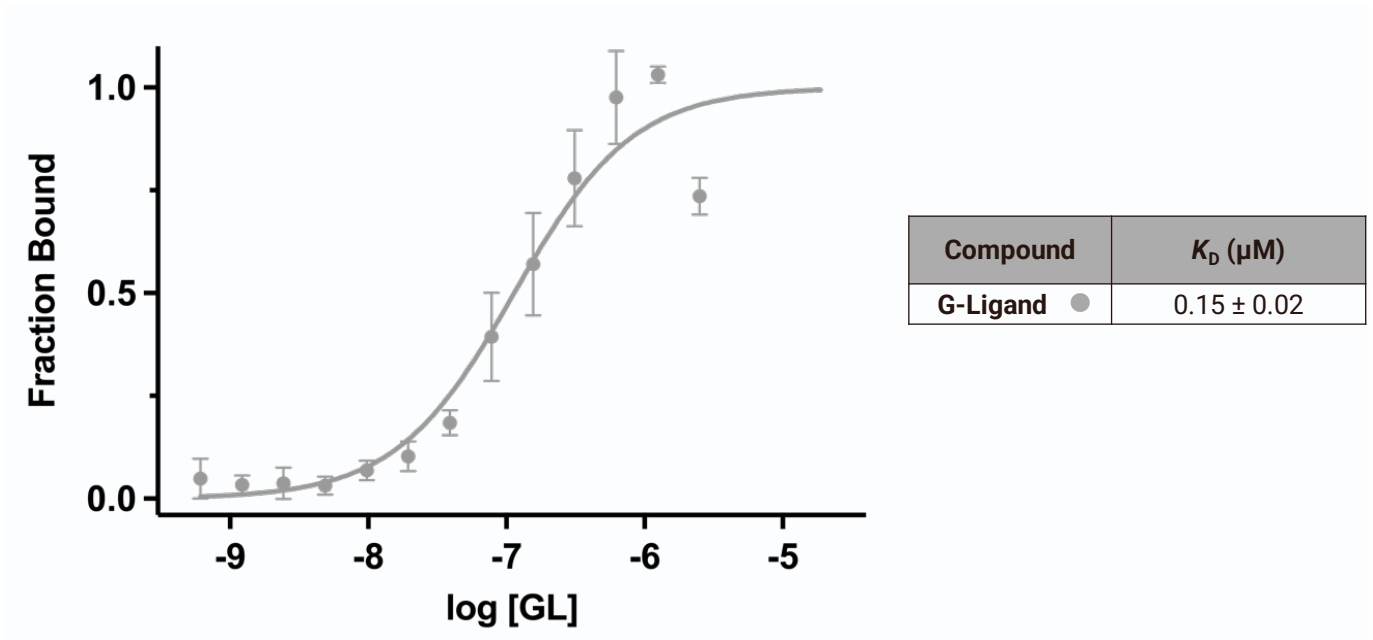

Figure S23. Binding affinity of the G4-ligand (GL). Dose-response curves obtained using MST where the GL was serial diluted to the G4 DNA 5´-labelled with a fluorescent tag. Dissociation constants (K_D) values and error bars correspond to two independent measurements.

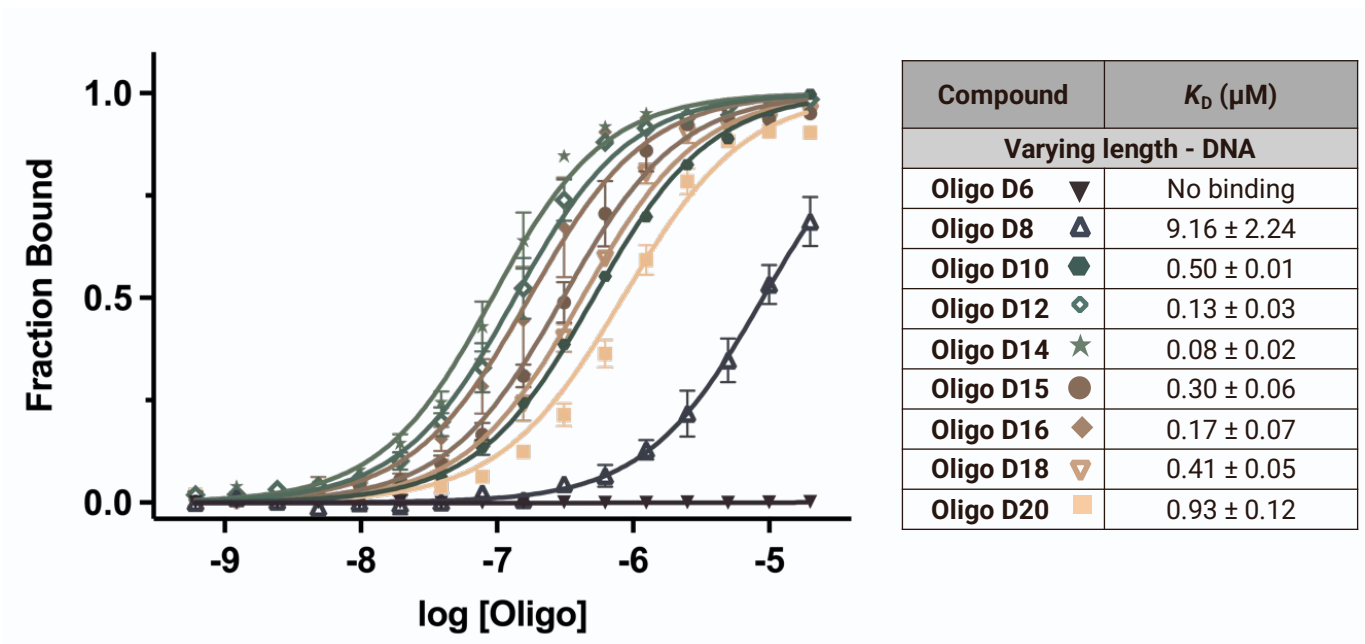

Figure S24. Binding affinities of the oligonucleotides **D6**-**D20**. Dose-response curves obtained using MST where the oligonucleotides without G4-ligands (still contains a 5´hexylamine). **D6**-**D20** were serial diluted to the G4 DNA 5´-labelled with a fluorescent tag. Dissociation constants (K_D) values and error bars correspond to two independent measurements.

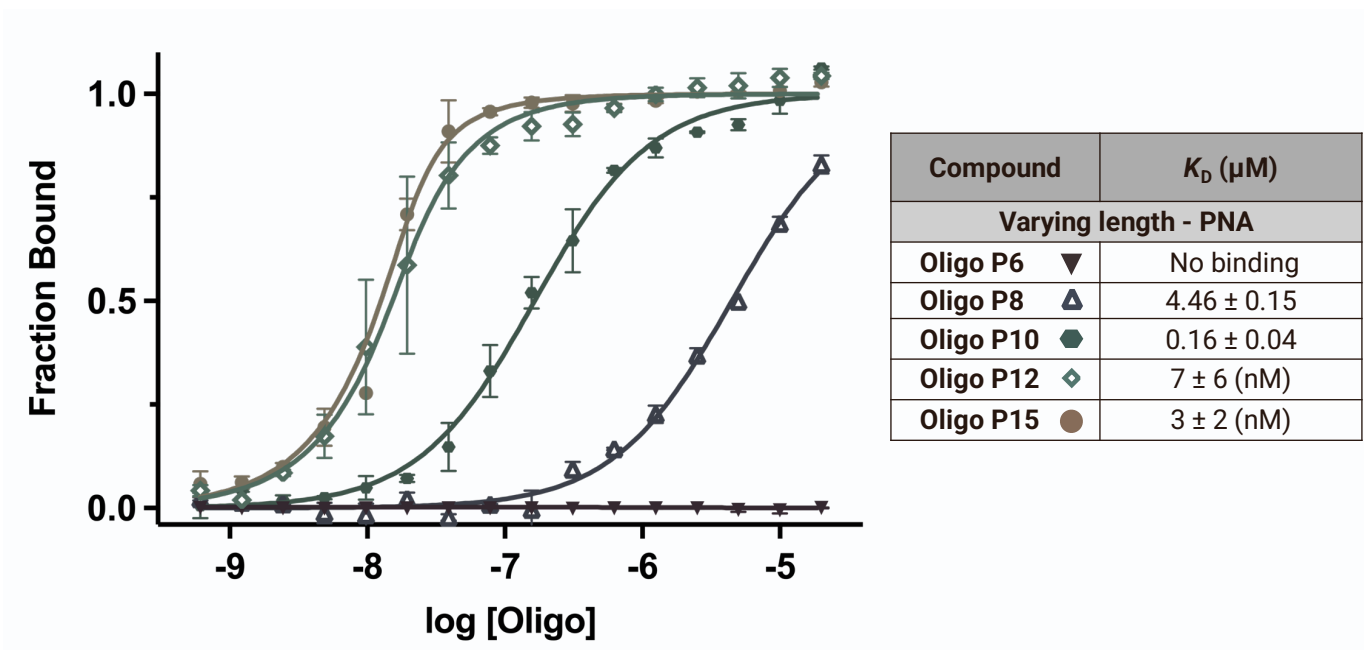

Figure S25. Binding affinities of the oligonucleotides **P6**-**P15**. Dose-response curves obtained using MST where the oligonucleotides without G4-ligands (still contains a 5´hexylamine). **P6**-**P15** were serial diluted to the G4 DNA 5´-labelled with a fluorescent tag. Dissociation constants (K_D) values and error bars correspond to two independent measurements.

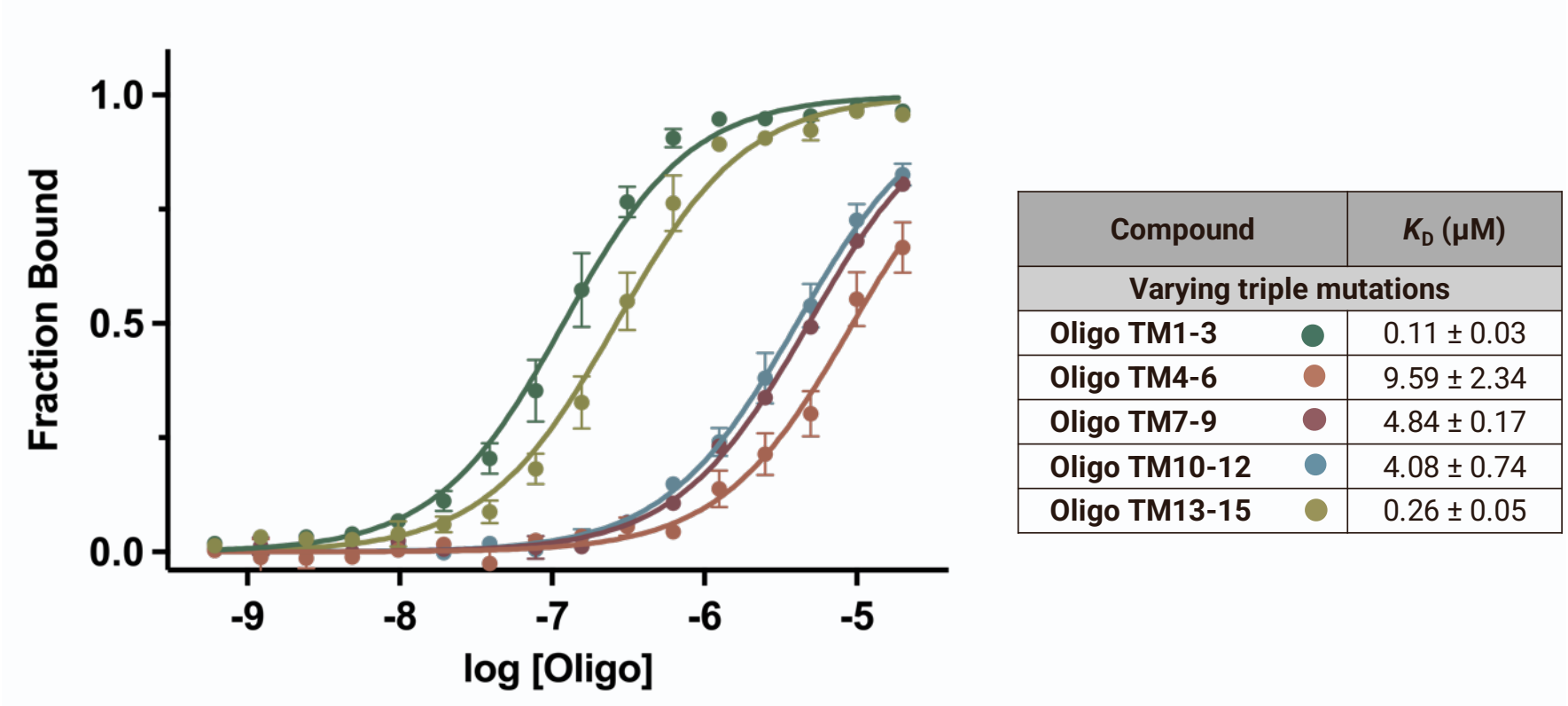

Figure S26. Binding affinities of the oligonucleotides **TM1-3, 4-6, 7-9, 10-12, 13-15.** Dose-response curves obtained using MST where the oligonucleotides without G4-ligands (still contains a 5´hexylamine). **TM1-3, 4-6, 7-9, 10-12, 13-15** were serial diluted to the G4 DNA 5´-labelled with a fluorescent tag. Dissociation constants (K_D) values and error bars correspond to two independent measurements.

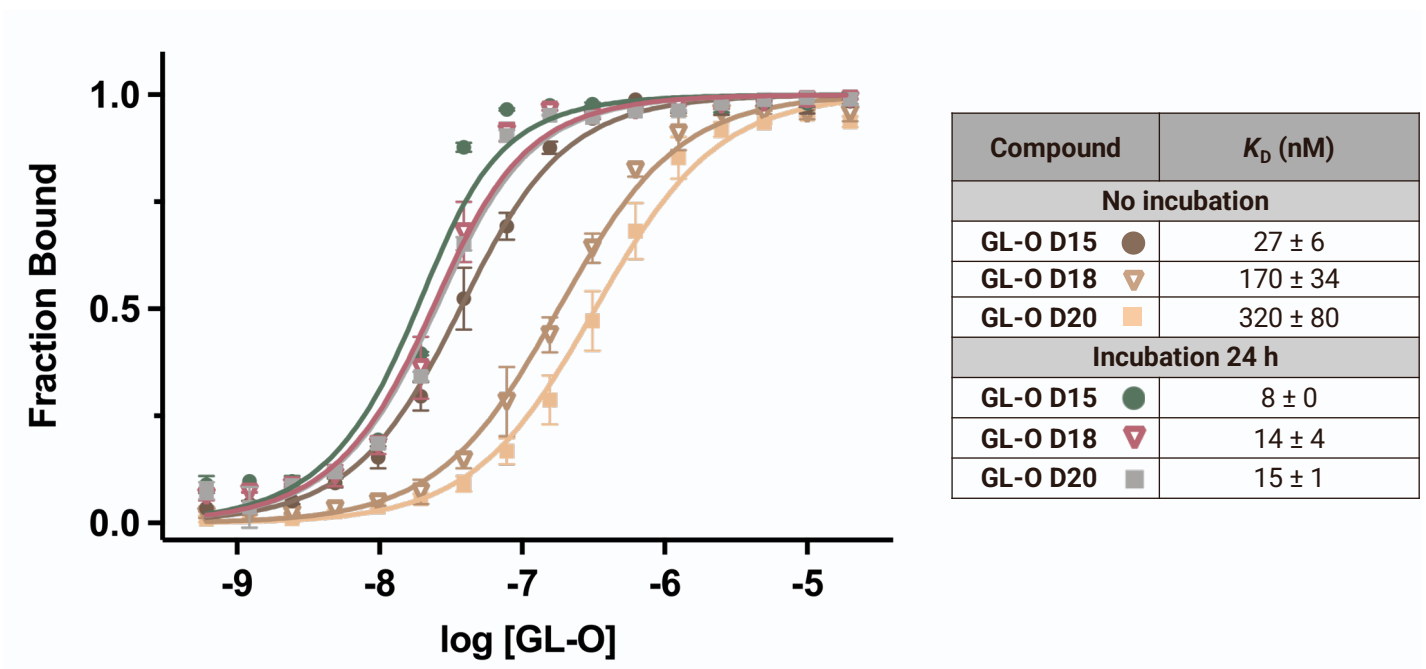

Figure S27. Binding affinities of the GL-Os **D15-D20** with or without incubation in room temperature for 24 h. Dose-response curves obtained using MST. **D15-D20** were serial diluted to the G4 DNA 5´-labelled with a fluorescent tag. Dissociation constants (K_D) values and error bars correspond to two independent measurements.

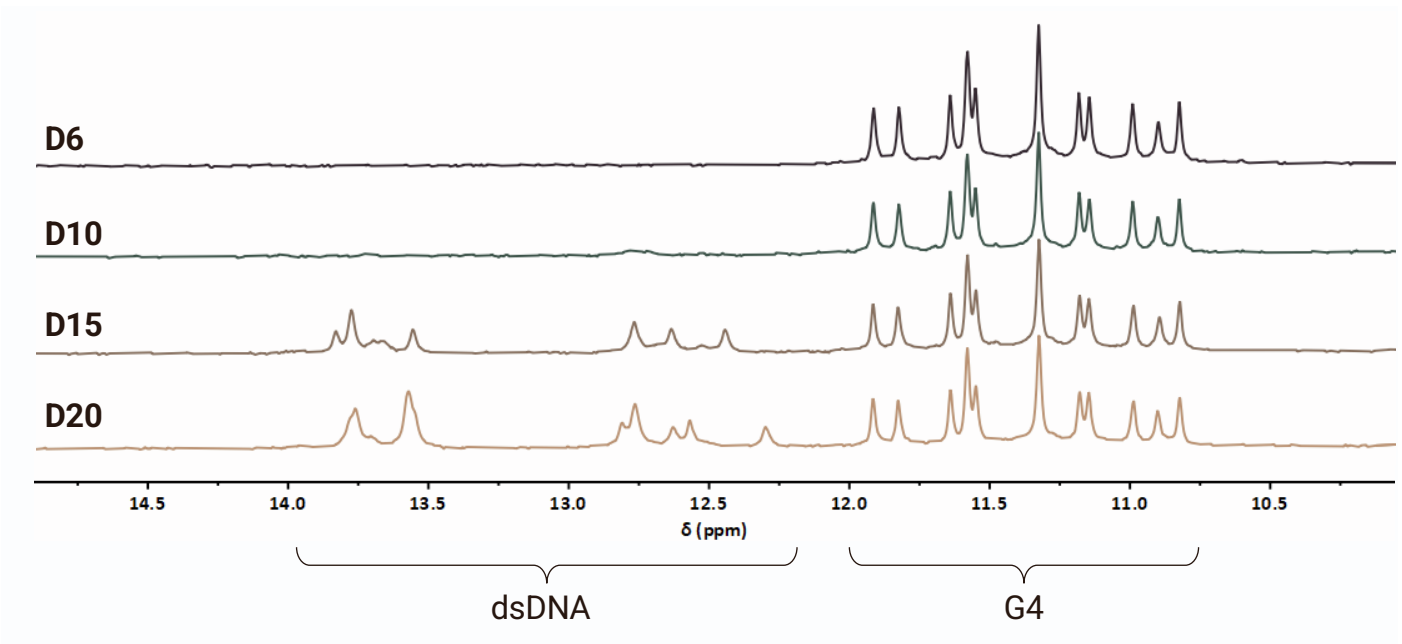

Figure S28. Binding interaction of the oligonucleotides **D6-D20** (still contains a 5´hexylamine) measure using ^{1}H NMR. Spectra were recorded of *c-MYC* Pu24T with complementary flanking sequence in a 1:1 molar ratio at 25 °C. G4 imino signals appear between 10-12 ppm and double-stranded DNA signals appear between 12-14 ppm.

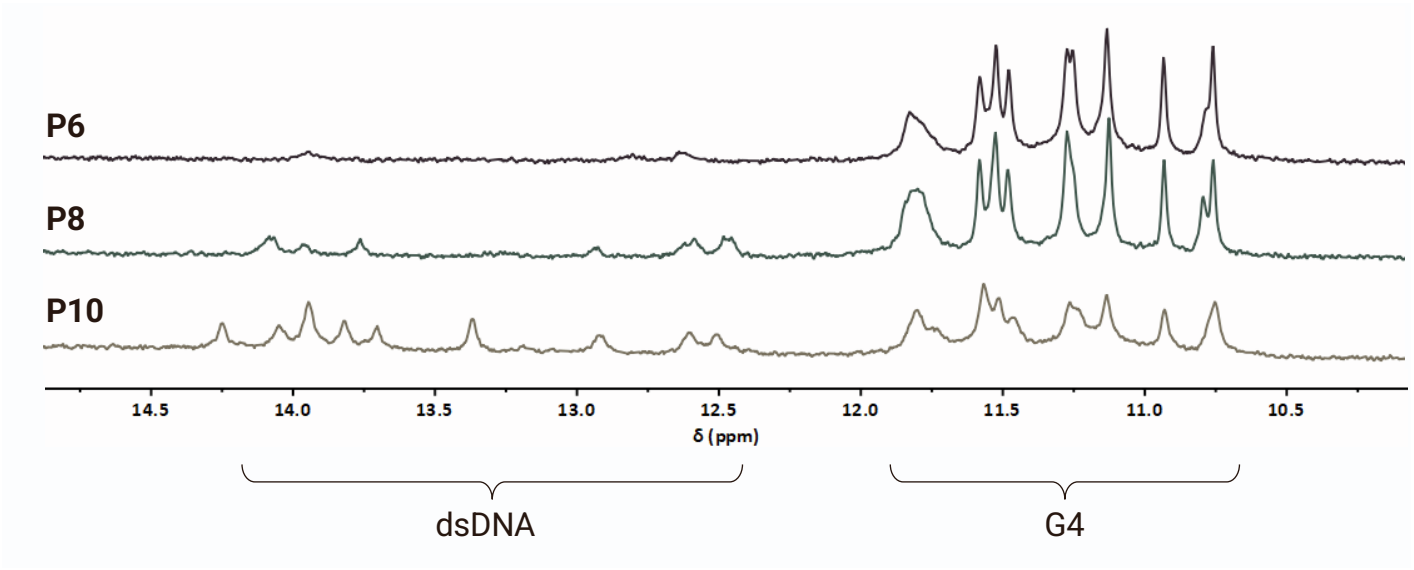

Figure S29. Binding interaction of the oligonucleotides **P6**-**P20** (still contains a 5´hexylamine) measure using ^{1}H NMR. Spectra were recorded of *c-MYC* Pu24T with complementary flanking sequence in a 1:1 molar ratio at 25 °C. G4 imino signals appear between 10-12 ppm and double-stranded DNA signals appear between 12-14 ppm.

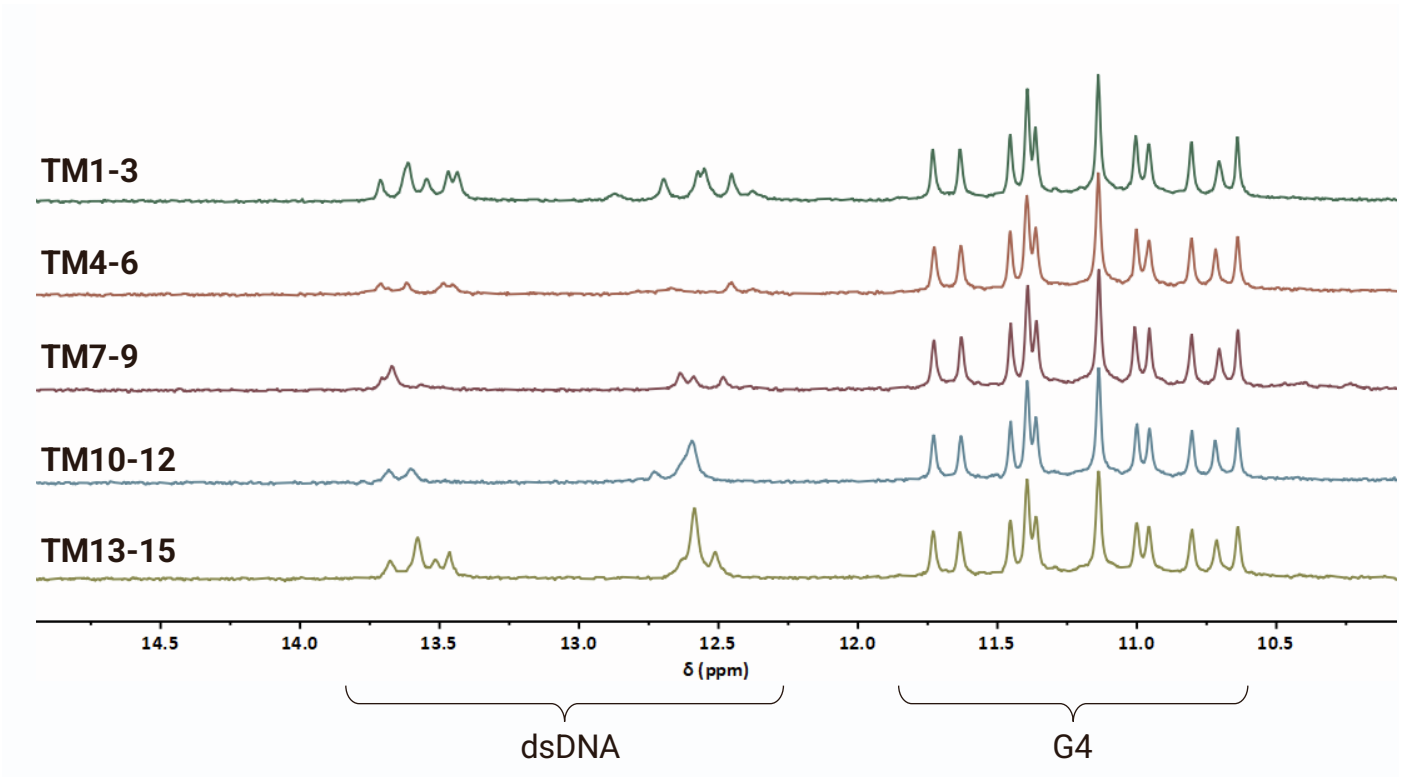

Figure S30. Binding interaction of the oligonucleotides **TM 1-3, 4-6, 7-9, 10-12, 13-15** (still contains a 5´hexylamine) measure using ^{1}H NMR. Spectra were recorded of *c-MYC* Pu24T with complementary flanking sequence in a 1:1 molar ratio at 25 °C. G4 imino signals appear between 10-12 ppm and double-stranded DNA signals appear between 12-14 ppm.

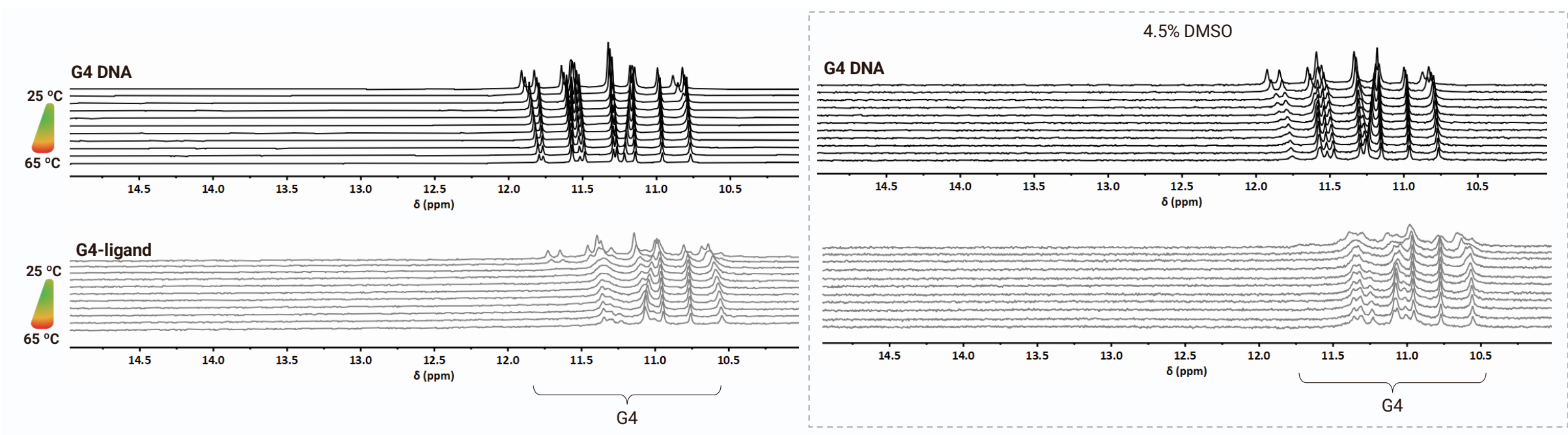

Figure S31. Thermal stabilization data of the G4 DNA alone and G4-ligand alone with and without DMSO using ^{1}H NMR. Spectra were recorded of *c-MYC* Pu24T with complementary flanking at 25 and 35 °C and then with a temperature ramp of 2.5 °C from 45-65 °C. G4 imino signals appear between 10-12 ppm and double-stranded DNA signals appear between 12-14 ppm.

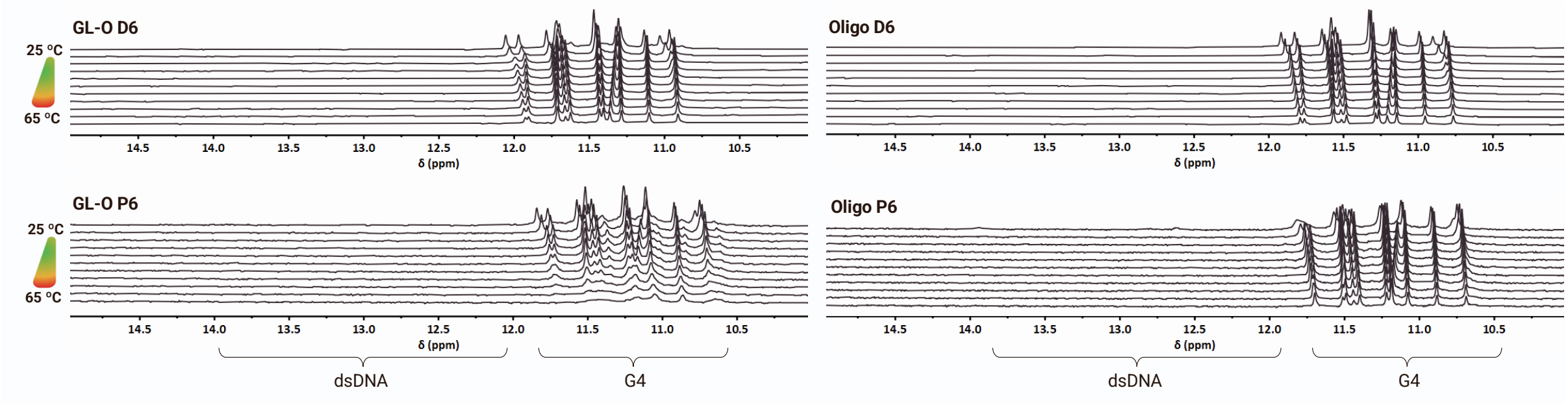

Figure S32. Thermal stabilization data of the GL-Os **D6** and **P6** with their unconjugated oligonucleotides using ^{1}H NMR. Spectra were recorded of *c-MYC* Pu24T with complementary flanking at 25 and 35 °C and then with a temperature ramp of 2.5 °C from 45-65 °C. G4 imino signals appear between 10-12 ppm and double-stranded DNA signals appear between 12-14 ppm.

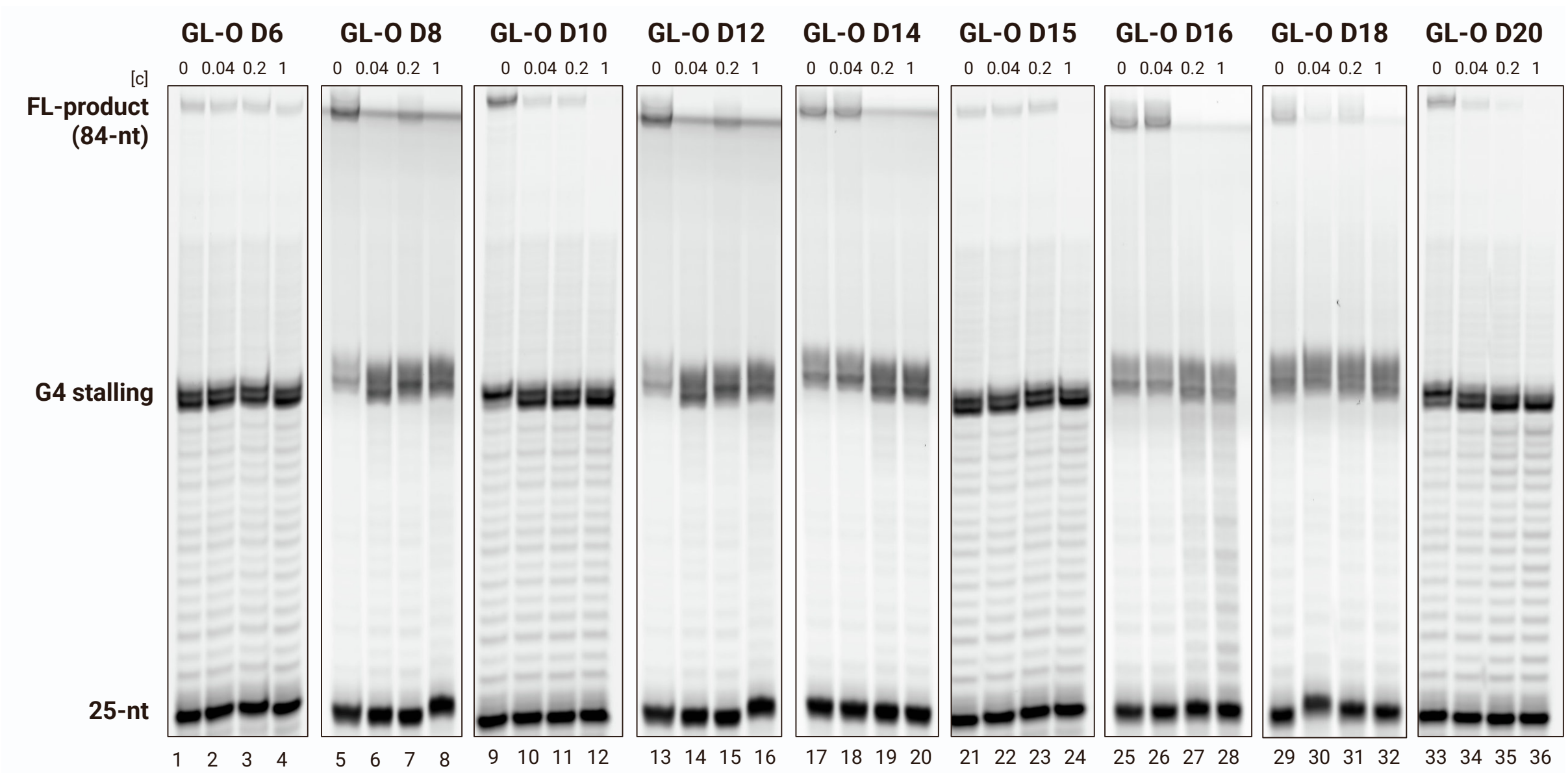

Figure S33. Taq Polymerase stabilization of the G4 DNA in presence of varying length oligonucleotide **GL-Os D6**-**D20**. Quantification of the Taq polymerase stop assay shown in as the full-length (FL, 84-nt) DNA product is expressed as a % of the full-length band intensity that was observed in the control reaction containing only the G4 template. Data represent the mean ± standard deviation from three independent experiments.

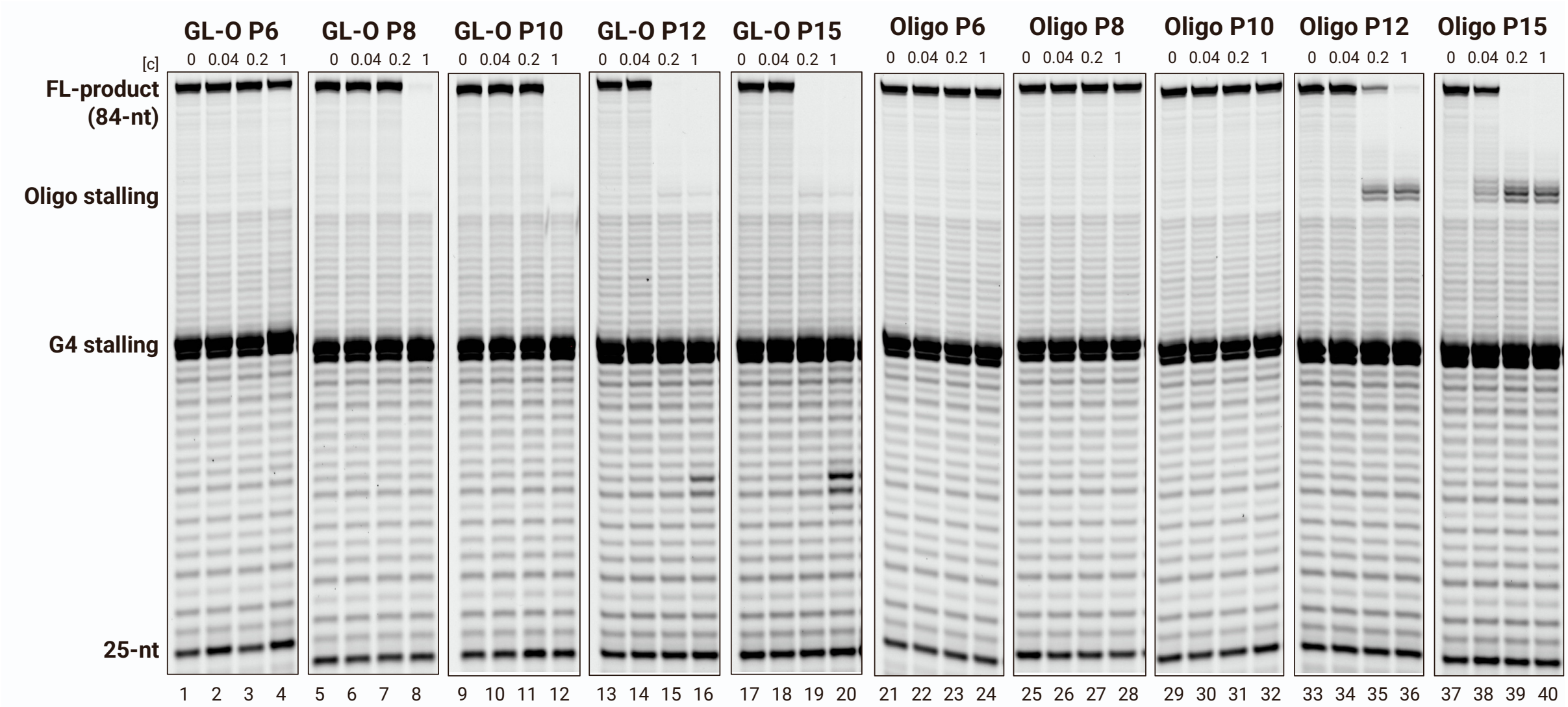

Figure S34. Taq Polymerase stabilization of the G4 DNA in presence of varying length oligonucleotide GL-Os **P6**-**P15** (lanes 1-20) and unconjugated oligonucleotides **P6**-**P15** (lanes 21-40). Quantification of the Taq polymerase stop assay shown in as the full-length (FL, 84-nt) DNA product is expressed as a % of the full-length band intensity that was observed in the control reaction containing only the G4 template. Data represent the mean ± standard deviation from three independent experiments.

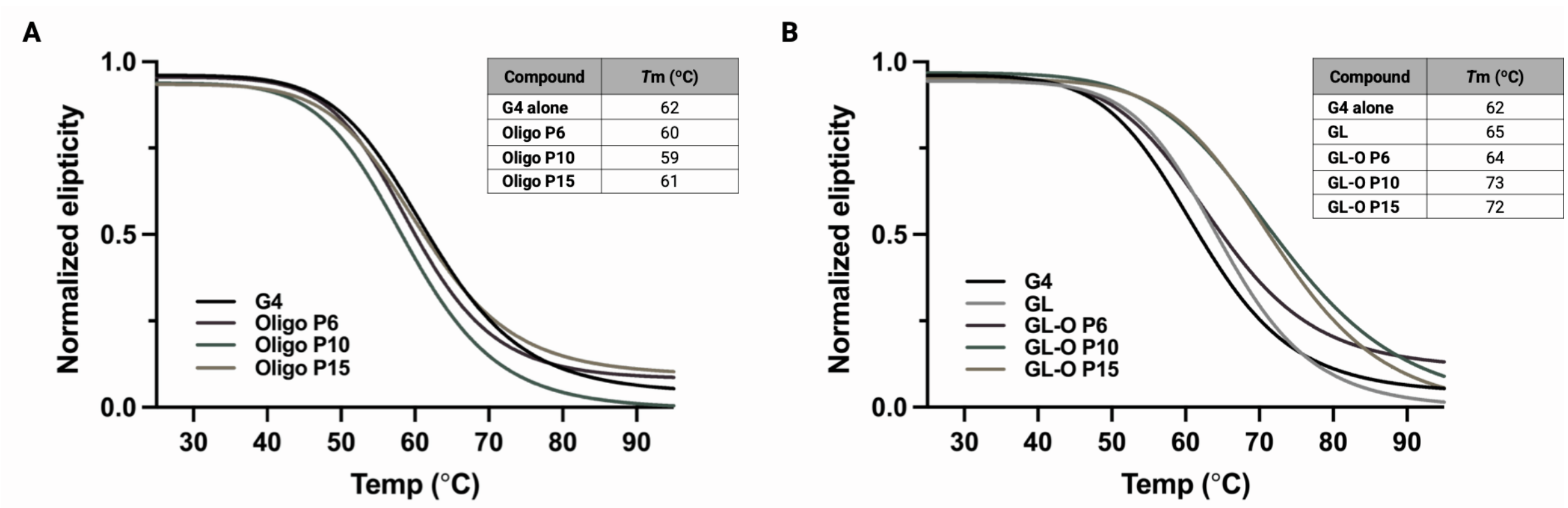

Compound	Tm (°C)
G4 alone	62
Oligo P6	60
Oligo P10	59
Oligo P15	61

Compound	Tm (°C)
G4 alone	62
GL	65
GL-O P6	64
GL-O P10	73
GL-O P15	72

Figure S35. CD melting profiles of varying length oligonucleotide GL-Os **P6**-**P15** and unconjugated oligonucleotides **P6**-**P15.** Melting profiles were measured from 25-95 °C.

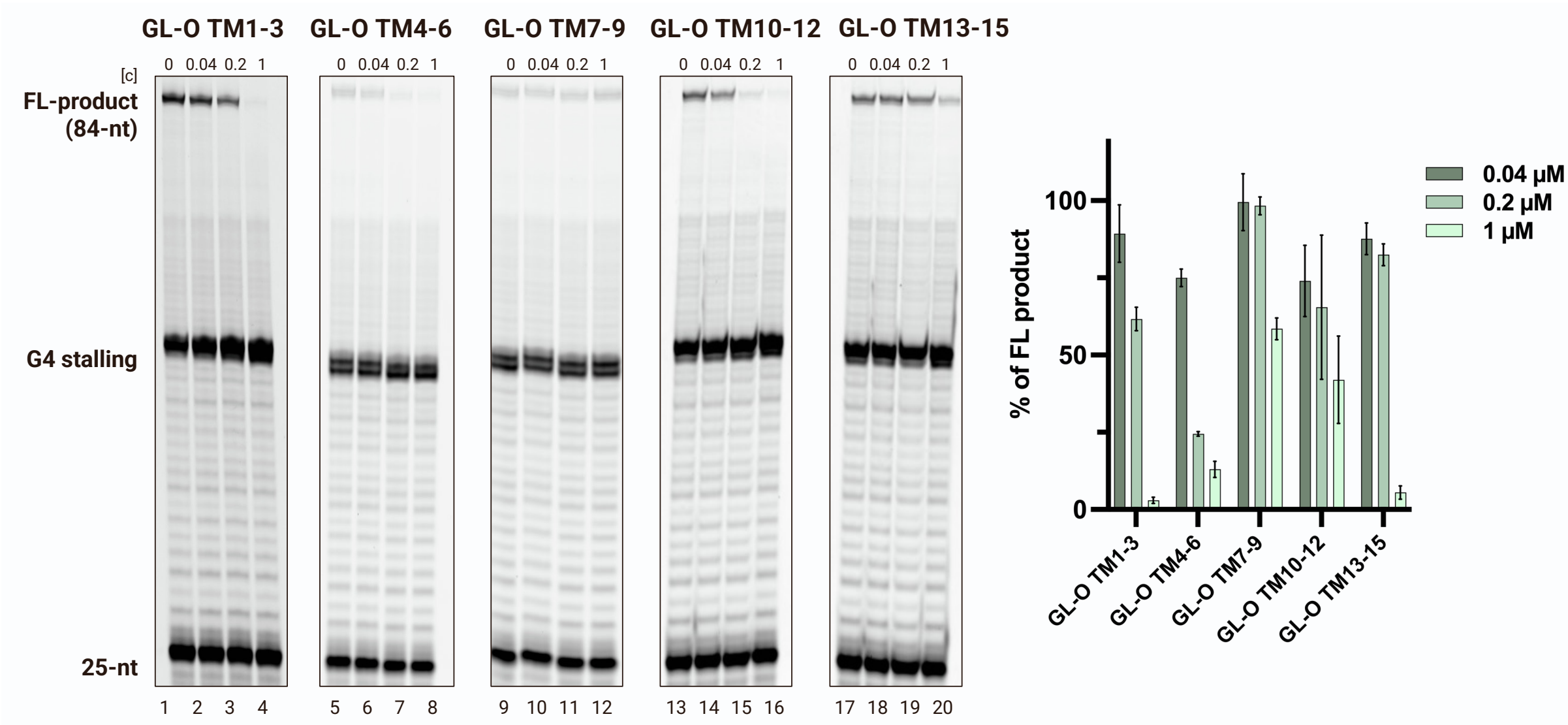

Figure S36. Taq Polymerase stabilization of the G4 DNA in presence of varying length oligonucleotide **GL-Os TM1-3**, **TM4-6**, **TM7-9**, **TM10-12** and **TM13-15**. Quantification of the Taq polymerase stop assay shown in as the full-length (FL, 84-nt) DNA product is expressed as a % of the full-length band intensity that was observed in the control reaction containing only the G4 template. Data represent the mean ± standard deviation from three independent experiments.

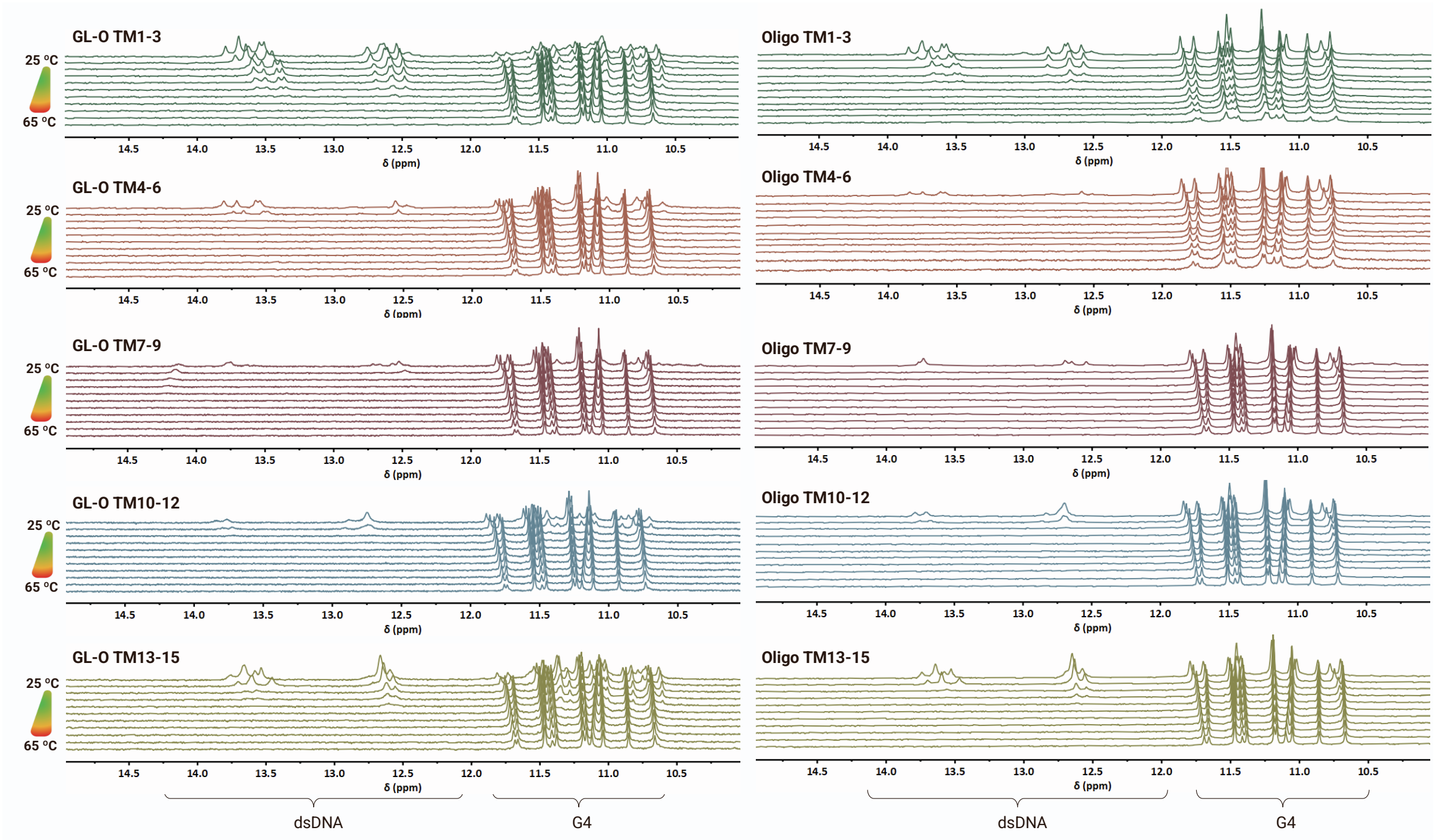

Figure S37. Thermal stabilization data of the GL-Os and unconjugated oligonucleotides **TM 1-3, TM4-6, TM7-9, TM10-12, TM13-15** using ^{1}H NMR. Spectra were recorded of *c-MYC* Pu24T with complementary flanking at 25 and 35 °C and then with a temperature ramp of 2.5 °C from 45-65 °C. G4 imino signals appear between 10-12 ppm and double-stranded DNA signals appear between 12-14 ppm.

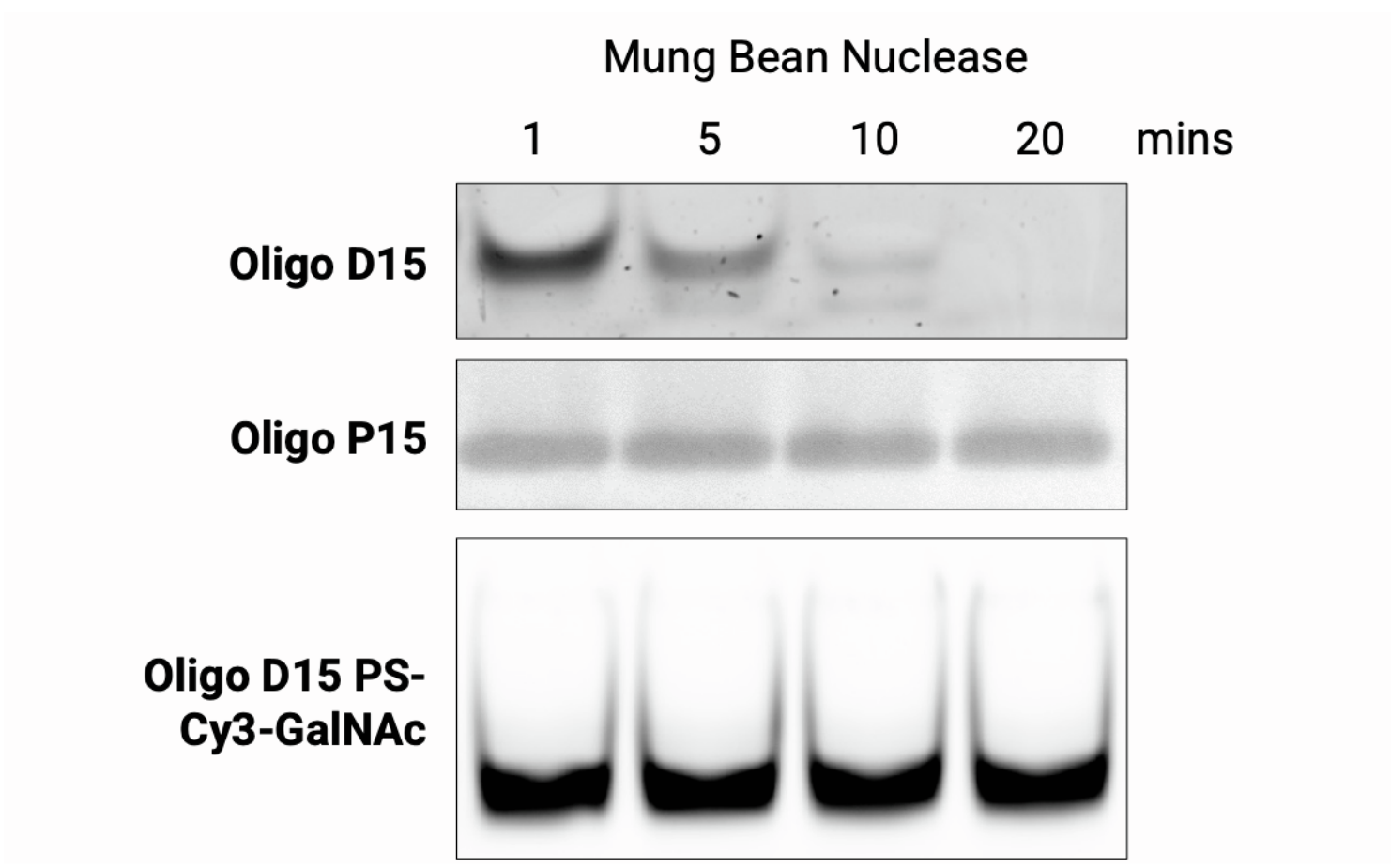

Figure S38. *In vitro* digestion of unconjugated oligonucleotides by mung bean nuclease. Unconjugated oligonucleotides were incubated with mung bean nuclease at 30 °C, and aliquots were quenched at the indicated time points. Digestion products were resolved by polyacrylamide gel electrophoresis: DNA oligonucleotides on 16% TBE gels stained with GelRed, and PNA oligonucleotides on 4–20% SDS–PAGE gels stained with InstantBlue. Gels were imaged on a ChemiDoc system (Bio-Rad).

References

1. Bhuma, N., Chand, K., Andréasson, M., Mason, J., Das, R.N., Patel, A.K., Öhlund, D., and Chorell, E. (2023). The effect of side chain variations on quinazoline-pyrimidine G-quadruplex DNA ligands. Eur. J. Med. Chem. *248*, 115103.
2. Berner, A., Das, R.N., Bhuma, N., Golebiewska, J., Abrahamsson, A., Andréasson, M., Chaudhari, N., Doimo, M., Bose, P.P., Chand, K., et al. (2024). G4-Ligand-Conjugated Oligonucleotides Mediate Selective Binding and Stabilization of Individual G4 DNA Structures. J. Am. Chem. Soc. *146*, 6926-6935.
3. Casper, J., Speir, M.L., Raney, B.J., Perez, G., Nassar, L.R., Lee, C.M., Hinrichs, A.S., Gonzalez, J.N., Fischer, C., Diekhans, M., et al. (2025). The UCSC Genome Browser database: 2026 update. Nucleic Acids Research *54*, D1331-D1335.
4. Langmead, B., and Salzberg, S.L. (2012). Fast gapped-read alignment with Bowtie 2. Nature Methods *9*, 357-359.
5. Li, H., Handsaker, B., Wysoker, A., Fennell, T., Ruan, J., Homer, N., Marth, G., Abecasis, G., Durbin, R., and Subgroup, G.P.D.P. (2009). The Sequence Alignment/Map format and SAMtools. Bioinformatics *25*, 2078-2079.
