# Supplementary material for: Dual recognition drives site-directed G-quadruplex stabilization: Oligonucleotide design in G4 ligand-oligonucleotide conjugates
Source: Mol Ther Nucleic Acids. 2026 Aug 14;37(3):103061. doi: 10.1016/j.omtn.2026.103061 (PMC13551894; doi:10.1016/j.omtn.2026.103061)
Supplement: Supplementary file 2 [file mmc2.pdf]

Molecular Therapy
Nucleic Acids
Original Article

# Dual recognition drives site-directed G-quadruplex stabilization: Oligonucleotide design in G4 ligand-oligonucleotide conjugates

Alva Abrahamsson,[1] Sakina Khwaja,[1] Namrata Chaudhari,[2] Steven Vertueux,[1] Andreas Berner,[2] Koit Aasumets,[2] Chandan Kumar,[3] Lotte Stietz,[1] Tom Baladi,[4] Anders Dahlén,[4] Sjoerd Wanrooij,[2] and Erik Chorell[1]

[1]Department of Chemistry, Umeå University, 901 87 Umeå, Sweden; [2]Department of Medical Biochemistry and Biophysics, Umea University, 907 36 Umeå, Sweden; [3]Chemical Biology Consortium Sweden (CBCS), Department of Chemistry, Umeå University, 901 87 Umeå, Sweden; [4]Nucleic Acid Therapeutics, Discovery Sciences, Biopharmaceuticals R&D - AstraZeneca Gothenburg, 431 83 Mölndal, Sweden

**G-quadruplex (G4) DNA structures are increasingly recognized for their roles in transcriptional regulation and genome stability, making them attractive therapeutic targets. Selective recognition of individual G4s remains challenging due to the high structural similarity among G4 motifs. G4 Ligand-Oligonucleotides conjugates (GL-Os) address this challenge by combining small-molecule G4 ligands with the sequence specificity of oligonucleotides, targeting sequences flanking the intended G4 target. Here, we systematically investigate how oligonucleotide length, backbone composition, and sequence complementarity govern GL-O binding, selectivity, and G4 stabilization. We show that effective G4 recognition depends on the interdependence between oligonucleotide hybridization and G4 ligand binding, such that both elements cooperatively reinforce complex stability and site specificity. Longer oligonucleotides promote more stable complexes and stronger G4 stabilization, whereas central mismatches disrupt this dual-recognition mechanism. Replacement of DNA with peptide nucleic acids (PNAs) enhances binding strength, thermal stability, and metabolic stability. Importantly, ligand conjugation redirects PNA oligonucleotides from nonspecific polymerase stalling toward selective G4 stabilization. Finally, we demonstrate receptor-mediated cellular uptake of modified GL-Os, supporting the feasibility of cellular delivery while highlighting remaining delivery barriers. Together, these findings show the molecular design principles governing GL-O behavior and provide a foundation for the future development and evaluation of selective G4-targeting therapeutics.**

## INTRODUCTION

G-quadruplex (G4) DNA structures are non-canonical secondary conformations that form either intra- or intermolecularly within guanine-rich regions of the genome. These structures are characterized by the formation of G-tetrads, which are planar arrangements of four guanine bases stabilized by Hoogsteen hydrogen bonding. G-tetrads can self-stack through arene-arene interactions and are further stabilized by monovalent or divalent cations, such as $Na^+$ or $K^+$.[1] G4 structures exhibit significant topological polymorphism, with their conformation influenced by factors, such as strand orientation, loop length, and the nature of the stabilizing cation.[2]

G4 formation within promoter regions has been greatly investigated through *in vitro* and cellular assays, where these structures have demonstrated a direct role in transcriptional regulation.[3,4] Among the most thoroughly studied examples is the *MYC* oncogene, which encodes the transcription factor MYC, and this G4 is of particular interest due to its potential as a therapeutic target in cancer.[5] The guanine-rich nuclease hyper-sensitivity element $III_1$ is responsible for the majority of the *MYC* transcription,[5] which contains a well-characterized G4-forming sequence termed Pu27. Small molecules, referred to as G4 ligands, have been shown to bind and stabilize this G4 structure, resulting in downregulation of *MYC* expression.[6] However, many G4 ligands act on multiple genomic sites, and the observed effects may reflect broader, genome-wide G4 stabilization rather than a promoter-specific response.[7,8] This complexity is compounded by the potential formation of over 700,000 G4 structures throughout the human genome,[9] with the G-tetrad as a conserved structural motif serving as the primary-binding site for most G4-targeting ligands. As a result, there is a pressing need for strategies that enable selective targeting of individual G4 structures, to advance the developments of therapeutic agents and elucidating the cellular functions of G4s.

To achieve sequence-specific G4 recognition, one promising approach involves conjugating a G4-specific ligand to an oligonucleotide designed to be complementary to the flanking sequence of the target G4. This design guides the ligand to the intended G4 structure while minimizing off-target interactions with non-matching G4s.[10]

Received 8 February 2026; accepted 11 August 2026;
https://doi.org/10.1016/j.omtn.2026.103061.

**Correspondence:** Sjoerd Wanrooij, Department of Medical Biochemistry and Biophysics, Umea University, 907 36 Umeå, Sweden.
**E-mail:** sjoerd.wanrooij@umu.se
**Correspondence:** Erik Chorell, Department of Chemistry, Umeå University, 901 87 Umeå, Sweden.
**E-mail:** erik.chorell@umu.se


© 2026 The Author(s). Published by Elsevier Inc. on behalf of The American Society of Gene and Cell Therapy.
This is an open access article under the CC BY license (http://creativecommons.org/licenses/by/4.0/).

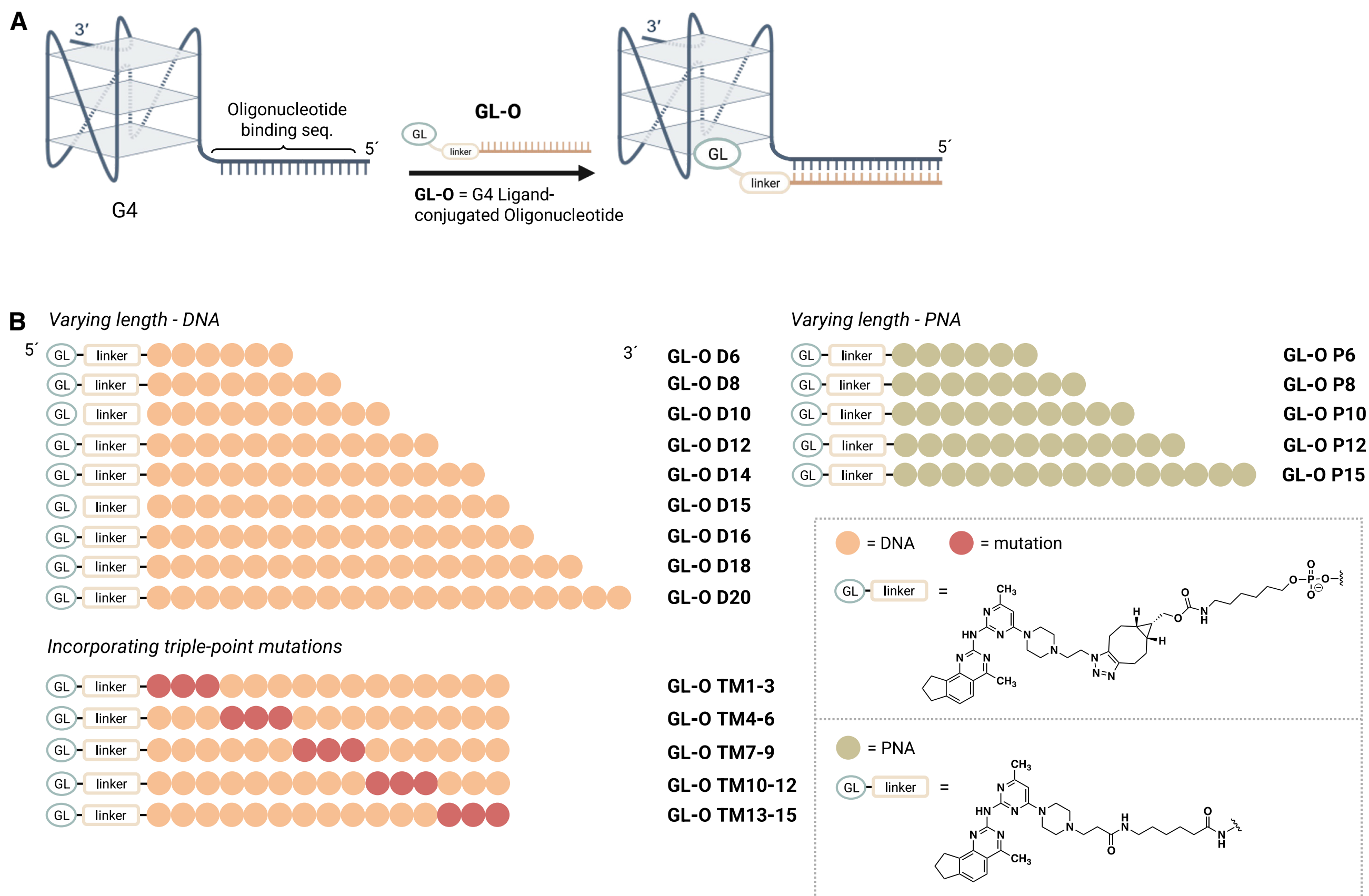


**Figure 1. Overview of the GL-O strategy and the GL-O conjugates investigated in this study**

(A) Overview of the G4 Ligand-conjugated oligonucleotide (GL-Os) strategy and how the oligonucleotide binds to the 5′-flanking sequence of the G4. Schematic made with Biorender. (B) Modified GL-Os based on oligonucleotide length (**GL-Os D6**–**D20**), backbone composition (**GL-Os P6**–**P15**), and systematically introduced triple-point mutations (**GL-Os TM1–3**, **TM4–6**, **TM7-9**, **TM10–12**, and **TM13–15**).

This approach is referred to as the G4 Ligand-conjugated oligonucleotide (GL-O) strategy (Figure 1A).[10]

Previous studies on the GL-O strategy have included detailed investigations into both the linker composition[11] and the G4 ligand,[12] resulting in a flexible platform capable of accommodating various conjugation designs and fine-tuning the G4 ligand for optimal GL-O potency. Building on this foundation, the present study focuses on the primary determinant of the GL-O selectivity: the oligonucleotide. Specifically, we examine how three parameters, the oligonucleotide length, chemical backbone modifications, and the degree of sequence complementarity, affect binding affinity, structural properties, and the ability to stabilize a target G4 DNA structure.

First, we investigated the effect of oligonucleotide length. The original GL-O design incorporated a 15-nucleotide (nt) oligonucleotide, which was selected based on foot printing experiments that identified a 14–15 nt single-stranded DNA region in the 5′-flanking sequence of the *c-MYC* promoter.[13] However, a systematic evaluation of optimal oligonucleotide length has not yet been conducted. To address this, we performed sequence alignments of various length oligonucleotides with three known G4 targets. This led us to design a series of GL-O constructs with oligonucleotide lengths ranging from 6 to 20 nt (Figure 1B).

Second, we evaluated how oligonucleotide composition influences GL-O performance. While selectivity requires sufficient length, this must be balanced against hybridization stability. To modulate these properties, we incorporated peptide nucleic acid (PNA), a DNA mimic with a neutral, peptide-like backbone developed by the Nielsen group in 1991.[14] This uncharged backbone eliminates electrostatic repulsion during hybridization with the complementary DNA target, resulting in significantly enhanced binding affinity compared to unmodified DNA.[15] In addition to their enhanced binding affinity, PNA oligonucleotides demonstrate pronounced metabolic stability against nuclease-mediated degradation.[15] We therefore created a series of PNA-based GL-Os with oligonucleotide lengths ranging from 6 to 15 nt in length (Figure 1B).

Third, we assess the influence of sequence complementarity. A previous study examined the effects of central single-point mutations

**Table 1. Sequence conservation analysis of complementary sequences within the genome for three different G4 targets**

| Entry | Oligonucleotide length | Numbers of G4 flanking sequence sites in human genome | | |
|---|---|---|---|---|
| | | *c-Myc* Pu27 | *KRAS* 32R | *Helicase B* G4-1 |
| 1 | 20 | 1 | 1 | 1 |
| 2 | 19 | 1 | 1 | 1 |
| 3 | 18 | 1 | 1 | 1 |
| 4 | 17 | 1 | 1 | 1 |
| 5 | 16 | 1 | 1 | 6 |
| 6 | 15 | 1 | 2 | 16 |
| 7 | 14 | 1 | 3 | 54 |
| 8 | 13 | 4 | 74 | 309 |
| 9 | 12 | 15 | 210 | 662 |
| 10 | 11 | 78 | 788 | 1,838 |
| 11 | 10 | 330 | 2,141 | 7,228 |
| 12 | 9 | 1,816 | 8,119 | 35,586 |
| 13 | 8 | 56,285 | 22,049 | 94,195 |
| 14 | 7 | 253,973 | 59,166 | 455,120 |
| 15 | 6 | 1,556,186 | 250,643 | 1,639,320 |
| 16 | 5 | 5,065,989 | 1,021,355 | 5,697,262 |

and double-point mutations in the peripheral regions.[10] However, a more systematic analysis is still needed to identify clear trends in mismatch tolerance. To address this, we designed five GL-Os containing triple-point mutations at various positions along the oligonucleotide sequence (Figure 1B).

By systematically varying these three parameters, we aim to define the molecular principles governing G4 recognition and stabilization by GL-Os.

## RESULT

### Sequence conservation analysis of potential oligonucleotide lengths for various G4 targets

The length of the oligonucleotide is a key factor determining the selectivity of the GL-O strategy. A sufficiently long sequence enables the oligonucleotide to hybridize selectively to the desired genomic target while avoiding partial matches elsewhere. To evaluate how oligonucleotide length influences selectivity, we first aligned our target oligonucleotide binding sequences against the complete human genome. Genomic regions showing sequence similarity, with no mismatches allowed, were then annotated to determine their genomic locations and whether they resided within coding or non-coding regions.

The analysis indicates that the required length of the oligonucleotide differs depending on the G4 target (Table 1). For the *c-MYC* Pu27 sequence, individual specificity could be retained with a 14-nt oligonucleotide, whereas the corresponding 13-nt variant showed complementarity to three additional genomic loci (listed in Table S5), none of which were adjacent to predicted G4-forming regions. In contrast, 13-nt sequences derived from *KRAS* and *Helicase B* exhibited much lower selectivity, matching 74 and 309 genomic locations, respectively.

This analysis considers only complete complementarity to the oligonucleotide guide sequence. Sequences containing one or a few mismatches are expected to occur more frequently in the genome and may still retain partial binding affinity, although typically with reduced affinity compared to fully complementary targets. Furthermore, within the GL-O strategy, only sequences located in proximity to a G4 structure would be expected to result in true off-target effects.

### Design of oligonucleotide-modified GL-Os

The chemical design of the GL-O conjugate builds on previous work in which the G4 ligand and the linker chemistry were extensively investigated. In the present study, we retained the same G4 ligand as described in the initial report of the GL-O strategy.[10] Conjugation was achieved through a strain-promoted azide-alkyne cycloaddition reaction, coupling an azide-functionalized G4 ligand with an oligonucleotide carrying a 5′-bicyclononyne (BCN).[11] This approach provides a robust, copper-free biorthogonal linkage that ensures efficient conjugations.

For the DNA-based GL-Os, a series of oligonucleotides of varying lengths (6, 8, 10, 12, 14, 15, 16, 18, and 20 nt) were synthesized and conjugated to the G4 ligand to generate **GL-Os D6–D20** (see Table S3 for sequences and Table S4 for yields).

To evaluate the effect of backbone composition, a corresponding set of PNA-based GL-Os with different length oligonucleotides (6, 8, 10, 12, and 15 nt), named **GL-Os P6–P15**, were synthesized (Figure 2, more detailed in Figure S1). The PNA backbone oligonucleotide was synthesized on an automated solid-phase peptide synthesizer. PNA elongation was carried out in accordance with standard procedures.[16] Conjugation of the G4 ligand to the N terminus was performed directly on solid support under amide coupling conditions using the carboxylic acid analog of the G4 ligand previously described (Figure 2).[11] Subsequent deprotection and cleavage using trifluoroacetic acid (TFA)/m-cresol yielded the purified **GL-Os P6–P15** (see Table S3 for sequences; Table S4 for yields).

Finally, to probe the role of sequence complementarity, a series of triple-point mutation GL-Os were designed based on the 15-nt oligonucleotide. In these GL-Os, three consecutive bases were replaced with thymine, while thymine residues within the triple mutated region were substituted with cytidine to maintain overall base composition. The resulting variants (**GL-Os TM1–3**, **TM4–6**, **TM7–9**, **TM10–12**, and **TM13–15**) allow systematic evaluation of how mismatch position affects hybridization and target selectivity (see Table S3 for sequences; Table S4 for yields).

### Binding properties of the oligonucleotide-modified GL-Os

The G4 target selected for this study was the *c-MYC* G4, known as Pu24T, a stabilized mutant of the native Pu27 sequence. This

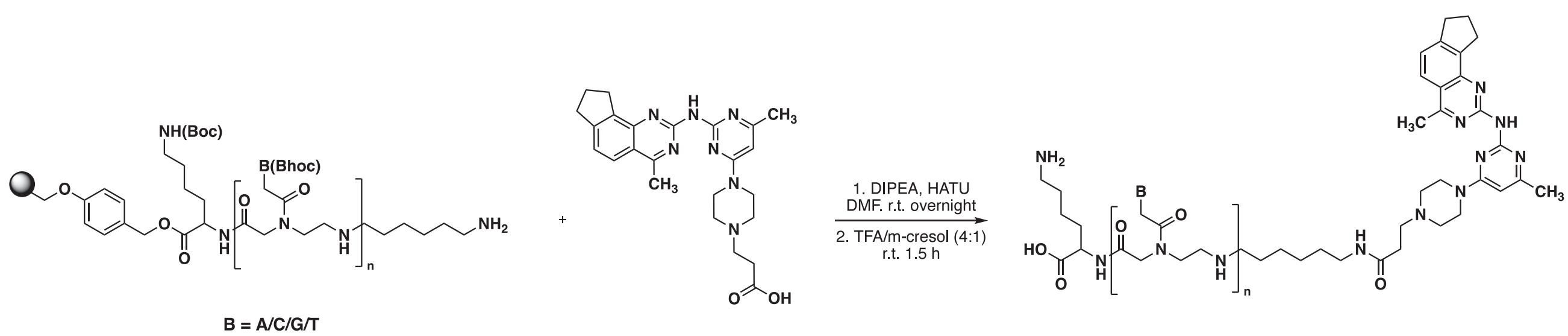


**Figure 2. Synthesis of PNA GL-Os P6–P15 (*n* = 6–15 nt)**

construct includes the G4-forming sequence together with an adjacent accessible 20-nt 5′-flanking sequence complementary to the oligonucleotide component of the GL-Os (Table S2; Figure 1). Throughout this study, the term G4 DNA refers specifically to the *c-MYC* Pu24T sequence along with this 5′-flanking sequence.

Binding of the GL-Os to the *c-MYC* G4 target was characterized by microscale thermophoresis (MST) and proton nuclear magnetic resonance ($^1$H-NMR) spectroscopy. MST provided dissociation constants ($K_D$) from dose-response curves with fluorescently labeled G4 DNA (Table S2), while NMR monitored imino proton resonances to detect G4 interactions (10–12 ppm) and duplex formation (12–14 ppm). As controls, the G4 ligand alone and the individual unconjugated oligonucleotides were analyzed in both MST and NMR experiments (Figures 3, S23–S26, and S28–S30). Together, these complementary techniques allowed quantitative and structural assessment of GL-O binding.

### *Effect of oligonucleotide length*

A clear relationship between oligonucleotide length and binding affinity was observed for the DNA GL-Os. MST analysis showed that conjugates with oligonucleotides shorter than 8 nt displayed negligible binding, indicating that 8 nt represents the minimal effective length required for hybridization under these conditions (Figure 3A). Within the range of 8–16 nt, the DNA-based **GL-Os D8**–**D16** displayed comparable affinities, with $K_D$ values ranging from 15 to 37 nM, suggesting similar binding efficiency within this range. Unexpectedly, longer oligonucleotides in the **GL-Os D18** and **D20** exhibited reduced binding affinity, with $K_D$ values increasing to 170 and 320 nM, respectively. This reduction may reflect slower hybridization kinetics. Consistent with this interpretation, extended incubation (24 h) decreased the $K_D$ values for **GL-Os D18** and **D20**, suggesting that full hybridization requires additional time for longer oligonucleotides in the GL-Os (Figure S27).

NMR spectroscopy corroborated these findings (Figure 3A). Increasing oligonucleotide length in GL-Os from 6 to 20 nt resulted in the appearance of imino proton signals between 12 and 14 ppm, corresponding to double-stranded DNA (dsDNA) formation. The G4 imino proton signals between 10 and 12 ppm were retained for all GL-Os, although binding of the G4 ligand affected the G4 structure leading to the appearance of additional peaks in this region, consistent with previous reports.[10,11] Extension beyond 10 nt did not further alter the G4-specific imino resonances, indicating that once a minimal hybridization interface is established, additional base pairs do not affect the interaction between the G4 and the G4 ligand. The shortest construct (**GL-O D6**) showed no detectable hybridization and minimal perturbation of the G4 imino signals, consistent with its weak binding observed by MST and the behavior of the unconjugated oligonucleotide (Figure S28).

### *Effect of backbone composition*

MST analysis revealed that substitution of the DNA backbone with PNA enhanced binding affinity (Figure 3B). For equivalent lengths, the PNA-based GL-Os consistently exhibited lower $K_D$ values than their DNA counterparts, although the magnitude of the affinity enhancement under these MST conditions was moderate. For instance, **GL-O P15** showed stronger binding affinity than **GL-O D15**. This increase in affinity is consistent with the neutral, peptide-like PNA backbone, which eliminates electrostatic repulsion between the oligonucleotide and the DNA target and thereby stabilizes the duplex.

NMR spectroscopy supported these findings (Figure 3B) and additionally revealed the lower aqueous solubility of the PNA constructs, which required an increased DMSO content in the NMR experiments (4.5%). This change in solubility gave minor alterations of the G4 imino protons with a separation of G-8/9 and G-18 and a merge of G-15 and G-17 (Figure 3B). The NMR showed that PNA-based GL-Os displayed more pronounced broadening and partial shifting of the G4 imino proton signals (10–12 ppm), compared with the DNA analogs, indicating stronger or more persistent interactions with the G4 structure. Signals corresponding to dsDNA regions (12–14 ppm) were also prominent, consistent with extensive hybridization. However, similar to the DNA **GL-O D6**, the shortest PNA **GL-O P6** failed to hybridize and showed only minimal effects on the G4 signals. Interestingly, the unconjugated PNA oligonucleotides alone induced small shifts in the G4 imino region (Figure S29), reflecting their inherent effect on the surrounding environment. However, when conjugated to the G4 ligand, the resulting GL-Os produced broader and more intense perturbations of the G4 signals, implying that the G4 ligand and the PNA act cooperatively to stabilize the complex. Furthermore, both **GL-Os P10** and **P15** gave strong broadening of the G4 signals, which was not evident from

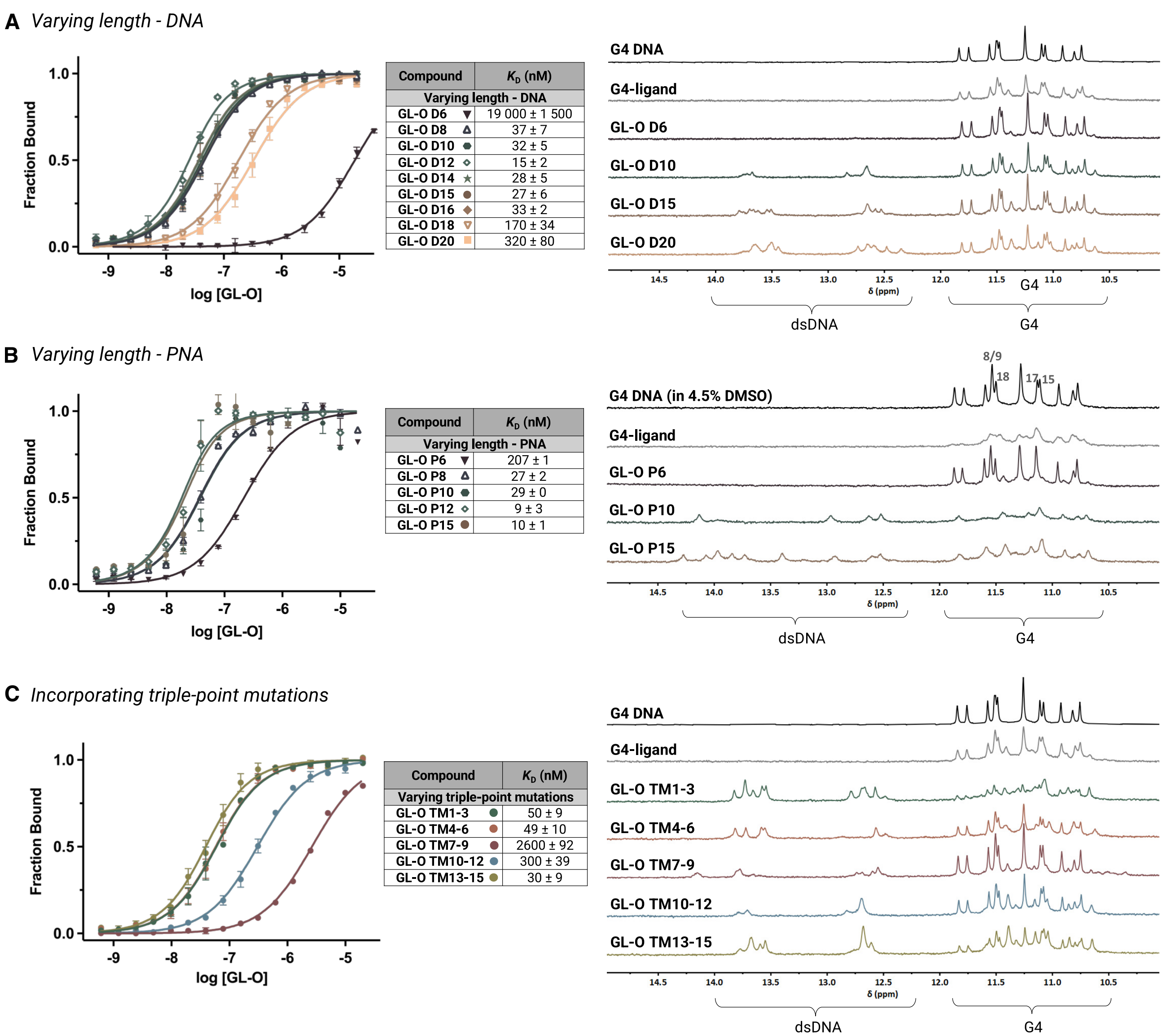


**Figure 3. Binding properties of the modified GL-Os with the *c-MYC* Pu24T G4 DNA structure**

GL-Os were serially diluted and incubated with 5′-fluorescently labeled *c-MYC* Pu24T G4 DNA. $^1$H-NMR profiles of the G4 DNA in 1:1 molar ratios with *c-MYC* Pu24T with complementary flanking sequence (90 μM). Error bars correspond to two independent measurements. DNA-based constructs were analyzed in aqueous buffer, whereas PNA-based constructs required the presence of 4.5% (v/v) DMSO for NMR measurements to ensure solubility; DMSO had no effect on MST-derived binding parameters. G4 imino proton signals appear between 10 and 12 ppm and double-stranded DNA imino proton signals between 12 and 14 ppm. (A) Varying the length of the DNA oligonucleotide (**GL-Os D6–D20**). (B) Varying the length of the PNA oligonucleotide (**P6–P15**). (C) Incorporating triple-point mutations in the oligonucleotide sequence (**GL-Os TM1–3**, **TM4–6**, **TM7–9**, **TM10–12**, and **TM13–15**).

the binding affinity where **GL-O P15** had a significantly better affinity than **GL-O P10**.

### *Effect of sequence complementarity*

For the triple-point mutation GL-O series (**GL-Os TM1–3** to **TM13–15**), MST results revealed a clear mismatch position dependence. Mutations positioned closer to the center of the oligonucleotide sequence (**GL-O TM7–9**) resulted in higher $K_D$ values, indicating weaker binding, whereas mutations near the terminal regions (**GL-Os TM1–3** and **TM13–15**) had only minor effects (Figure 3C). NMR analysis supported these observations (Figure 3C). All mutation variants produced detectable dsDNA signals, but the degree of G4 interaction varied. Constructs with terminal mismatches exhibited extensive broadening and downfield shifts of the G4 imino

resonances, indicating that the ligand remained engaged with the G4. In contrast, central mismatches (**GL-O TM7–9**) led to diminished broadening and weaker G4 signal intensity, consistent with reduced ligand binding when hybridization in the core region is disrupted. Interestingly, mismatches adjacent to the G4 (**GL-O TM1–3**) appeared to enhance the ligand's effect on the G4 structure, possibly by introducing local flexibility while maintaining the spatial configuration necessary for optimal G4 interaction.

### G4 stabilization of the oligonucleotide-modified GL-Os

The GL-O strategy has previously been shown to stabilize G4 DNA structures, as demonstrated by thermal stability assays and increased stalling with Taq DNA polymerase stop assays.[10,11] Consistent with its binding mechanism, oligonucleotide hybridization and G4 ligand interaction are expected to act synergistically to stabilize the G4 conformation. To assess this, thermal stability was first evaluated using $^{1}$H-NMR by gradually increasing the temperature from 25°C to 35°C and then 45°C, followed by 2.5°C increments up to 65°C (Figure 4A). The GL-Os in complex with the G4 DNA at a 1:1 molar ratio were compared to the G4 DNA and G4 ligand alone with and without DMSO (Figure S31). To determine whether thermal stabilization of the G4 structure results in a functional consequence, a Taq polymerase stop assay was performed (Figure 4B). The assay, previously described,[10,11] evaluates DNA polymerase progression on a G4-containing DNA template in the presence or absence of GL-Os. While Taq DNA polymerase exhibits baseline stalling at the G4 site, increased stalling, reflected by reduced full-length (FL) DNA product, indicates enhanced G4 stabilization that impedes polymerase G4 bypass.

#### *Effect of oligonucleotide length*

Among the DNA-based GL-Os tested (**D10**, **D15**, and **D20**), increasing oligonucleotide length correlated with elevated melting temperatures ($T_m$) for both the duplex DNA and the G4 ligand dissociation from the G4 DNA structure (Figure 4A). The shortest construct, **GL-O D6**, failed to hybridize with the G4 flanking sequence and showed only minor G4-ligand-induced effects, which was rapidly lost upon increasing the temperature (Figure S32). In contrast, the longest construct, **GL-O D20**, exhibited both the highest duplex $T_m$ and the highest G4 ligand dissociation temperature (about 65°C), showing that longer oligonucleotide guides can form highly thermally stable complexes once hybridized. Interestingly, for all tested DNA GL-Os, the melting of the duplex occurred at approximately the same temperature as the loss of G4/G4-ligand interaction, indicating a coordinated stabilizing effect (Figure 4A). When compared with the corresponding unconjugated oligonucleotides, it is evident that conjugation of the G4 ligand in the GL-Os substantially increases duplex stability, clearly illustrated by the enhanced melting profile of **GL-O D20** relative to its unconjugated counterpart **oligo D20** (Figure 4A).

Consistent with these thermal stabilization trends (Figure 4A), all DNA GL-Os, except the shortest construct **GL-O D6**, showed a dose-dependent increase on polymerase stalling, indicative of enhanced G4 stabilization, (Figures 4B and S33). Although this assay does not resolve fine differences between oligonucleotide lengths, it reveals a general trend in which longer oligonucleotides, such as in **GL-Os D16** and **D18**, induce stronger polymerase stalling, resulting in reduced formation of the FL product (Figure 4B). This correlates well with the high thermal stabilization induced by **GL-O D20** in the NMR melting experiments.

#### *Effect of backbone composition*

Overall, the PNA GL-Os displayed higher thermal stabilization than the DNA-based GL-Os toward the G4 target with flanking sequence. This was evident from the preservation of dsDNA peaks and G4 interaction peaks at higher measured temperatures (Figure 4A). Notably, **GL-O P15** retained strong duplex and G4-ligand-related signals even at the highest temperature measured (65°C), whereas these signals disappeared at approximately 55°C for **GL-O D15**. However, unlike the DNA GL-Os, the unconjugated PNA oligonucleotides exhibited similar duplex melting profiles to their conjugated counterparts (Figure 4A). This reflects the inherently stronger hybridization of PNA, which produces highly potent GL-Os and also reduces their dependence on G4 ligand binding. This is a factor that should be considered in terms of potential nonspecific interactions. Furthermore, the melting behavior of the PNA-based GL-Os differed from that of the DNA series and more closely resembled the profile of the free G4 ligand (Figure S31), likely reflecting the large difference in physicochemical properties between the PNA and DNA backbones. Specifically, the PNA-based GL-Os retained broader G4 imino proton signal patterns throughout the melting experiments, consistent with persistent G4 ligand interactions at elevated temperatures. This was particularly evident for **GL-O P10** relative to **GL-O D10**, where the G4 peak profile more closely resembled that observed for the free G4 ligand bound to the G4 structure.

Consistent with the NMR data, in the Taq polymerase stop assay, constructs containing longer PNA oligonucleotides exhibited more pronounced inhibition of polymerase progression, consistent with stronger G4 stabilization (Figures 4B and S34). The dose-response profiles for the PNA-based GL-Os displayed a steep transition, with a narrow range between concentrations showing no effect and those producing complete inhibition at very low concentrations. Both **GL-Os P12** and **P15** were inactive at 0.04 μM but induced a complete loss of the FL product at 0.2 μM (Figure 4B).

To further probe the strong affinity of PNA relative to DNA, we examined the corresponding unconjugated PNA oligonucleotides. In contrast to the DNA oligos, the PNA oligos strongly inhibited polymerase extension, but this effect did not originate from G4 stabilization. Instead, the data suggest that polymerase progression is blocked when encountering a PNA:DNA hybrid downstream of the G4. This was evident from **oligo P15**, which showed a strong oligo stalling band (Figure 4C).

Importantly, this artifact was not observed for the PNA-based GL-Os. Upon conjugation of the G4 ligand, the GL-Os showed inhibition

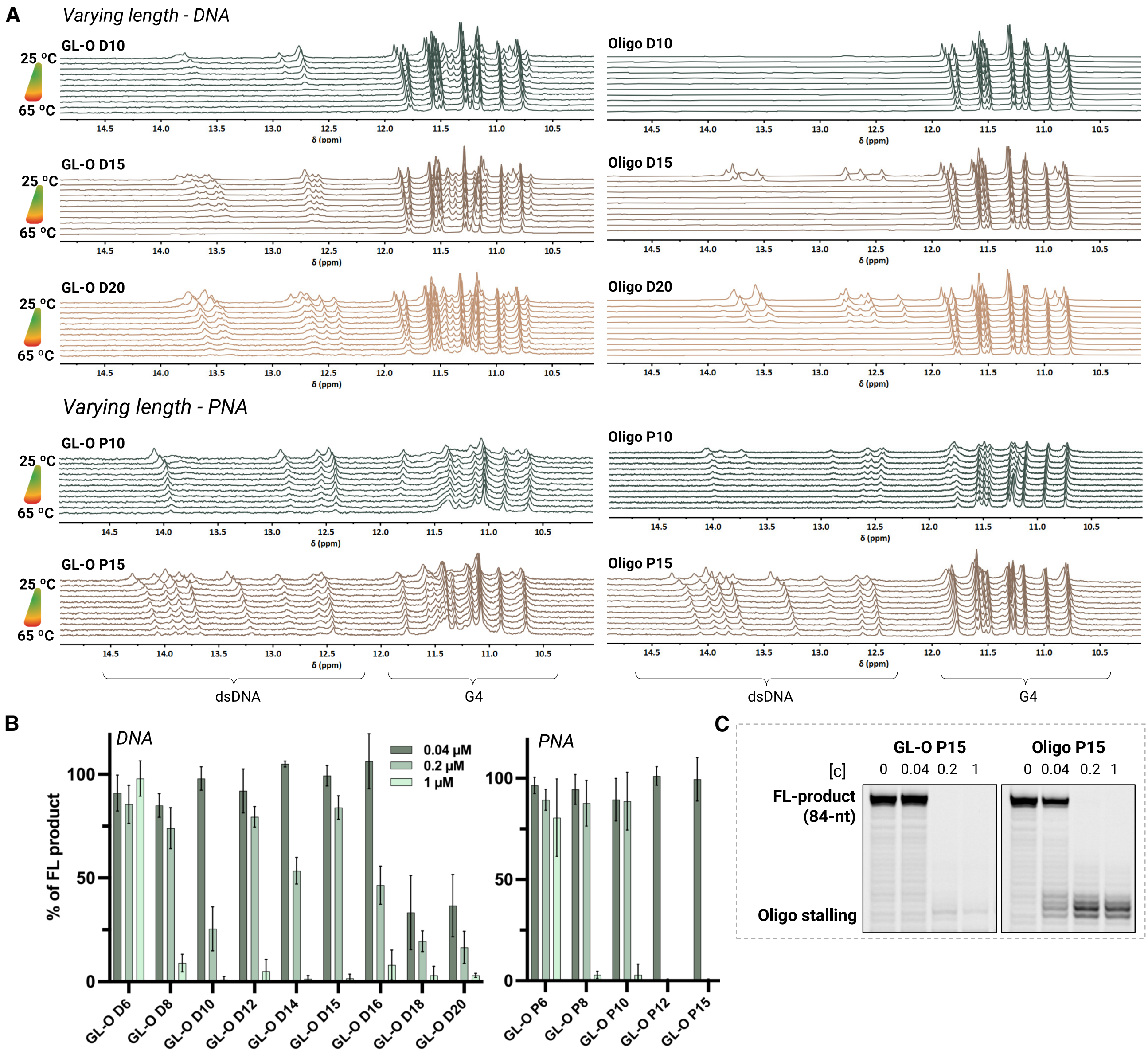


**Figure 4. G4 stabilization of the *c-MYC* Pu24T G4 DNA structure by the modified GL-Os**

(A) Comparison between unconjugated oligonucleotides and with G4 ligand using $^1$H-NMR (GL-Os and oligos **D10**, **D15**, and **D20** and GL-Os and oligos **P10** and **P15)**. Spectra were recorded of *c-MYC* Pu24T with complementary 5′-flanking sequence at 25°C and 35°C and a 2.5°C temperature ramp from 45°C to 65°C. Spectra for DNA-based constructs were recorded in aqueous buffer, whereas PNA-based constructs required the presence of 4.5% (v/v) DMSO to ensure solubility. G4 imino signals appear between 10 and 12 ppm, and double-stranded DNA signals appear between 12 and 14 ppm. (B) Taq polymerase stop assay that measures stabilization of the G4 DNA in the presence of the modified **GL-Os D6–D20** and **P6–P15**. Quantification of the Taq polymerase stop assay, shown as the full-length (FL, 84 nt) DNA product, is expressed as a percentage of the FL band intensity that was observed in the control reaction containing only the G4 template. Data represent the mean ± standard deviation from three independent experiments. (C) Gels of **GL-O P15** and **oligo P15** showing the FL(84 nt) DNA product band and oligo stalling band.

exclusively attributable to G4 stabilization, with no evidence of PNA-induced polymerase stalling (Figures 4B and S34). This indicates that the strong affinity of PNA is translated into G4 stabilization only when conjugated to the G4 ligand, an effect that was particularly pronounced for the longer 12- and 15-nt GL-Os. This conclusion was further supported by circular dichroism measurements, in which melting temperature profiles of G4 DNA alone were compared with those obtained in the presence of the G4 ligand, unconjugated PNA oligonucleotides (**P6**, **P10**, and **P15**), or the corresponding GL-Os (**P6**, **P10**, and **P15**) (Figure S35). While the PNA oligonucleotides alone did not stabilize the G4 DNA, ligand-conjugated PNA GL-Os produced substantial G4 stabilization, exceeding that of the G4

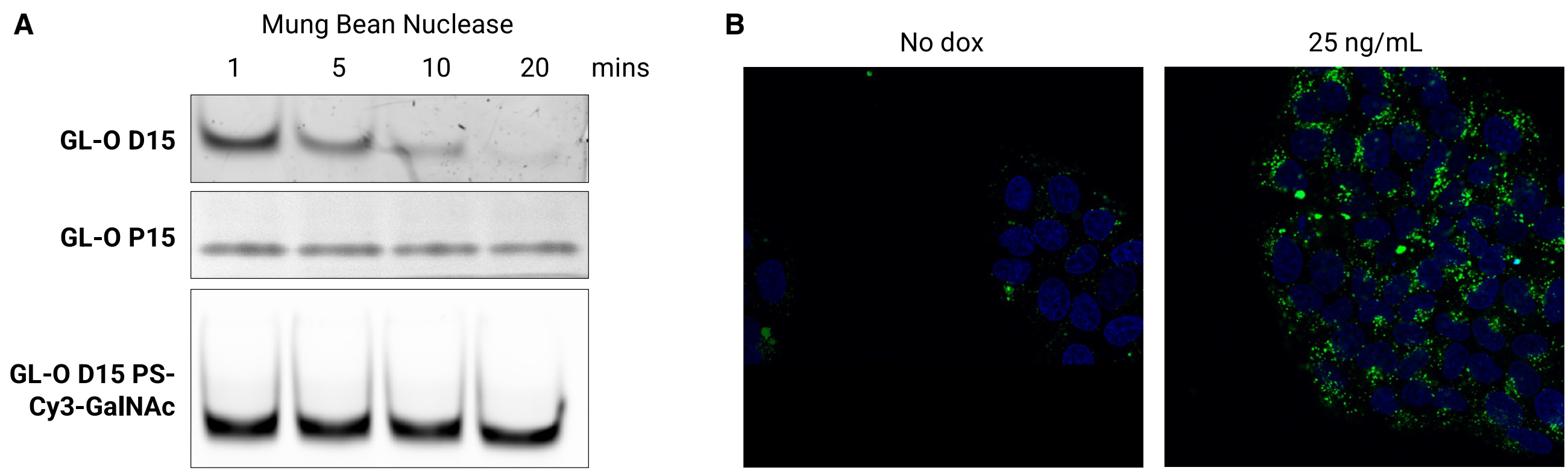


**Figure 5. Biological evaluation of GL-Os**

(A) *In vitro* digestion of GL-Os by mung bean nuclease. GL-Os were incubated with mung bean nuclease at 30°C and quenched at the indicated time points. Digestion products were analyzed by PAGE-DNA on 16% TBE gels stained with GelRed and PNA on 4%–20% SDS-PAGE gels stained with InstantBlue. Gels were imaged using a ChemiDoc (Bio-Rad) system. (B) Confocal microscopy images of **GL-O D15-PS-Cy3-GalNAc** in HEK293 cells with ASGR1 receptor-mediated uptake.

ligand alone, particularly for **GL-O P10** and **GL-O P15**. This further supports that the GL-O design preserves G4-specific activity while mitigating the nonspecific hybridization-driven interference observed for unconjugated PNA oligonucleotides.

### *Effect of sequence complementarity*

GL-Os containing central triple-point mutations (**GL-Os TM4–6**, **TM7–9** and **TM-10–12**), which exhibited reduced binding affinity, also failed to induce thermal stabilization effects (Figure S36). These constructs showed minimal or no interaction with the G4 even at 35°C. In contrast, GL-Os with terminal mutations retained G4 association up to 50°C–55°C, consistent with the higher tolerance for mismatches near the duplex ends observed in the binding assays (Figure 3A). For the unconjugated oligonucleotides, no G4 stabilization was observed (Figure S37).

Consistent with these observations, in the Taq polymerase stop assay, no significant differences in the FL product were observed at 0.04 μM as all triple-mutant GLOs displayed minimal effects at this concentration. However, at increasing concentrations, a clear trend emerged where GL-Os with central mutations (**TM7–9** and **TM10–12**) exhibited the weakest G4 stabilization, yielding approximately 50% FL product at the highest concentration tested (Figure S37), consistent with the NMR melting data.

### Stability of GL-Os toward endonuclease

GL-Os exhibit strong affinity and robust G4 stabilization *in vitro*, but their suitability for cellular applications also depends on nuclease resistance. To assess this, we incubated DNA- and PNA-based GL-Os with the length of 15 nt (Figure 5A), together with their unconjugated oligonucleotide as controls (Figure S38), in the presence of internally cleaving nuclease.

Time-dependent degradation of DNA-based GL-O was observed upon incubation with a restriction endonuclease mung bean nuclease (MBN), as evidenced by progressive loss of FL DNA oligonucleotides (unconjugated and conjugated) on TBE polyacrylamide gel electrophoresis (PAGE). Interestingly, the conjugated **GL-O D15** showed to be more metabolically stable than its unconjugated **oligo D15** (Figure 5A; Figure S38).

In contrast, PNA-based GL-O and unconjugated oligonucleotide remained intact over the same incubation period, and no detectable degradation products are observed on SDS-PAGE, indicating resistance to MBN digestion (Figure 5A; Figure S38), highlighting that PNA improves the metabolic stability of the GL-O, a feature required for cellular activity.

### Cell entry and localization of DNA-based GL-Os

After assessing the GL-O metabolic stability, we next investigated whether the GL-O strategy could be adapted for receptor-mediated cellular delivery and intracellular tracking. We re-designed the GL-O D15 to incorporate a full phosphorothioate (PS) backbone, a 3′-Cy3 fluorophore labeling, and a 3′-trivalent GalNAc moiety (**GL-O D15-PS-Cy3-GalNAc**) to enable ASGR1 receptor-mediated uptake. The PS-Cy3-GalNAc-modified GL-O and unconjugated oligonucleotide were subsequently evaluated for nuclease stability and remained stable for at least 20 min, comparable to that of the PNA-based GL-O (Figure 5A; Figure S38).

To enable controlled evaluation of GalNAc-mediated uptake, we generated an inducible ASGR1-expressing HEK293 cell line (Figure S2). We next examined receptor-mediated uptake of **GL-O D15-PS-Cy3-GalNac** in ASGR1-expressing HEK293 cells. In the absence of ASGR1 expression, only minimal GL-O uptake was observed. In contrast, receptor expression led to a several fold increase in uptake efficiency of the fluorescent oligo, demonstrating efficient receptor-mediated internalization. Notably, the fluorescent signal was predominantly localized to the cytoplasm with a punctate staining pattern, consistent with localization within endosomal

compartments following receptor-mediated uptake. The limited nuclear signal highlights endosomal escape as the principal remaining step to unlock the full potential of this delivery strategy (Figure 5B).

## DISCUSSION

This study provides a systematic analysis of the molecular determinants governing G4 recognition and stabilization by GL-Os. By varying oligonucleotide length, backbone composition, and sequence complementarity, we identify the key parameters that control GL-O binding affinity, structural engagement, and functional G4 stabilization.

A central component of this work was the successful synthesis and characterization of a comprehensive GL-O library, comprising twenty conjugates. The use of complementary conjugation strategies, including a robust copper-free click reaction for DNA-based GL-Os and amide coupling for PNA-based constructs, enabled the modular construction of structurally diverse GL-Os. This dual synthetic approach allowed incorporation of chemically distinct oligonucleotide backbones without altering the G4 ligand scaffold, allowing systematic investigation of how oligonucleotide length, backbone composition, and sequence complementarity influence G4 targeting and stabilization.

Genome-wide alignment analyses across three representative G4 targets (*c-MYC*, *KRAS*, and *Helicase B*) established an important conceptual framework for GL-O selectivity. These analyses revealed that sequence uniqueness is highly target dependent, with the *c-MYC* Pu27 region retaining single-locus specificity at oligonucleotide lengths as short as 14 nt, whereas other loci require longer sequences to achieve comparable discrimination. Although shorter oligonucleotides showed partial complementarity to additional genomic loci, such matches are unlikely to result in functional off-target effects. The GL-O strategy relies on a dual-binding requirement, whereby productive engagement necessitates both oligonucleotide hybridization to a flanking single-stranded region and simultaneous binding of the G4 ligand to a neighboring G4 structure.[10–12] This interdependence implies that the oligonucleotide does not need to achieve full genome-wide uniqueness, as functional binding will occur only where sequence complementarity coincides with a compatible G4 motif. Thus, the dual-recognition mechanism provides an inherent safeguard against nonspecific interactions while allowing flexibility in oligonucleotide design.

Experimental characterization further demonstrates that oligonucleotide length exerts a dual effect on GL-O performance. Short guide strands (<8 nt) fail to hybridize effectively and show minimal G4 interaction, while intermediate lengths (10–16 nt) bind efficiently but yield moderate stabilization. In contrast, longer oligonucleotides ($\geq$18 nt) show reduced apparent binding in the initial MST measurements, likely reflecting slower hybridization under the assay conditions, yet drive markedly higher G4 stabilization, as measured by both thermal denaturation and polymerase stop assays. This indicates that although longer guides may associate more slowly, they form more persistent and thermally stable complexes once bound. The stabilization data for the DNA GL-Os further reveal a strong interdependence between duplex formation and G4 ligand interaction, demonstrating that both elements act cooperatively to reinforce each other. This dual engagement not only enhances overall complex stability but also anchors the guide oligonucleotide at the correct genomic site, thereby minimizing the likelihood of off-target G4 binding.

Backbone composition further modulates these effects. PNA-based GL-Os exhibited greater thermal stability and prolonged G4 association relative to their DNA counterparts, consistent with the high affinity of the neutral PNA backbone.[15] Importantly, G4 ligand conjugation transforms PNA guides from nonspecific polymerase-blocking agents into selective G4 stabilizers, thereby preserving G4-specific activity. While complementary genomic sequences lacking adjacent G4 structures could in principle contribute to off-target interactions for PNA-based GL-Os, productive interactions at such loci would still require local accessibility of the target sequence within genomic DNA beyond simple sequence complementarity. Productive interactions are therefore expected to preferentially occur at regions associated with G4 formation or related local DNA structural dynamics. Importantly, PNA GL-Os also exhibit enhanced metabolic stability, a critical prerequisite for sustaining subsequent cellular activity. This property underscores the advantageous nature of PNA GL-Os within the GL-O platform.

Sequence complementarity emerged as another critical determinant of GL-O function. Central mismatches within the oligonucleotide severely compromised binding affinity and G4 stabilization, whereas terminal mismatches were better tolerated. This behavior is consistent with general thermodynamic principles of nucleic acid pairing, where internal mismatches impose the greatest destabilizing penalties,[17] and demonstrates that central complementarity is essential not only for duplex formation but also for maintaining effective G4 ligand engagement.

Our successful receptor-mediated internalization of GL-Os into cells represents an important step toward enabling functional intracellular studies of this modality. The PS- and GalNAc-modified GL-O architecture established here demonstrates that GL-Os can be adapted for targeted cellular uptake while maintaining metabolic stability. Importantly, the predominantly punctate cytoplasmic localization observed for the fluorescent GL-Os indicates that endosomal escape and efficient nuclear trafficking remain key challenges for achieving functional intracellular activity at genomic G4 targets. These findings therefore establish both a delivery-compatible GL-O design and identify intracellular trafficking as a critical next step for future development of the strategy. PNA-based GL-Os are likely to pose additional delivery challenges due to their reduced solubility and neutral backbone properties and may therefore require further optimization strategies, such as conjugation to cell-penetrating peptides or alternative delivery systems,[18] to achieve efficient intracellular and nuclear delivery.

In conclusion, these findings show that effective GL-O binding and G4 stabilization depend on a balance between oligonucleotide hybridization and G4 ligand engagement. Oligonucleotide length, backbone composition, and sequence complementarity jointly determine this balance: short sequences hybridize inefficiently, longer sequences hybridize more slowly but form more stable complexes, and central mismatches destabilize duplex formation, while PNA substitution enhances binding strength but requires ligand conjugation to maintain specificity. This interdependence stabilizes the oligonucleotide at the target site and limits off-target interactions. Together with the demonstrated receptor-mediated cellular uptake of modified GL-Os, these results underscore the pivotal role of the oligonucleotide component in determining GL-O behavior and highlight the modular, chemically adaptable nature of the strategy. These findings provide a foundation for the future rational design of GL-Os for probing G4 biology and for future studies aimed at evaluating their therapeutic potential, particularly in oncogenic contexts such as the *c-MYC* promoter.

## MATERIALS AND METHODS

### Conjugation of G4 ligand with DNA oligonucleotides

5′-Hexylamine oligonucleotide (50 nmol) was dissolved in 0.1 M $NaHCO_3$ (50 μL). BCN-NHS ester (20 equiv., 0.2 M in DMSO) was added, and the mixture was shaken for 24 h. The product was precipitated twice using 3 M NaOAc and 4× volume ethanol, washed with ethanol, dried under $N_2$, and redissolved in 0.1 M $NaHCO_3$. An azide-functionalized G4 ligand (2.5 equiv., 10 mM in DMSO) was added, and the reaction was shaken for 24 h. The mixture was diluted, filtered, and purified by reverse-phase HPLC (C18 column, 2 mL/min, 5%–60% ACN in 50 mM TEAA buffer). For chromatograms of purified GL-Os, see Figures S3–S11 and S17–S22. Conjugates were confirmed by high resolution mass spectrometry (HRMS) ($ESI^-$).

### PNA synthesis

PNA synthesis was done on an automated solid phase peptide synthesizer (SPPS) on a 20 μmol scale using Fmoc-Lys(Boc)-Wang (0.61 mmol/g loading) resin for **P6**–**P10** and Fmoc-Lys(Boc)-Wang (0.2–0.4 mmol/g) for **P12**–**P15** (more details in Figure S1). The resin was allowed to swell in dichloromethane (DCM) for 30 min. The Fmoc protecting group was removed by treating the resin with 20% (v/v) piperidine in N,N-dimethylformamide (DMF). Fmoc-PNA(Bhoc)-OH monomers (0.50 M in DMF) were coupled using HATU (0.49 M in N-methyl-2-pyrrolidone [NMP]) and DIPEA (2.0 M in DMF) for 60 min. A capping step was performed after each coupling using 50% (v/v) $Ac_2O$ in DCM for 40 min. Cleavage and deprotection were performed using a mixture of TFA/m-cresol (4:1) for 1 h at room temperature. The cleavage mixture was filtered into ice-cold diethyl ether, and the resulting precipitate was diluted, filtered, and subjected to purification by RP-HPLC (C18 column, 2 mL/min, 0%–30%, and then 30%–100% solvent B [100% ACN] and solvent A [0.1% formic acid]). For chromatograms of purified GL-Os, see Figures S12–S16. The conjugated products were confirmed by HRMS ($ESI^+$).

### Conjugation of G4 ligand with PNA oligonucleotides

The G4 ligand (0.25 M, 2.5 equiv.) was solubilized in NMP and added to the resin (5 μmol) along with HATU (0.24 M in NMP) and DIPEA (1.0 M in DMF). The reaction was allowed to shake for 24 h at room temperature. The conjugated PNAs were cleaved from the resin following the aforementioned procedures.

### MST

Cy5-labeled G4 DNA was annealed in MST buffer (10 mM potassium phosphate, 100 mM KCl, 0.05% Tween 20, pH 7.4) by heating at 96°C for 5 min and cooling to room temperature. MST measurements were performed on a Monolith NT.115 using standard capillaries, with G4 DNA at 20 nM and serially diluted GL-Os, unconjugated oligonucleotides, and G4 ligand (5–40 μM). DNA-based oligonucleotides were prepared in aqueous buffer. For PNA-based GL-Os and unconjugated PNA oligonucleotide controls, experiments were performed both in the presence and absence of DMSO, and no differences in thermophoresis traces or derived binding affinities were observed. $K_D$ were obtained using Monolith analysis software, and dose-response curves were plotted in GraphPad Prism 10.

### $^1$H-NMR spectroscopy

G4 DNA (110 μM) was annealed in 10 mM potassium phosphate buffer (3 mM KCl, pH 7.4) following same procedure as for MST. Before measuring, 10% $D_2O$ was added, and the sample (100 μM) was transferred to a 3 mm tube. GL-O, unconjugated oligonucleotide (1 mM), or G4 ligand (10 mM) were added in separate experiments to achieve a 1:1 molar ratio relative to G4 DNA, and $^1$H-NMR spectra were recorded after equilibration. DNA-based GL-Os were prepared in aqueous buffer, whereas PNA-based constructs required the addition of DMSO to a final concentration of 4.5% (v/v) to ensure complete solubility. Experiments were performed on an 850 MHz Bruker AVANCE III HD spectrometer at 298 K using excitation sculpting (512 scans). Thermal melting profiles were recorded at 308 K then a temperature ramp of 2.5 K from 318 to 338 K. Data were processed with MestreNova 10.0.2.

### Taq polymerase stop assay

DNA templates were annealed with a fluorescent primer in 100 mM KCl by heating to 95°C and cooling to room temperature. Reactions (40 nM template) were prepared in 1× Taq buffer (10 mM Tris-HCl pH 8.8, 50 mM KCl), 1.5 mM $MgCl_2$, 0.05 U/μL Taq polymerase, and compound as indicated. DNA-based oligonucleotides were prepared in water. For PNA-based GL-Os and unconjugated PNA oligonucleotide controls, assays were performed both in the presence and absence of DMSO, and no differences in polymerase stalling or FL product formation were observed. After 10 min on ice, dNTPs (100 μM) were added and samples were incubated at 37°C for 15 min. Reactions were stopped with 2× stop solution and separated on a 12% denaturing TBE gel. Fluorescence was detected using a Typhoon scanner, and band intensities were quantified with ImageQuant TL 10.2.

### *In vitro* endonuclease digestion

Stability of GL-Os and unconjugated oligonucleotides against mung bean nuclease digestion was assessed. 4 μM oligo was incubated with 3 units of enzyme in 1× MBN buffer at 30°. At indicated time points, reactions were quenched by adding DNA gel loading dye (50 mM EDTA, 50 mM HEPES, 30% glycerol, and 0.001% bromophenol blue) and heating at 95°C. Digestion products were analyzed by PAGE. DNA samples were resolved on 16% TBE gels for 1 h at 100 V in 1× TBE buffer. PNA samples were loaded on 4%–20% SDS-PAGE gels (Mini-PROTEAN TGX gels, Bio-Rad) and run for 1 h at 120 V in 1× SDS running buffer (Tris glycine). TBE gels were stained with gel red (Biotium), while SDS-PAGE gels were stained with InstantBlue (Abcam). Gels were imaged using a ChemiDoc Imaging system (Bio-Rad).

### CD melting temperature assay

G4 DNA (3 μM) was folded in K-phosphate buffer (10 mM, pH 7.4) containing KCl (3 mM) by heating for 5 min at 95°C, then allowed to cool to room temperature. PNA in DMSO was added in a 1:1 ratio, resulting in a final DMSO concentration of 0.3%. CD measurements were performed on a J-1700 circular dichroism spectrophotometer (Jasco International Co. Ltd.). A quartz cuvette with a path length of 1 mm was used for measurements. CD spectra were recorded between λ = 200–350 nm with an interval of 0.5 nm and a scan rate of 100 nm/min. Thermal melting was recorded between 25°C and 95°C with increments of 10°C at a speed of 1°C/min.

### Sequence alignment analysis

The specificity of 5′ G4 flanking sequences of varying lengths was assessed by aligning these DNA sequences to the human reference genome. The human reference genome (GRCh38) was retrieved from the UCSC Genome Browser,[19] and flanking sequence was obtained from NCBI nucleotide database. FASTA datasets containing flanking sequences of 5–20 bases were generated and each dataset was independently aligned to the human reference genome using Bowtie 2.0,[20] with no mismatch allowed. The aligned reads in SAM files were converted to BAM format and subsequently sorted and indexed using SAMtools[21] with default settings. The final BAM files were used for downstream sequence annotation and comparative analysis of mapping specificity across different flanking lengths.

### Cell line generation

To enable cellular uptake and tracking of GL-O internalization, a trivalent GalNAc moiety and a Cy3 fluorophore were incorporated at the 3′ end of an oligonucleotide, which was subsequently conjugated to the G4 ligand as described previously. Flp-In T-REx 293 cells (Thermo Fisher Scientific) were engineered for doxycycline-inducible expression of the asialoglycoprotein receptor 1 (ASGR1). The *ASGR1* coding sequence (CDS), flanked by restriction sites, was synthesized by Eurofins and subcloned into the pcDNA5/FRT/TO vector. The Flp-In system enables site-specific integration of the gene of interest via Flp recombinase-mediated recombination; its key features are summarized in Figure S2. Briefly, Flp-In T-REx 293 cells were co-transfected with pcDNA5/FRT/TO-ASGR1 (600 ng) and pOG44 (6 μg) using TurboFect (Thermo Fisher Scientific). Hygromycin-resistant isogenic colonies were pooled and expanded. ASGR1 expression following doxycycline induction (25 ng/mL) was confirmed by western blot using an anti-ASGR1 antibody (Proteintech; Figure S2).

### Microscopy

Uptake and intracellular localization of the GalNAc- and Cy3-modified GL-O were assessed by live-cell confocal microscopy. ASGR1-expressing HEK293 cells were seeded onto glass-bottom dishes and induced overnight with doxycycline (25 ng/mL). Cells were then incubated with 0.5 μM Cy3-conjugated GalNAc oligonucleotide for 24 h prior to imaging. Live-cell imaging was performed at the Biochemical Imaging Centre Umeå on a Leica SP8 FALCON confocal microscope equipped with a 63× oil-immersion objective.

## DATA AND CODE AVAILABILITY

Detailed synthetic procedures and conjugation methods and experimental data for the assays are provided in the ESI.

## ACKNOWLEDGMENTS

Work in the Chorell lab was supported by grants from the Swedish Research Council (VR-NT 2021–04805), the Swedish Cancer Foundation (23 2793 Pj), Lion's Cancer Research Foundation in Northern Sweden (LP 24–2352), and the Kempe Foundations (JCK-3159). We thank the Knut and Alice Wallenberg foundation program "NMR for Life" for NMR spectroscopy support. Work in the Wanrooij lab was supported by the Knut and Alice Wallenberg Foundation, Kempe Foundations (SMK21-0059), and the Swedish Research Council (VR-MH 2023–02160).

## AUTHOR CONTRIBUTIONS

E.C. and S.W. initiated and directed the project. A.A., S.K., S.W., E.C., S.V., A.B., K.A., N.C., C.K., and L.S. developed the project. A.A., E.C., and S.W. wrote the manuscript. C.K. performed the sequence analysis. Oligonucleotide synthesis was performed by T.B. and A.D. PNA and PNA GL-O synthesis was performed by S.K. and S.V. Bioconjugations and biophysical assays were performed by A.A., S.K., and L.S. Polymerase stop assays were performed by A.B., K.A., and L.S. *In vitro* digestion of GL-Os was performed by N.C. and A.A. Analysis of the data was done by all authors. All authors read and edited the manuscript.

## DECLARATION OF INTERESTS

The authors declare no conflict of interest.

## SUPPLEMENTAL INFORMATION

Supplemental information can be found online at https://doi.org/10.1016/j.omtn.2026.103061.

## REFERENCES

1. Dell'Oca, M.C., Quadri, R., Bernini, G.M., Menin, L., Grasso, L., Rondelli, D., Yazici, O., Sertic, S., Marini, F., Pellicioli, A., et al. (2024). Spotlight on G-Quadruplexes: From Structure and Modulation to Physiological and Pathological Roles. Int. J. Mol. Sci. *25*, 3162. https://doi.org/10.3390/ijms25063162.

2. Lane, A.N., Chaires, J.B., Gray, R.D., and Trent, J.O. (2008). Stability and kinetics of G-quadruplex structures. Nucleic Acids Res. *36*, 5482–5515. https://doi.org/10.1093/nar/gkn517.

3. Hänsel-Hertsch, R., Beraldi, D., Lensing, S.V., Marsico, G., Zyner, K., Parry, A., Di Antonio, M., Pike, J., Kimura, H., Narita, M., et al. (2016). G-quadruplex structures mark human regulatory chromatin. Nat. Genet. *48*, 1267–1272. https://doi.org/10.1038/ng.3662.

4. Lago, S., Nadai, M., Cernilogar, F.M., Kazerani, M., Domíniguez Moreno, H., Schotta, G., and Richter, S.N. (2021). Promoter G-quadruplexes and transcription factors cooperate to shape the cell type-specific transcriptome. Nat. Commun. *12*, 3885. https://doi.org/10.1038/s41467-021-24198-2.
5. Siddiqui-Jain, A., Grand, C.L., Bearss, D.J., and Hurley, L.H. (2002). Direct evidence for a G-quadruplex in a promoter region and its targeting with a small molecule to repress c-*MYC* transcription. Proc. Natl. Acad. Sci. *99*, 11593–11598. https://doi.org/10.1073/pnas.182256799.
6. Chaudhuri, R., Bhattacharya, S., Dash, J., and Bhattacharya, S. (2021). Recent Update on Targeting c-MYC G-Quadruplexes by Small Molecules for Anticancer Therapeutics. J. Med. Chem. *64*, 42–70. https://doi.org/10.1021/acs.jmedchem.0c01145.
7. Boddupally, P.V.L., Hahn, S., Beman, C., De, B., Brooks, T.A., Gokhale, V., and Hurley, L.H. (2012). Anticancer Activity and Cellular Repression of c-MYC by the G-Quadruplex-Stabilizing 11-Piperazinylquindoline Is Not Dependent on Direct Targeting of the G-Quadruplex in the c-MYC Promoter. J. Med. Chem. *55*, 6076–6086. https://doi.org/10.1021/jm300282c.
8. Nuccio, S.P., Cadoni, E., Nikoloudaki, R., Galli, S., Ler, A.-J., Sanchez-Cabanillas, C., Maher, T.E., Fan, E., Guneri, D., Flint, G., et al. (2025). Chemically modified CRISPR-Cas9 enables targeting of individual G-quadruplex and i-motif structures, revealing ligand-dependent transcriptional perturbation. Nat. Commun. *17*, 385. https://doi.org/10.1038/s41467-025-67074-z.
9. Chambers, V.S., Marsico, G., Boutell, J.M., Di Antonio, M., Smith, G.P., and Balasubramanian, S. (2015). High-throughput sequencing of DNA G-quadruplex structures in the human genome. Nat. Biotechnol. *33*, 877–881. https://doi.org/10.1038/nbt.3295.
10. Berner, A., Das, R.N., Bhuma, N., Golebiewska, J., Abrahamsson, A., Andréasson, M., Chaudhari, N., Doimo, M., Bose, P.P., Chand, K., et al. (2024). G4-Ligand-Conjugated Oligonucleotides Mediate Selective Binding and Stabilization of Individual G4 DNA Structures. J. Am. Chem. Soc. *146*, 6926–6935. https://doi.org/10.1021/jacs.3c14408.
11. Abrahamsson, A., Berner, A., Golebiewska-Pikula, J., Chaudhari, N., Keskitalo, E., Lindgren, C., Chmielewski, M.K., Wanrooij, S., and Chorell, E. (2025). Linker Design Principles for the Precision Targeting of Oncogenic G-Quadruplex DNA with G4-Ligand-Conjugated Oligonucleotides. Bioconjug. Chem. *36*, 724–736. https://doi.org/10.1021/acs.bioconjchem.5c00008.
12. Abrahamsson, A., Khwaja, S., Berner, A., Nath Das, R., Aasumets, K., Chaudhari, R., Wanrooij, S., and Chorell, E. (2026). Tuning Potency for Precision: The Role of the G4-Ligand in G4-Ligand–Conjugated Oligonucleotides Targeting Individual G-Quadruplex DNA Structures. In Revised for RSC Chemical Biology] *Department of Chemistry and Department of Medical Biochemistry and Biophysics* (Umea University).
13. Sun, D., and Hurley, L.H. (2009). The Importance of Negative Superhelicity in Inducing the Formation of G-Quadruplex and i-Motif Structures in the c-Myc Promoter: Implications for Drug Targeting and Control of Gene Expression. J. Med. Chem. *52*, 2863–2874. https://doi.org/10.1021/jm900055s.
14. Egholm, M., Buchardt, O., Nielsen, P.E., and Berg, R.H. (1992). Peptide nucleic acids (PNA). Oligonucleotide analogs with an achiral peptide backbone. J. Am. Chem. Soc. *114*, 1895–1897. https://doi.org/10.1021/ja00031a062.
15. Wan, W.B., and Seth, P.P. (2016). The Medicinal Chemistry of Therapeutic Oligonucleotides. J. Med. Chem. *59*, 9645–9667. https://doi.org/10.1021/acs.jmedchem.6b00551.
16. Nielsen, P.E. (2002). Peptide Nucleic Acids - Methods and Protocols (Humana Press Inc.).
17. Piao, X., Sun, L., Zhang, T., Gan, Y., and Guan, Y. (2008). Effects of mismatches and insertions on discrimination accuracy of nucleic acid probes. Acta Biochim. Pol. *55*, 713–720.
18. De, A., Kumar, V., Saltzman, W.M., Slack, F.J., Vikram, A., Gupta, A., and Bahal, R. (2026). Advances in peptide nucleic acid for targeting RNA and genomic DNA. Cell Rep. Phys. Sci. *7*, 103061. https://doi.org/10.1016/j.xcrp.2025.103061.
19. Casper, J., Speir, M.L., Raney, B.J., Perez, G., Nassar, L.R., Lee, C.M., Hinrichs, A.S., Gonzalez, J.N., Fischer, C., Diekhans, M., et al. (2026). The UCSC Genome Browser database: 2026 update. Nucleic Acids Res. *54*, D1331–D1335. https://doi.org/10.1093/nar/gkaf1250.
20. Langmead, B., and Salzberg, S.L. (2012). Fast gapped-read alignment with Bowtie 2. Nat. Methods *9*, 357–359. https://doi.org/10.1038/nmeth.1923.
21. Li, H., Handsaker, B., Wysoker, A., Fennell, T., Ruan, J., Homer, N., Marth, G., Abecasis, G., and Durbin, R.; 1000 Genome Project Data Processing Subgroup (2009). The Sequence Alignment/Map format and SAMtools. Bioinformatics *25*, 2078–2079. https://doi.org/10.1093/bioinformatics/btp352.

**OMTN, Volume *37***

## Supplemental information

# Dual recognition drives site-directed G-quadruplex stabilization: Oligonucleotide design in G4 ligand-oligonucleotide conjugates

**Alva Abrahamsson, Sakina Khwaja, Namrata Chaudhari, Steven Vertueux, Andreas Berner, Koit Aasumets, Chandan Kumar, Lotte Stietz, Tom Baladi, Anders Dahlén, Sjoerd Wanrooij, and Erik Chorell**

***Oligonucleotide conjugations***

**DNA Oligonucleotide conjugations**

All oligonucleotides received through a collaboration with the Oligonucleotides and Targeted Delivery group, Discovery Sciences at AstraZeneca R&D (Gothenburg, Sweden) or purchased from IDT (Integrated DNA Technologies, Sweden). 5´-hexylamine oligonucleotide ($Na^+$ salt, 50 nmol) was dissolved in 0.1 M $NaHCO_3$ (aq) (50 µL, 1 mM). A solution of (1R,8S,9s)-bicyclo[6.1.0]non-4-yn-9-ylmethyl N-succinimidyl carbonate (BCN-NHS ester) (20 equiv., 5 µL, 0.2 M) was freshly prepared and added to the oligonucleotide solution. The reaction solution was vortexed and shaken for 24 h. Followed by precipitation of oligonucleotide conjugate using 10% v/v of 3 M NaOAc and 4 times the volume EtOH and kept in freezer for 30 min. Then the solution was centrifuged for 15 min followed by removing the supernatant. The precipitate pellet was resuspended in the same volume of NaOAc and EtOH and repeated the precipitation. After this, the pellet was washed with only EtOH followed by drying the pellet by gently adding $N_2$ gas and then resuspended in 0.1 M $NaHCO_3$ (aq) (50 µL, 1 mM). The G4-ligand baring an azide functionality (previously described)[1] (2.5 equiv., 25 µL, 10 mM in DMSO), was added to the dissolved oligonucleotide and vortexed and shaken for 24 h. The reaction mixture was dissolved in more water and DMSO and filtered before purified by RP-HPLC. Purification was done using a C18 semi-preparative column and 2 mL/min flowrate and a linear gradient of 5 to 60% solvent B (100% ACN). Solvent A was a 50 mM triethylammonium acetate buffer. The conjugation products were confirmed with HRMS ($ESI^-$).

**Peptide Nucleic Acid synthesis and conjugations**

All reagents and solvents including resins, piperidine, hexafluorophosphate azabenzotriazole tetramethyl uronium (HATU), *N*,*N*-Diisopropylethylamine (DIPEA), trifluoro acetic acid (TFA), m-cresol, *N*-Methyl-2-pyrrolidone (NMP), acetonitrile (ACN), formic acid, diethyl ether was purchased from *Merck Sigma Aldrich*. The Fmoc-PNA(Bhoc)-OH monomers were purchased from *Iris Biotech GmbH*, Fmoc-6-Ahx-OH was purchased from *Chemtronica,* Peptide Grade dimethylformamide (DMF) was purchased from *CARLO ERBA Reagents*. The PP-reactors were purchased from *Biotage*. RP-HPLC was carried out on a Hitachi HPLC system using a Phenomenex Luna® Omega 5 µm Polar C18 100Å (250x 10 mm) column.

Peptide grade DMF was used for PNA synthesis. The reported equivalents are relative to the amount of resin, and all reactions were performed at room temperature.

The PNAs were synthesized on a 5-20 µmol scale on a Syro I automated peptide synthesizer (Biotage) using preloaded resin Fmoc-Lys(Boc)-Wang resin (loading 0.61 mmol/g) for **P6-P10** and Fmoc-Lys(Boc)-Wang LL (loading 0.2 – 0.4 mmol/g) for **P12-P15**. The PNA synthesis was performed in PP reactors with PE frit, and deprotection and cleavage were performed in PP reactors with PTFE frit.

The resin was allowed to swell in DCM for 30 min under agitation. Fmoc deprotection cycle was performed with 20% (v/v) piperidine in DMF for 15 min. The Fmoc-PNA(Bhoc)-OH monomers (0.5 M in DMF, 5 eq) were coupled for 1 h using HATU (0.5M in NMP, 5 eq), DIPEA (2.0 M in DMF) for 60 minutes. A capping step was performed after each coupling using $Ac_2O$ 50% (v/v) in DCM. After PNA elongation, the N-terminal was coupled with Fmoc-6-Ahx-OH, followed by Fmoc deprotection according to the aforementioned procedures. Conjugation to the G4-ligand was performed on a 5 µmol scale. The N-terminal amine of the monomers was coupled with G4-ligand (0.25 M in NMP, 2.5 eq), HATU (0.25 M in NMP, 5 eq), DIPEA (1.0 M in DMF), and the coupling time was extended to 24 h (Figure S16).

After the synthesis was completed, the resin was suspended in a mixture of TFA:m-cresol (4:1) and vortexed for 1.5 h, resulting in simultaneous deprotection and cleavage from the resin. The solution was filtered into ice-cold $Et_2O$ and centrifuged. The white precipitate was purified using RP-HPLC. Purification was carried out at room temperature using detection at 260 nm on a C18 semi-preparative column with a 2.5 mL/min flow rate in a gradient system of 0-20% B over 24 min then 20-100% B over 2 min (B 100% ACN and (A 0.1 Formic acid in Milli-Q water) for **P6-P10** and gradient system of 0-30%

over 11 min B then 30-100% B over 15 min (B 0.1% TFA in ACN and A 0.1% TFA in Milli-Q water). The conjugated products were confirmed with HRMS ($ESI^+$).

### *In vitro assays*

**Microscale thermophoresis (MST).** The G4 template consisting of the G4-forming sequence and the flanking sequence was purchased with a Cy5 5´-label. The G4 DNA was annealed in MST buffer (10 mM potassium phosphate, 100 mM KCl, 0.05% Tween 20, pH 7.4) by heating at 96 °C for 5 min followed by cooling down to room temperature before storing in the fridge overnight. All MST experiments were done on a Monolith NT.115 (Nanotemper, Germany) instrument and performed in MST buffer with standard Monolith capillaries. The G4 DNA concentration was kept constant at 20 nM, and the GL-Os were serial diluted (1:1) with highest concentration of 5-40 µM. For PNA-based GL-Os, binding experiments were conducted both in the presence and absence of DMSO, and no differences in thermophoresis traces or derived binding affinities were observed. MST traces and binding affinity constants ($K_D$) were obtained using the Monolith analysis software and plotted and visualized in GraphPad Prism 10.

**Proton nuclear magnetic resonance spectroscopy ($^1$H NMR)**. G4 DNA was prepared by annealing in 10 mM potassium phosphate buffer (3 mM KCl, 110 µM G4 DNA, pH 7.4). The solution was heated to 96 °C for 5 minutes, then gradually cooled to room temperature and stored overnight at 4 °C. Prior to NMR analysis, 10% $D_2O$ was added to the G4 DNA solution to achieve a final concentration of 100 µM, and the sample was transferred to a 3 mm NMR tube. GL-O (1 mM) was added to the DNA solution in two steps: first, 2 µL (0.5 equivalents) was added, and after 10 minutes, a $^1$H NMR spectrum was recorded. A second aliquot (0.5 equivalents) was then added to achieve a 1:1 molar ratio with G4 DNA, and another spectrum was recorded. For DNA-based GL-Os, samples were prepared in aqueous buffer, whereas PNA-based constructs required the addition of DMSO to a final concentration of 4.5% (v/v) to ensure complete solubility. NMR experiments were conducted on an 850 MHz Bruker AVANCE III HD spectrometer equipped with a 5 mm TCI cryoprobe at 298 K. The transmitter frequency offset (O1P) was set at 4.7 ppm, with a spectral width of 22 ppm. One-dimensional $^1$H NMR spectra were acquired using excitation sculpting with 512 scans per spectrum. For thermal stability studies, samples were equilibrated at temperatures ranging from 313 K to 338 K in 2.5–10 K intervals. Spectra were recorded after a 5-minute equilibration at each temperature. Data were processed using MestreNova version 10.0.2.
NMR experiments of PNA GL-Os **P6**-**P15** were done in 4.5% DMSO by adding DMSO-$d_6$ (20 µL) and $D_2O$ (10 µL) to a G4 DNA solution (170 µL), to achieve a final concentration of 100 µM G4 DNA.

**Taq polymerase STOP assay.** Taq Polymerase stop assay was adapted from Berner et al.[2] DNA templates were annealed to fluorescent labelled primers in 100 mM KCl by heating to 95 °C for 5 minutes followed by slow cooling to room temperature. The indicated compound concentrations were added to 40 nM annealed template in 1x Taq Buffer (10 mM Tris-HCl pH 8.8, 50 mM KCl, Thermo Fisher Scientific), 1.5 mM $MgCl_2$, and 0.05 U/µL Taq Polymerase (Thermo Fisher Scientific). For PNA-based GL-Os, assays were performed both in the presence and absence of DMSO, and no differences in polymerase stalling or full-length product formation were observed. Samples were preincubated on ice (10 minutes) and reactions initiated with the addition of dNTPS (100 µM) and transferring the samples to 37 °C. After 15 min at 37 °C, reactions were stopped by addition of equal volume of 2x stop solution (0.5% SDS, 25 mM EDTA, XC-Dye in Formamide) and separated on a 12% polyacrylamide Tris-Borate-EDTA (TBE) gel containing 25% formamide and 8 M urea. Fluorescent signal was detected with a Typhoon Scanner (Amersham Biosciences). The intensity of the full-length band was quantified using Image Quant TL 10.2 software (GE Healthcare Life Sciences) and compared to sample without compound.
The PNA GL-Os **P6**-**P15** were run in a final concentration of 0.45% DMSO in the assay.

***In vitro* endonuclease digestion.** Stability of oligos against Mung bean nuclease digestion was assessed. 4 µM oligos were incubated with 3 units of enzyme (MBN, NEB) in 1X MBN buffer (MBN) at

30 degrees. At indicated time points, reactions were quenched by adding DNA Gel loading dye (50 mM EDTA, 50 mM HEPES, 30% Glycerol, 0.001% Bromophenol Blue) and heating at 95 degrees. Digestion products were analysed by polyacrylamide gel electrophoresis (PAGE). DNA samples were resolved on 16% TBE gels for 1 h at 100 V in 1X TBE buffer. PNA samples were loaded on 4-20% SDS PAGE gels (Mini-PROTEAN TGX Gels, Bio-Rad) and run for 1 h at 120 V in 1X SDS running buffer (Tris Glycine). TBE gels were stained with Gel red (Biotium), while SDS PAGE gels were stained with Instant Blue (Abcam). Gels were imaged using a ChemiDoc Imaging system (Bio-Rad).

**CD melting temperature assay.** G4 DNA (3 µM) was folded in K-phosphate buffer (10 mM, pH 7.4) containing KCl (3 mM) by heating for 5 minutes at 95°C, then allowed to cool to room temperature. PNA in DMSO was added in a 1:1 ratio, resulting in a final DMSO concentration of 0.3%. CD measurements were performed on a J-1700 Circular Dichroism Spectrophotometer (Jasco International Co. Ltd.). A quartz cuvette with a path length of 1 mm was used for measurements. CD spectra were recorded between λ = 200 – 350 nm with an interval of 0.5 nm and a scan rate of 100 nm/min. Thermal melting was recorded between 25°C and 95°C with increments of 10°C at a speed of 1 °C/min.

**Cell line generation.** To enable cellular uptake and tracking of GL-O internalization, a trivalent GalNAc moiety and a Cy3 fluorophore were incorporated at the 3′-end of an oligonucleotide, which was subsequently conjugated to the G4-ligand as described above. Flp-In™ T-REx™ 293 cells (Thermo Fisher Scientific) were engineered for doxycycline-inducible expression of the asialoglycoprotein receptor 1 (ASGR1). The ASGR1 coding sequence (CDS), flanked by restriction sites, was synthesized by Eurofins and subcloned into the pcDNA5/FRT/TO vector. The Flp-In™ system enables site-specific integration of the gene of interest via Flp recombinase–mediated recombination; its key features are summarized in Figure S2. Briefly, Flp-In™ T-REx™ 293 cells were co-transfected with pcDNA5/FRT/TO-ASGR1 (600 ng) and pOG44 (6 µg) using TurboFect (Thermo Fisher Scientific). Hygromycin-resistant isogenic colonies were pooled and expanded. ASGR1 expression following doxycycline induction (25 ng/mL) was confirmed by Western blot using an anti-ASGR1 antibody (Proteintech; Figure S2).

**Microscopy.** Uptake and intracellular localization of the GalNAc- and Cy3-modified GL-O were assessed by live-cell confocal microscopy. ASGR1-expressing HEK293 cells were seeded onto glass-bottom dishes and induced overnight with doxycycline (25 ng/mL). Cells were then incubated with 0.5 µM Cy3-conjugated GalNAc oligonucleotide for 24 h prior to imaging. Live-cell imaging was performed at the Biochemical Imaging Centre Umeå on a Leica SP8 FALCON confocal microscope equipped with a 63× oil-immersion objective.

***Computational assessment***

**Sequence alignment analysis.** The specificity of 5′ G4 flanking sequences of varying lengths was assessed by aligning these DNA sequences to the human reference genome. The human reference genome (GRCh38) was retrieved from the UCSC Genome Browser,[3] and flanking sequence was obtained from NCBI nucleotide database. FASTA datasets containing flanking sequences of 5–20 bases were generated and each dataset was independently aligned to the human reference genome using Bowtie 2.0,[4] with no mismatch allowed. The aligned reads in SAM files were converted to BAM format and subsequently sorted and indexed using SAMtools[5] with default settings. The final BAM files were used for downstream sequence annotation and comparative analysis of mapping specificity across different flanking lengths.

**Table S1. Protocol for PNA synthesis**

| Step | Reagent/solvent | Volume (µL) | Cycles x Time |
|---|---|---|---|
| Swelling | DCM | 500 | 30 minutes |
| Wash | DMF | 5 x 500 | 5 x 1 minute |
| Deprotection | 20% (v/v) piperidine in DMF | 500 | 2 x 3 minutes |
| | | | 1 x 9 minutes |
| Wash | DMF | 5 x 500 | 5 x 1 minute |
| Coupling | Monomer in DMF | 1 x 200 | 60 minutes |
| | HATU in NMP | 1 x 210 | |
| | DIPEA in DMF | 1 x 100 | |
| Wash | DMF | 5 x 500 | 5 x 1 minute |
| Capping | Ac2O 50% (v/v) in DCM | 1 x 200 | 40 minutes |
| | DIPEA | 1 x 100 | |
| Wash | DMF | 5 x 500 | 5 x 1 minute |
| Conjugation of G4-Ligand | G4-Ligand in NMP | 1 x 200 | 24 h |
| | HATU in NMP | 1 x 210 | |
| | DIPEA in DMF | 1 x 100 | |

**Table S2. G4 DNA templates**

| Used in | G4 DNA | Sequence (5´-3´) |
|---|---|---|
| MST assay | *c-MYC* Pu24T | [Cy5]-AGT CAC TGA ATT GGA CGT GA TGA GGG TGG TGA GGG TGG GGA AGG |
| NMR Assay | *c-MYC* Pu24T | AGT CAC TGA ATT GGA CGT GA TGA GGG TGG TGA GGG TGG GGA AGG |
| Taq Polymerase Stop Assay | c-MYC Pu24T | AGT CA CTG AAT TGG ACG TGA TGA GGG TGG TGA GGG TGG GGA AGG<br>CAC GTG AGT TGA GTG GAG TTG GAA GTA GGC ATA CCC CTA T |
| Polymerase Stop assay | 15-nt TET primer | [TET]-GCC TAC TTC CAA CTC |
| Polymerase Stop assay | 25-nt TET primer | [TET]-ATA GGG GTA TGC CTA CTT CCA ACT C |

G4 forming sequence (red), nucleotide gap (green), primer site (blue)

**Table S3. GL-O conjugates synthesized for this study**

| GL-O name | Conjugation method | Oligo 5´modification | Sequence length | Sequence (5´-3´) |
|---|---|---|---|---|
| **GL-O D6** | SPAAC | Amine-C6 | 6-nt | *TmCAmCGT |
| **GL-O D8** | SPAAC | Amine-C6 | 8-nt | *TmCAmCGTmCmC |
| **GL-O D10** | SPAAC | Amine-C6 | 10-nt | *TmCAmCGTmCmCAA |
| **GL-O D12** | SPAAC | Amine-C6 | 12-nt | *TmCACGTmCmCAATT |
| **GL-O D14** | SPAAC | Amine-C6 | 14-nt | *TmCAmCGTmCmCAATTmCA |
| **GL-O D15** | SPAAC | Amine-C6 | 15-nt | *TmCAmCGTmCmCAATTmCAG |
| **GL-O D16** | SPAAC | Amine-C6 | 16-nt | *TmCAmCGTmCmCAATTmCAGT |
| **GL-O D18** | SPAAC | Amine-C6 | 18-nt | *TmCAmCGTmCmCAATTmCAGTGA |
| **GL-O D20** | SPAAC | Amine-C6 | 20-nt | *TmCAmCGTmCmCAATTmCAGTGAmCT |
| **GL-O P6** | Amide | Amine-C6 | 6-nt | TCACGT |
| **GL-O P8** | Amide | Amine-C6 | 8-nt | TCACGTCC |
| **GL-O P10** | Amide | Amine-C6 | 10-nt | TCACGTCCAA |
| **GL-O P12** | Amide | Amine-C6 | 12-nt | TCACGTCCAATT |
| **GL-O P15** | Amide | Amine-C6 | 15-nt | TCACGTCCAATTCAG |
| **GL-O TM1-3** | SPAAC | Amine-C6 | 15-nt | *mCTTmCGTmCmCAATTmCAG |
| **GL-O TM4-6** | SPAAC | Amine-C6 | 15-nt | *TmCATTmCmCmCAATTmCAG |
| **GL-O TM7-9** | SPAAC | Amine-C6 | 15-nt | *TmCAmCGTTTTATTmCAG |
| **GL-O TM10-12** | SPAAC | Amine-C6 | 15-nt | *TmCAmCGTmCmCATmCmCmCAG |
| **GL-O TM13-15** | SPAAC | Amine-C6 | 15-nt | *TmCAmCGTmCmCAATTTTT |
| **GL-O D15 PS-Cy3-GalNac** | SPAAC | Amine-C6 | 15-nt | *Tm*C*A*mC*G*T*mC*mC*A*A*T*T*mC*A*G-Cy3-3´-valent GalNAc |

mC stands for methylated cytidine. * Phosphorothioate (PS) modification.

**Table S4. Yields and mass of the GL-O conjugates**

| GL-O name | Yield (%) | Calc. MS (g/mol) | Found MS (m/z) |
|---|---|---|---|
| **GL-O D6** | 66 | 2611,2446 | 2608,7248 |
| **GL-O D8** | 82 | 3217,6661 | 3215,8500 |
| **GL-O D10** | 57 | 3844,0856 | 3841,9774 |
| **GL-O D12** | 33 | 4452,4751 | 4452,0618 |
| **GL-O D14** | 43 | 5068,8957 | 5068,1141 |
| **GL-O D15** | 48 | 5398,1044 | 5397,2420 |
| **GL-O D16** | 30 | 5702,2992 | 5699,2611 |
| **GL-O D18** | 33 | 6344,7177 | 6344,3184 |
| **GL-O D20** | 54 | 6952,1232 | 6951,3930 |
| **GL-O P6** | 2 | 2289,0450 | 2290,0500 |
| **GL-O P8** | 6 | 2791,2788 | 2792,2542 |
| **GL-O P10** | 5 | 3341,4749 | 3342,4775 |
| **GL-O P12** | 5 | 3873.6779 | 3875,6694 |
| **GL-O P15** | 4 | 4691.0008 | 4692,9976 |
| **GL-O TM1-3** | 38 | 5389,0894 | 5373,1840 |
| **GL-O TM4-6** | 33 | 5373,0904 | 5390,1644 |
| **GL-O TM7-9** | 31 | 5391,0574 | 5388,2091 |
| **GL-O TM10-12** | 33 | 5361,1234 | 5386,1752 |
| **GL-O TM13-15** | 46 | 5365,0594 | 5364,1672 |
| **GL-O D15 PS-Cy3-GalNAc** | 55 | 7918.0527 | 7917.9102 |

**Table S5.** Three site complementarity of a 13-nt oligonucleotide binding sequence of *c-Myc* Pu27.

| Chromosome | Matching site (gene) | Functions | Gene ID | Start-end position | Annotation | Sequence (flanking ±25)* |
|---|---|---|---|---|---|---|
| 8 | MYC proto-oncogene | - | ENSG00000136997 | 127735951 - 127735964 | Promoter | CGCCCTCTGCTTTGGGAACCCGGGA**GGGGCGCTTATGG**GGAGGGTGGGGAGGGTGGGGAAGGT |
| 1 | LMX1A (LIM homeobox transcription factor 1 alpha) | Transcription factor regulates gene expression involved in the development of neurons and pancreas. | ENSG00000162761 | 165213724 - 165213737 | Promoter | AAGGAACTGCTGAGGAAGGCAAGGA**CCATAAGCGCCCC**AAACGTCCGAGAACCATCTTGACAA (antisense) TTGTCAAGATGGTTCTCGGACGTTT**GGGGCGCTTATGG**TCCTTGCCTTCCTCAGCAGTTCCTT |
| 12 | ABTB3 (ankyrin repeat and BTB domain containing 3) | Specifically expressed in cortical and hippocampal inhibitory interneurons and support in synaptic functions. | ENSG00000151136 | 107523249 - 107523262 | Intronic | AGTCAGTAGCTGATCCAAGCCATAG**GGGGCGCTTATGG**CCACTAGAGAGCTACTTTCCTGGCA |
| 2 | CASC11 (LncRNA) | Cancer susceptibility candidate involved in gene regulation and found to be overexpressed in various cancers. | - | 17657528-17657585 | Intergenic | AAACGAAGTGAACTGTATCT**GGGGCGCTTATGG**AAAACAAACCCTCTAGCAGACTCAC |

*Sequence highlighted in red and G4-forming sequence is underlined.

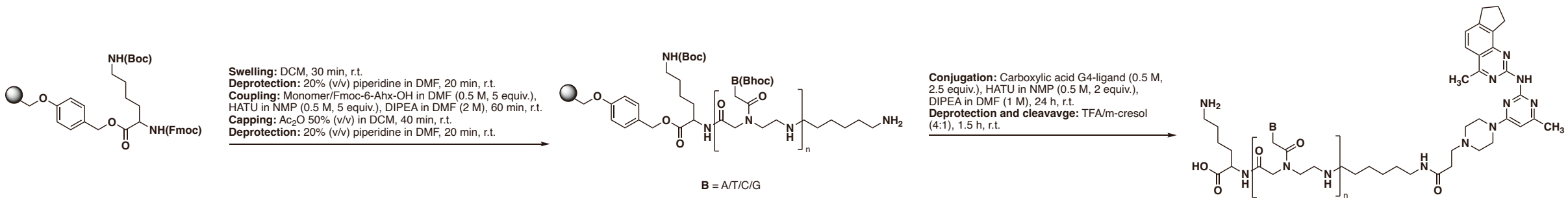

**Figure S1.** Scheme of PNA synthesis and G4-ligand conjugation.

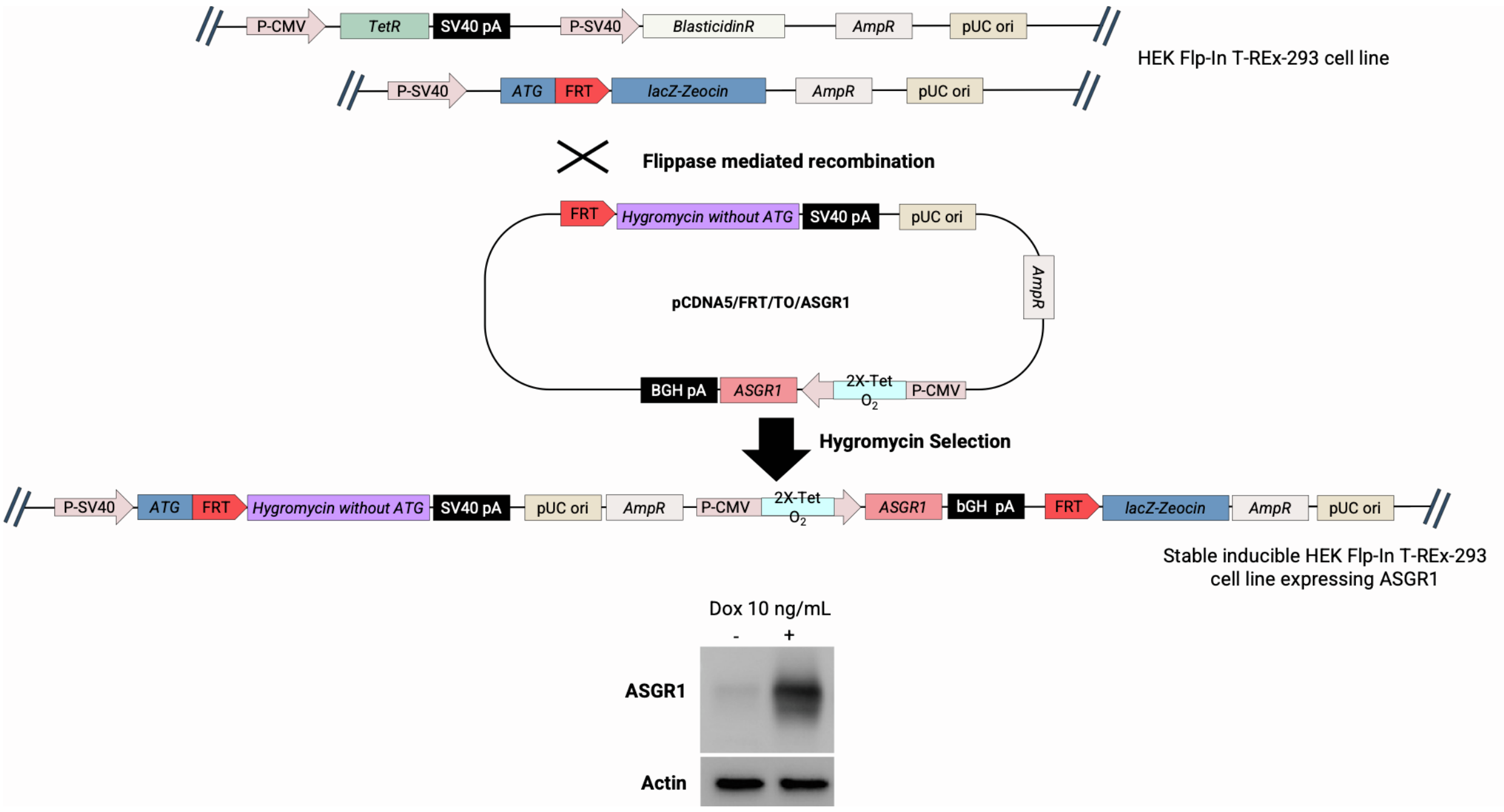


**Figure S2.** Schematic representation of the generation of a ASGR1-expressing cell line used to analyse GalNAc-mediated cellular uptake.

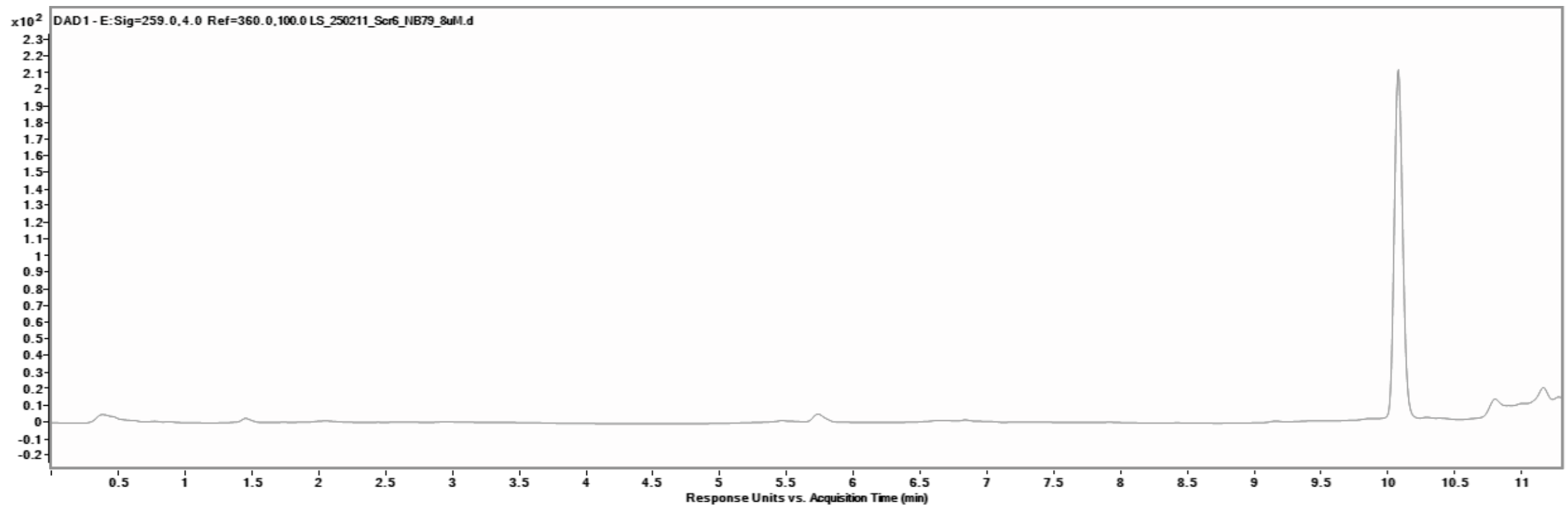


**Figure S3.** UV chromatogram at 259 nm of **GL-O D6**.

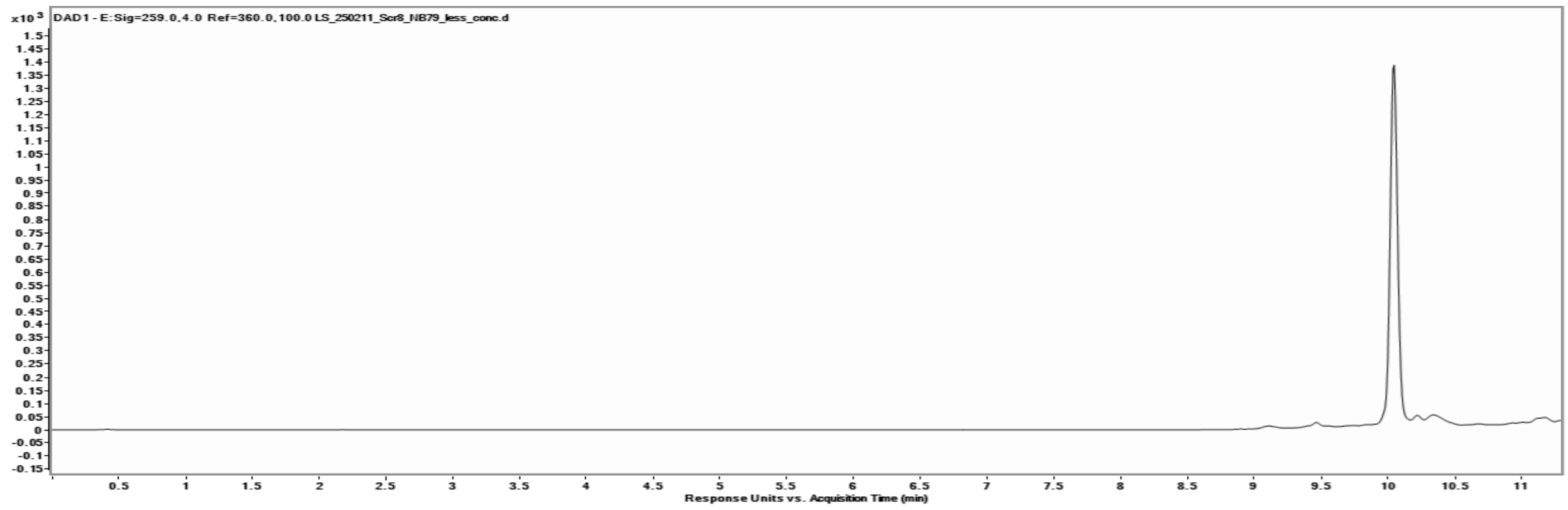


**Figure S4.** UV chromatogram at 259 nm of **GL-O D8**.

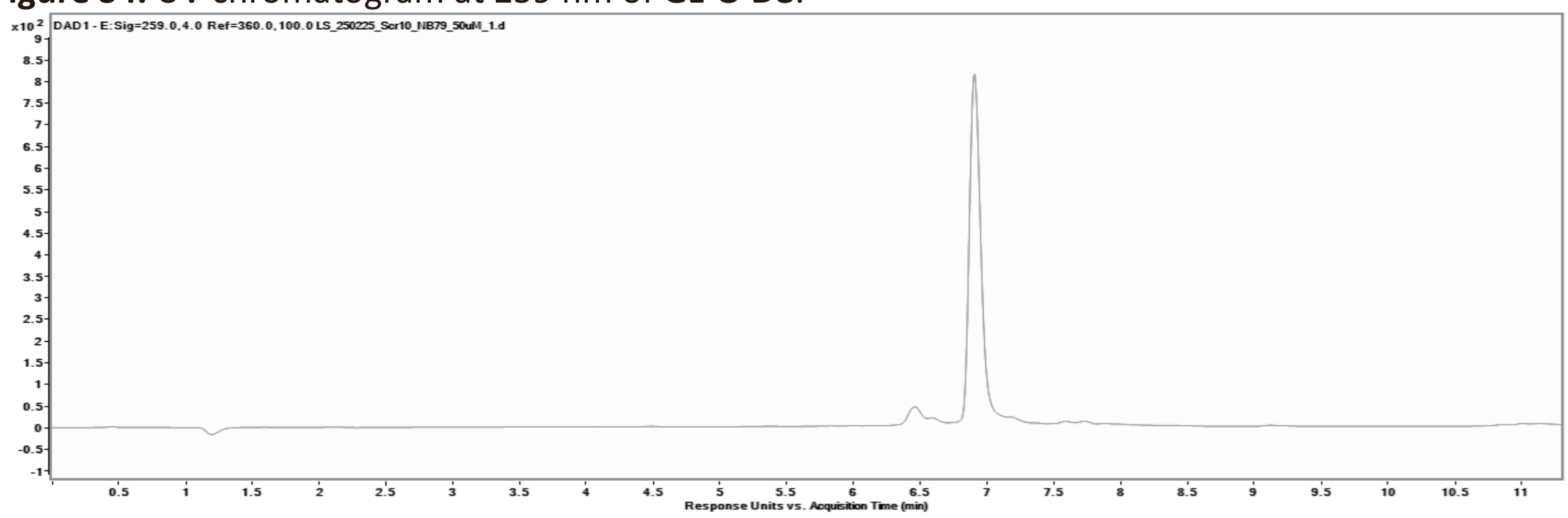


**Figure S5.** UV chromatogram at 259 nm of **GL-O D10**.

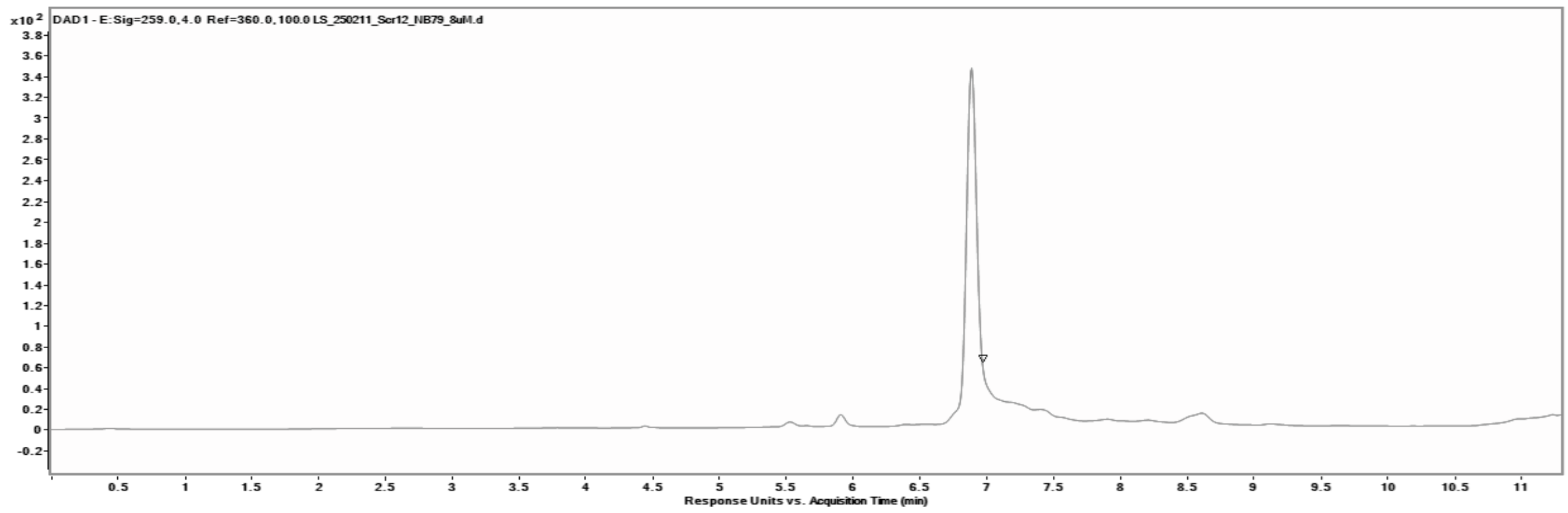


**Figure S6.** UV chromatogram at 259 nm of **GL-O D12**.

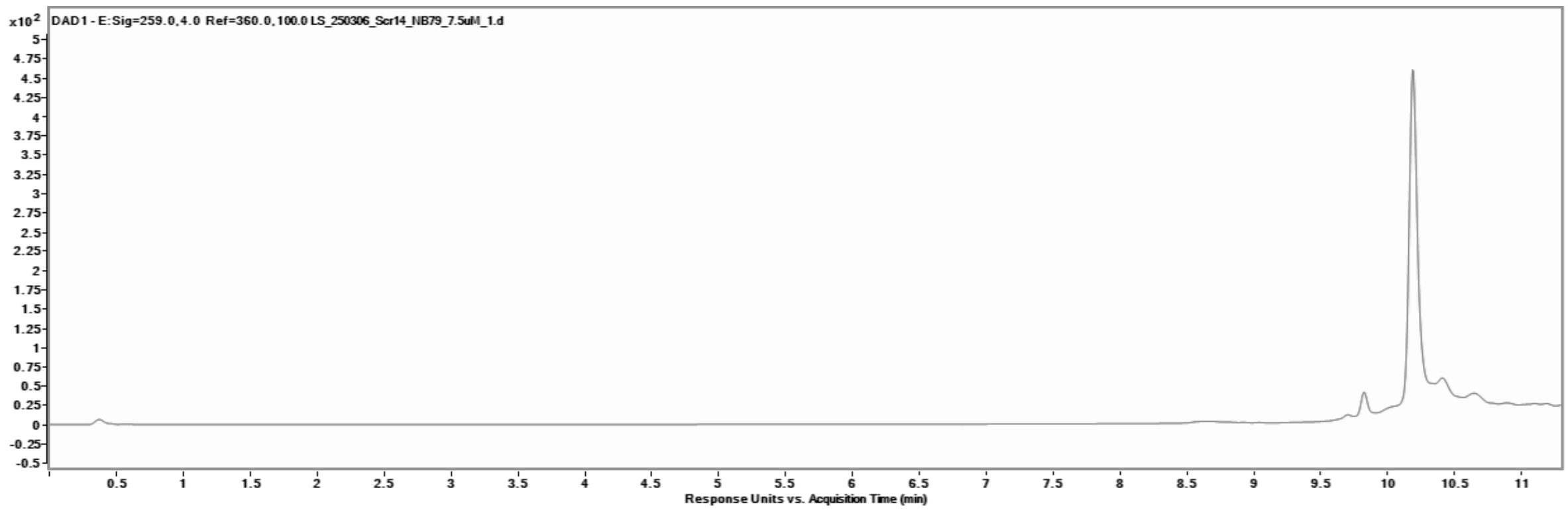


**Figure S7.** UV chromatogram at 259 nm of **GL-O D14**.

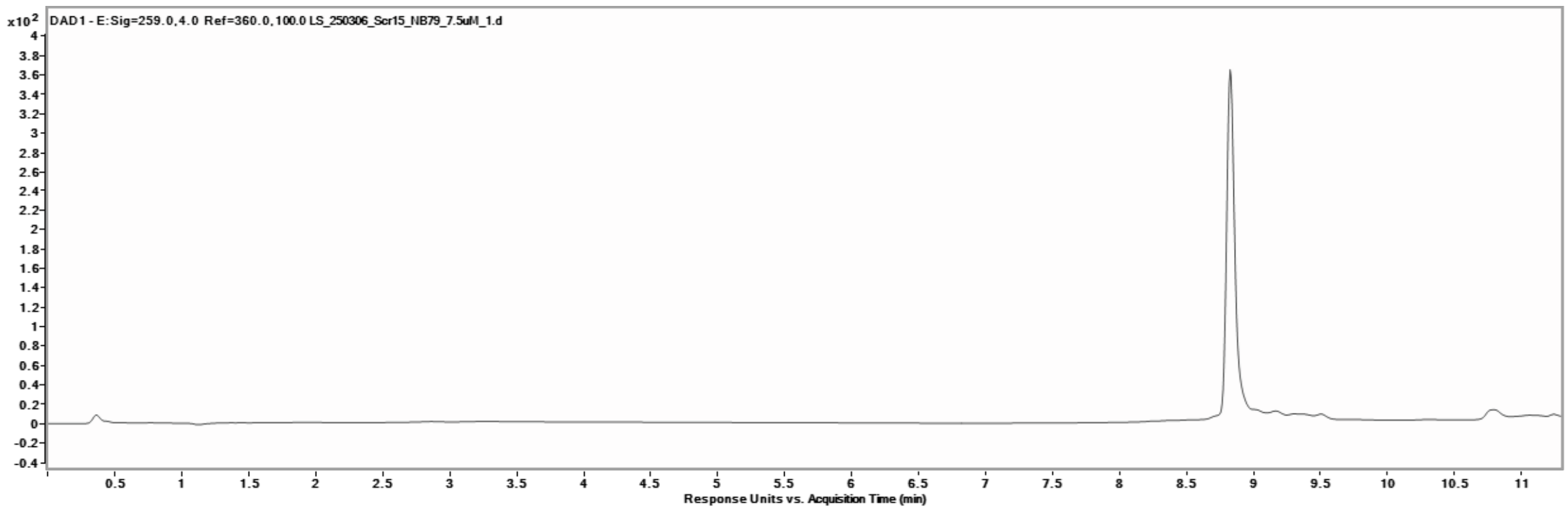


**Figure S8.** UV chromatogram at 259 nm of **GL-O D15**.

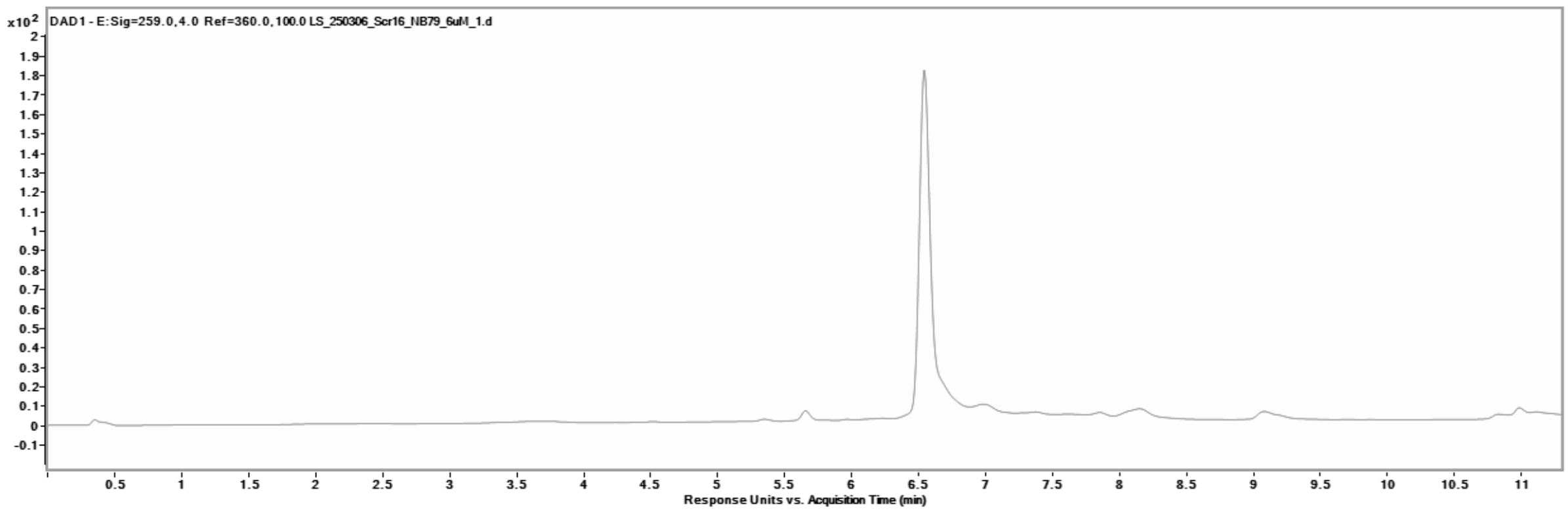


**Figure S9.** UV chromatogram at 259 nm of **GL-O D16**.

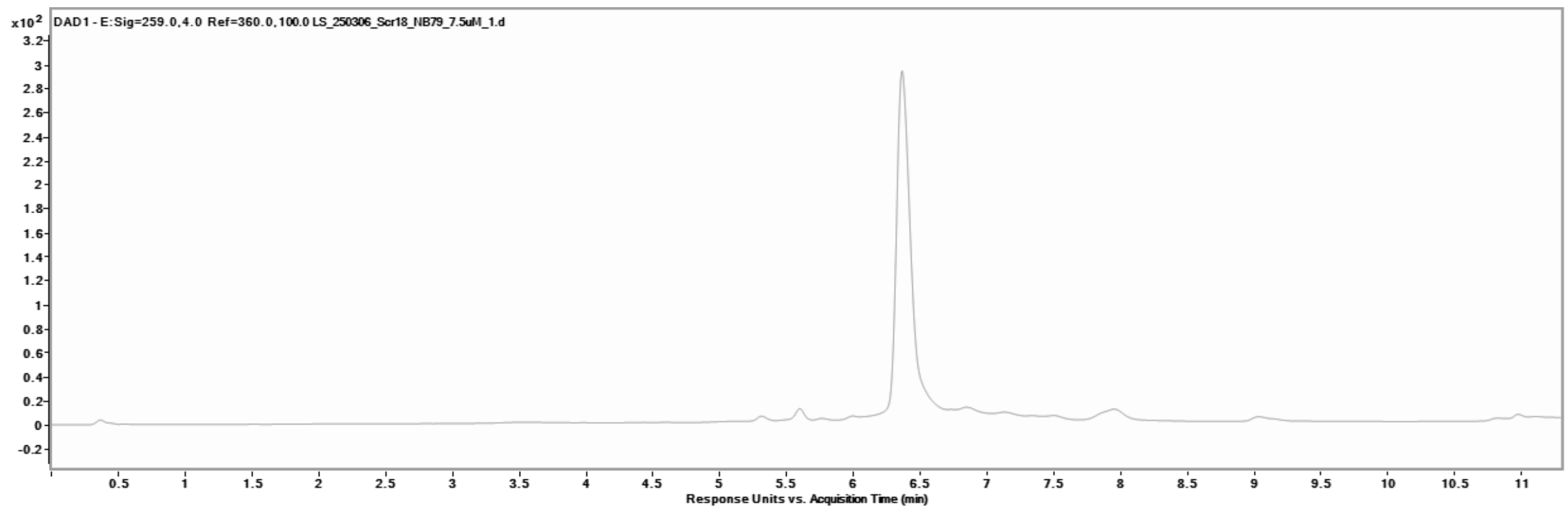


**Figure S10.** UV chromatogram at 259 nm of **GL-O D18**.

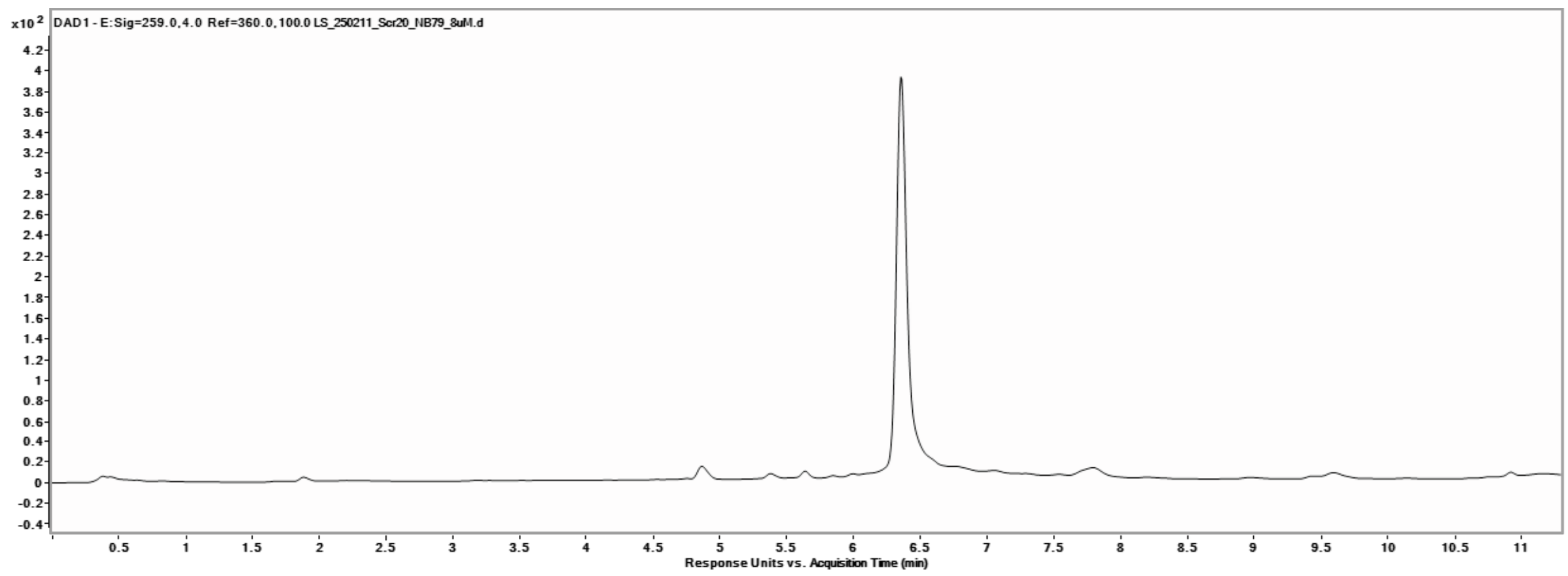


**Figure S11.** UV chromatogram at 259 nm of **GL-O D20**.

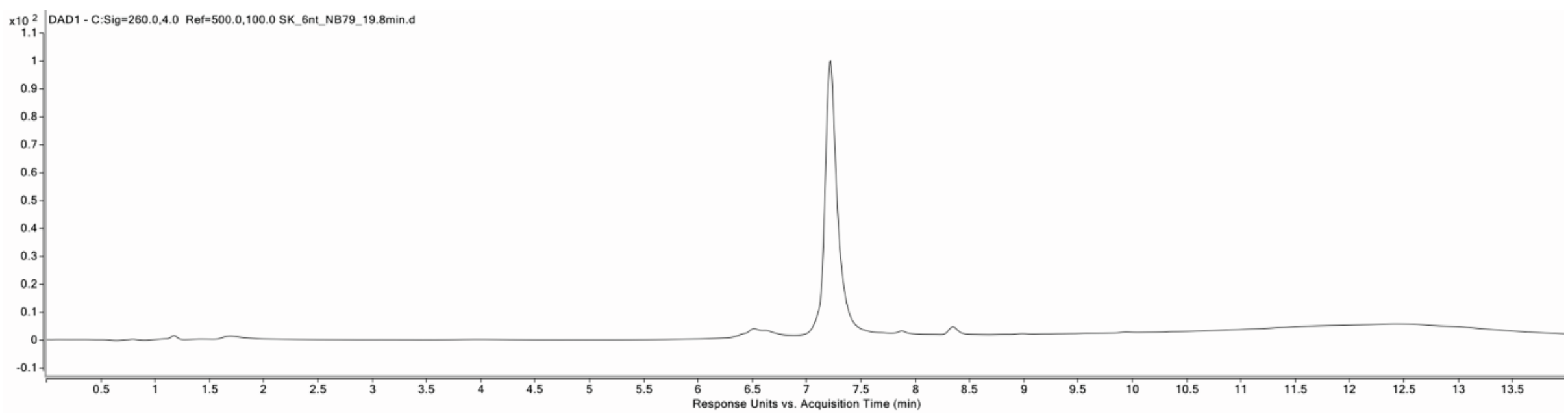


**Figure S12.** UV chromatogram at 259 nm of **GL-O P6**.

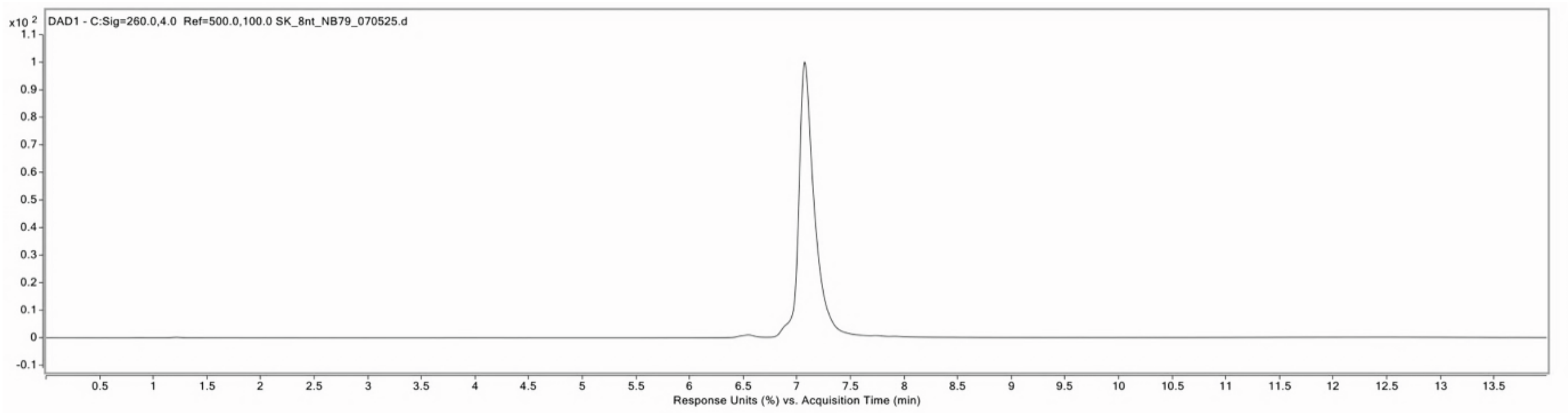


**Figure S13.** UV chromatogram at 259 nm of **GL-O P8**.

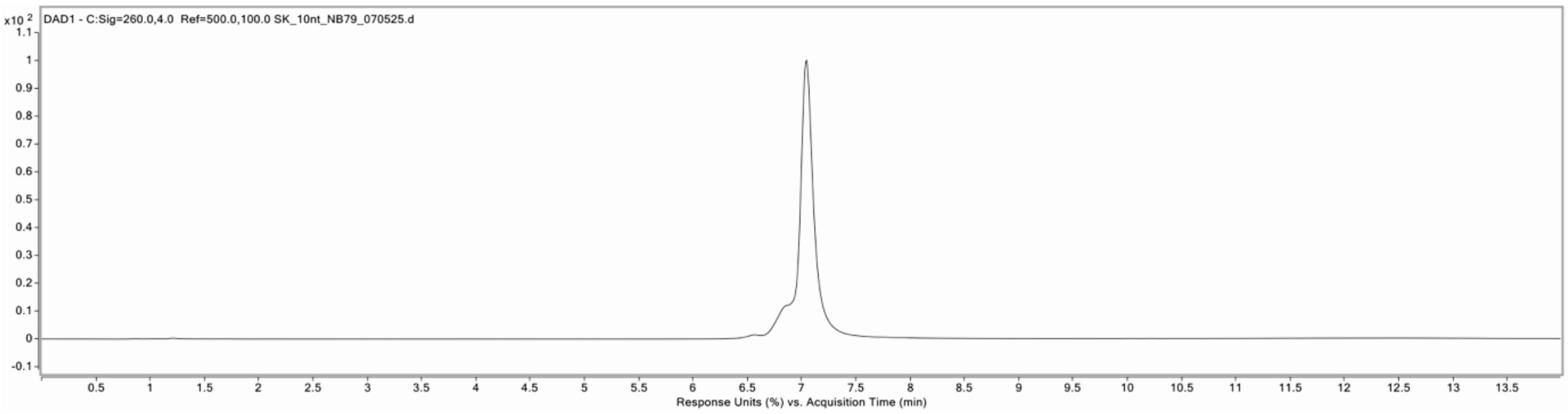


**Figure S14.** UV chromatogram at 259 nm of **GL-O P10**.

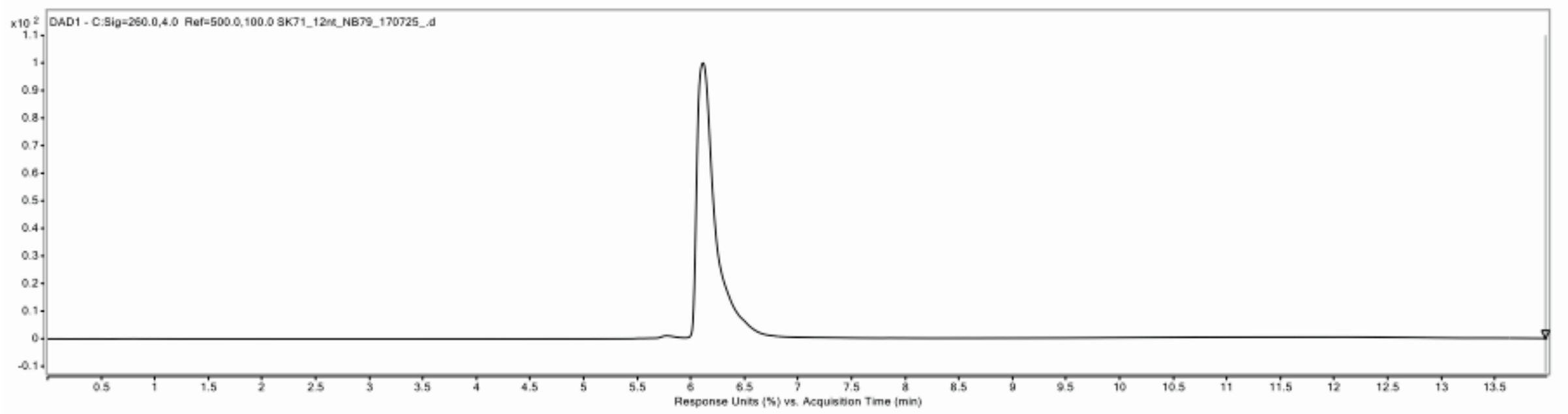


**Figure S15.** UV chromatogram at 259 nm of **GL-O P12**.

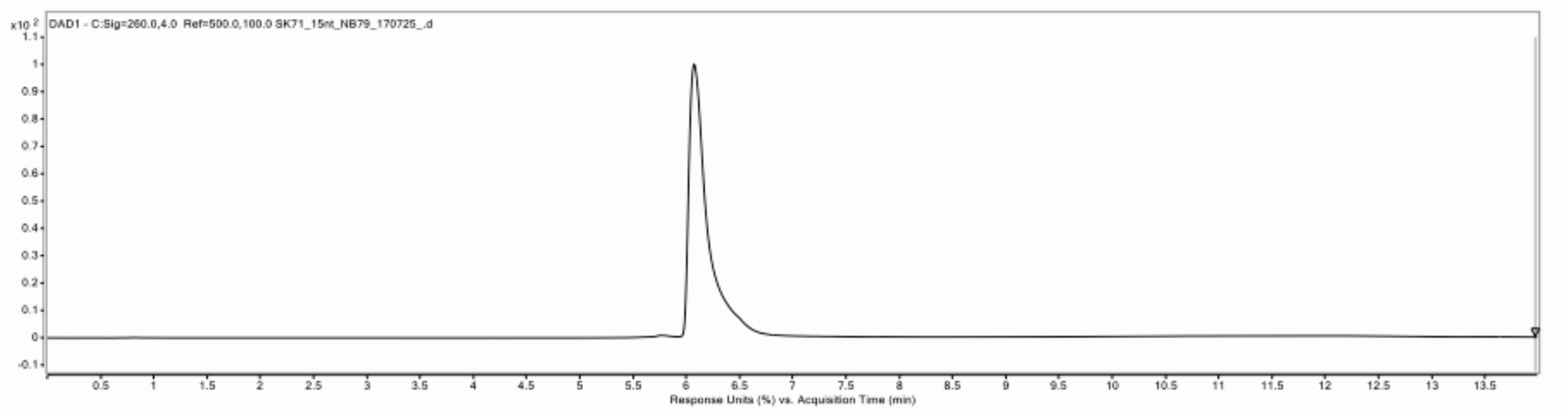


**Figure S16.** UV chromatogram at 259 nm of **GL-O P15**.

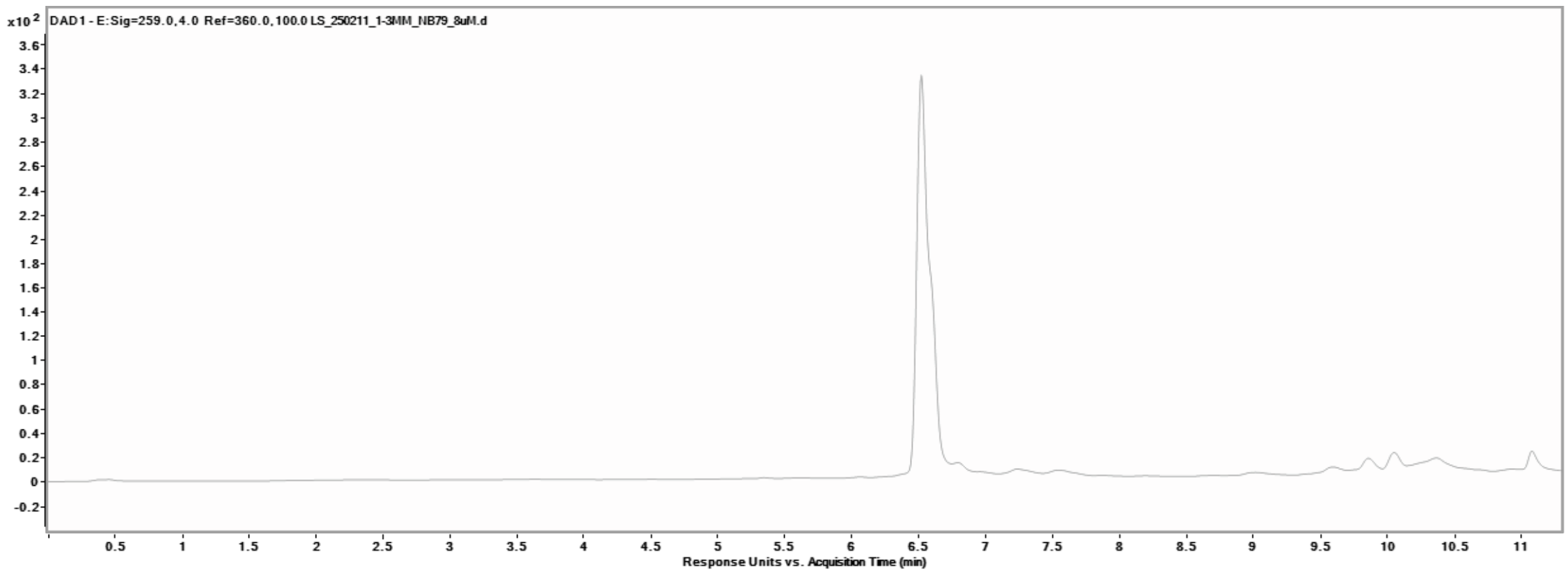


**Figure S17.** UV chromatogram at 259 nm of **GL-O TM1-3.**

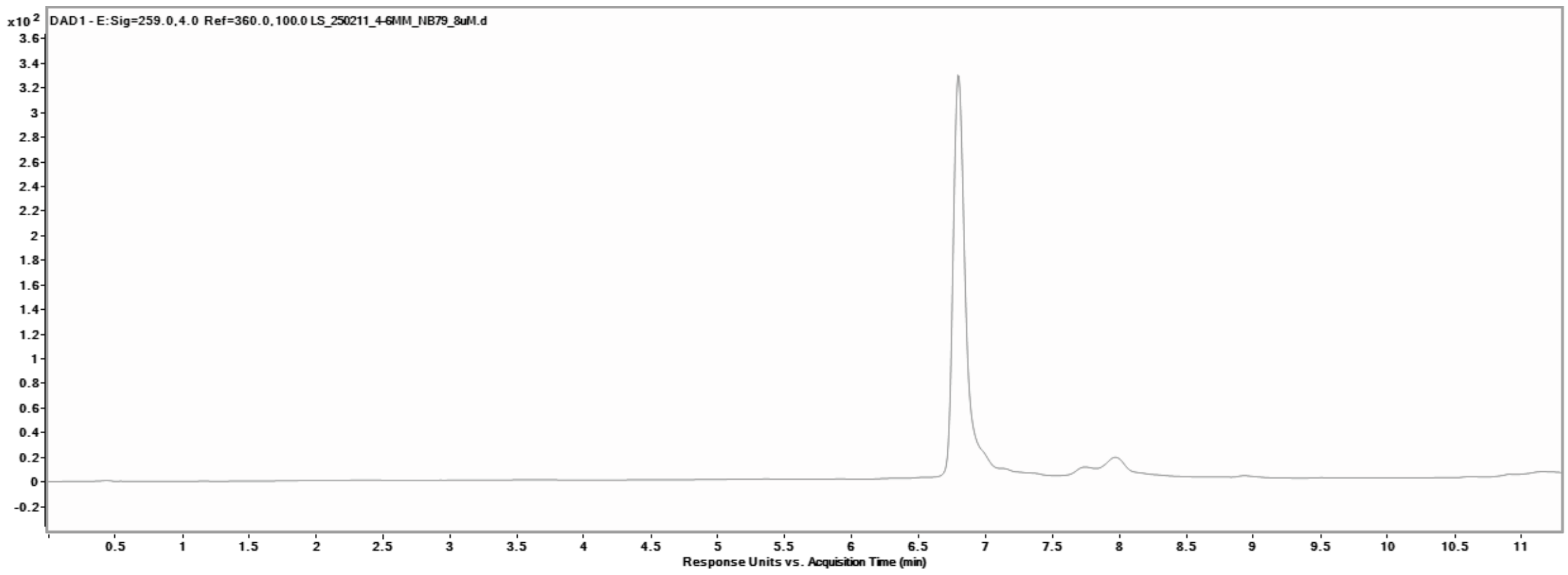


**Figure S18.** UV chromatogram at 259 nm of **GL-O TM4-6.**

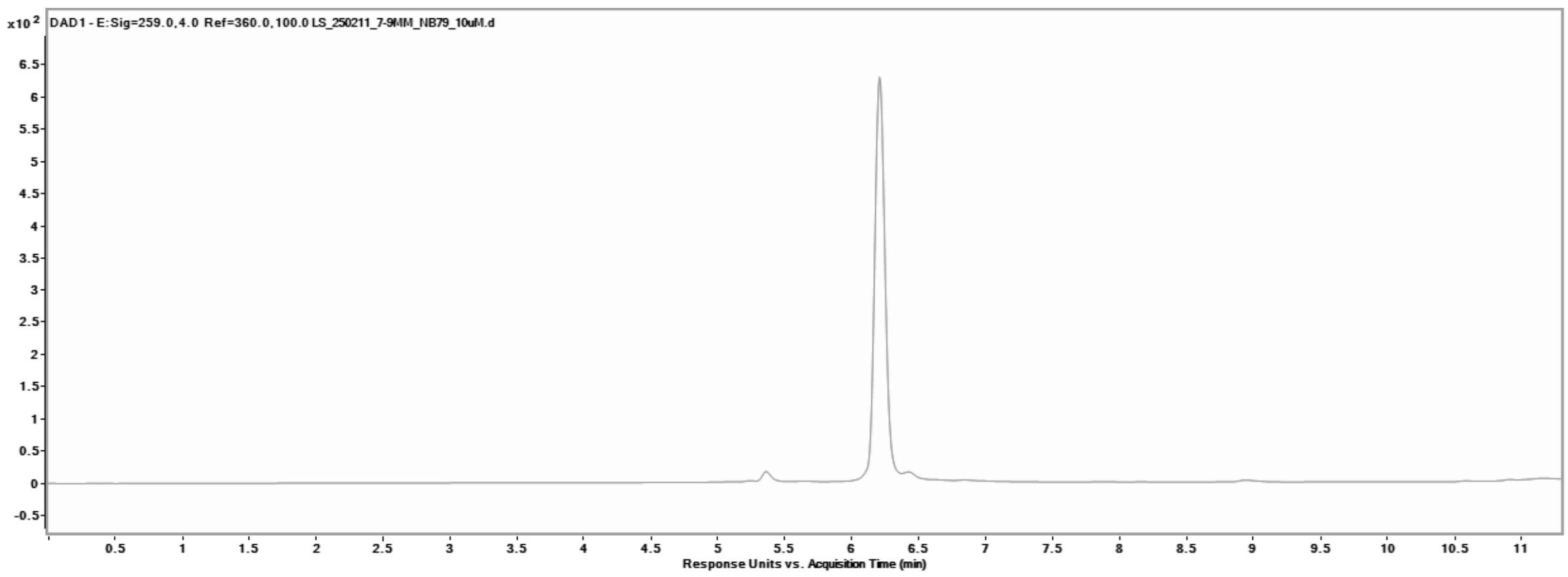


**Figure S19.** UV chromatogram at 259 nm of **GL-O TM7-9.**

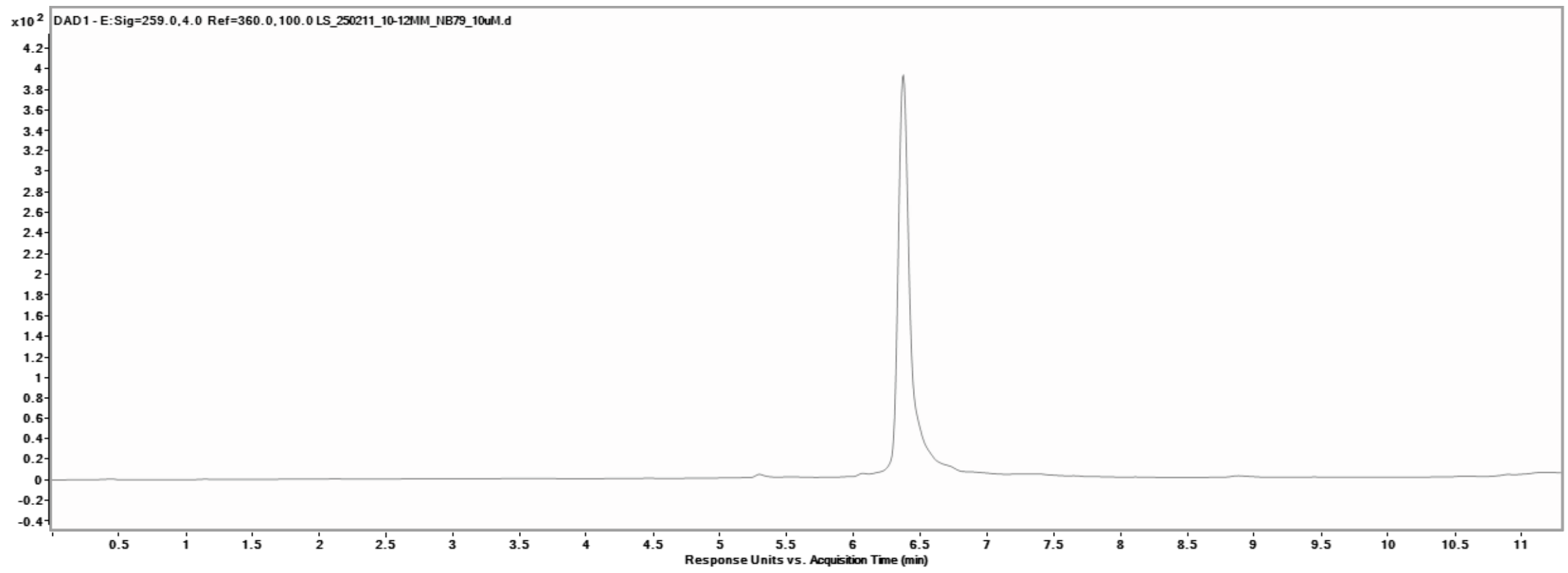


**Figure S20.** UV chromatogram at 259 nm of **GL-O TM10-12.**

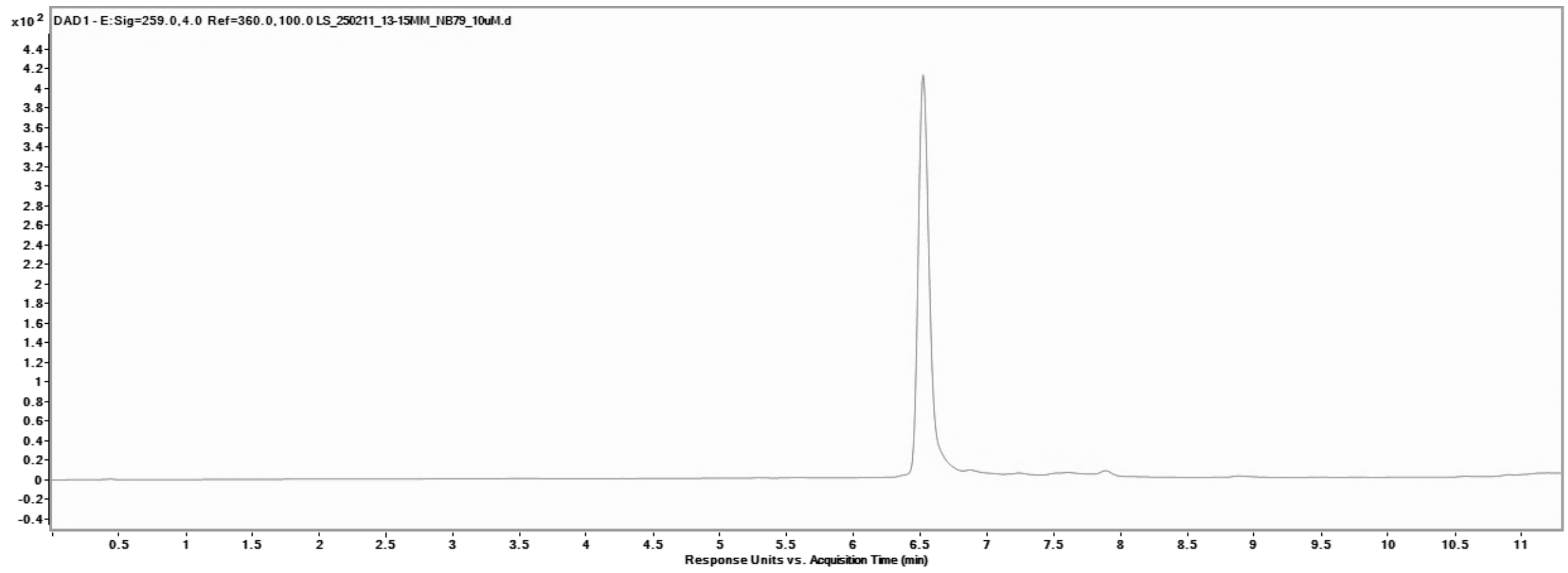


**Figure S21.** UV chromatogram at 259 nm of **GL-O TM13-15.**

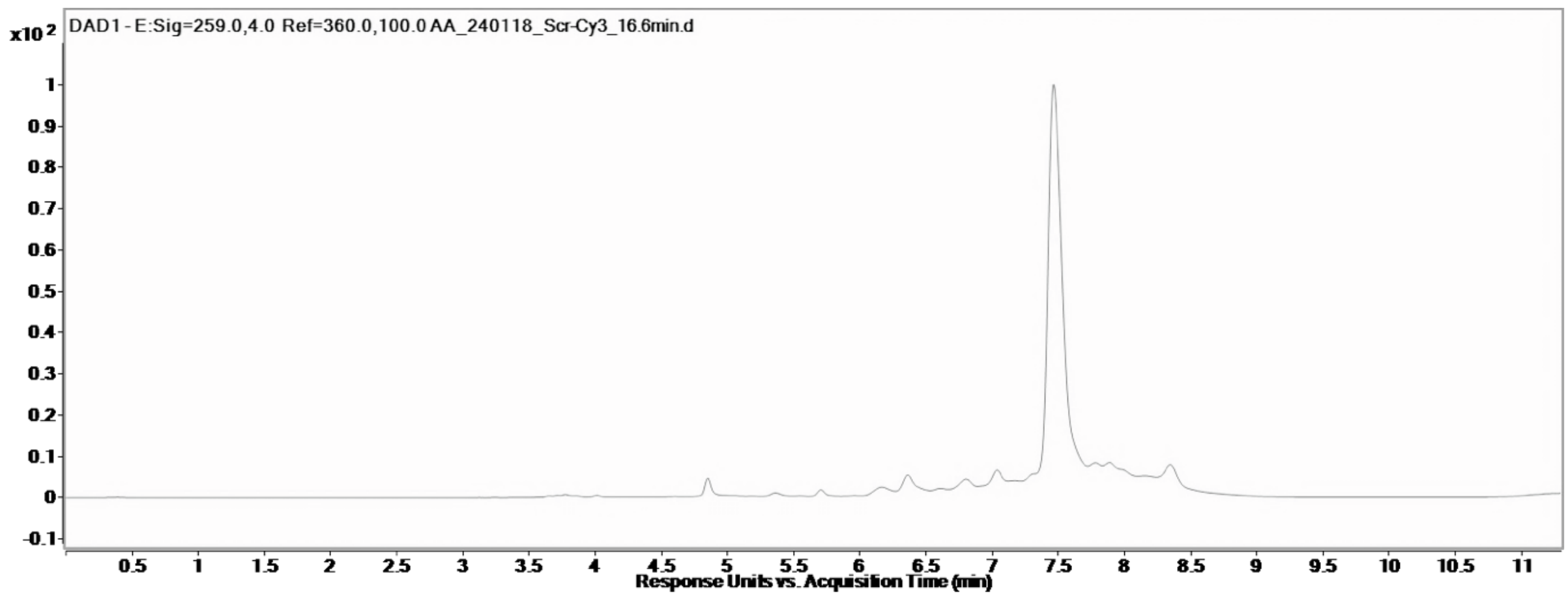


**Figure S22.** UV chromatogram at 259 nm of **GL-O D15-PS-Cy3-GalNAc.**

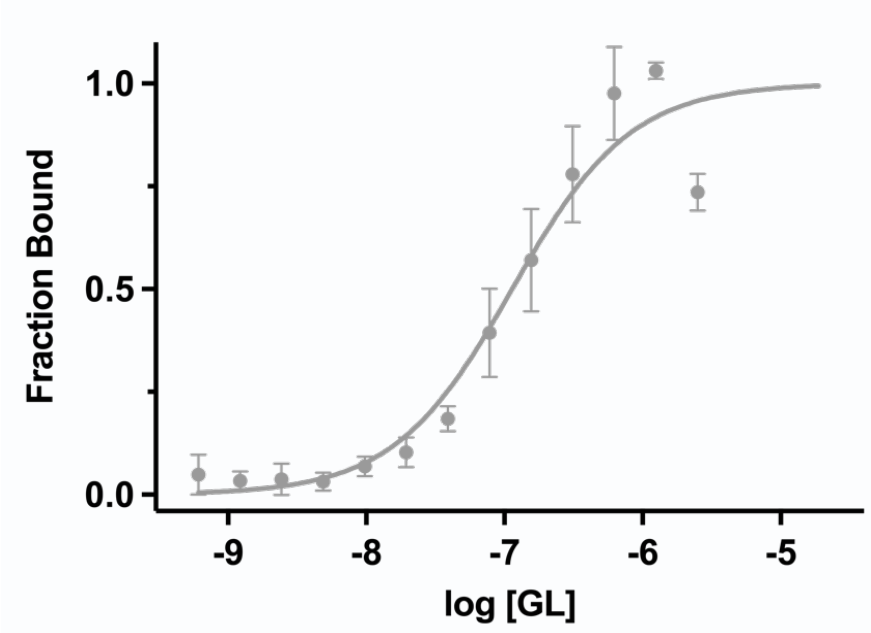


| Compound | $K_D$ (µM) |
|---|---|
| G-Ligand ● | 0.15 ± 0.02 |

**Figure S23.** Binding affinity of the G4-ligand (GL). Dose-response curves obtained using MST where the GL was serial diluted to the G4 DNA 5´-labelled with a fluorescent tag. Dissociation constants ($K_D$) values and error bars correspond to two independent measurements.

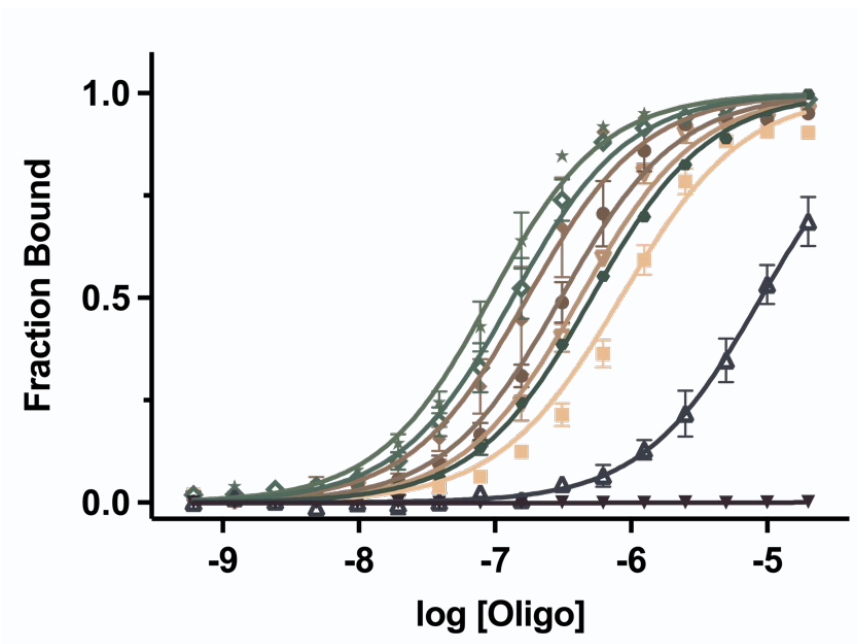


| Compound | $K_D$ (µM) |
|---|---|
| Varying length - DNA | |
| Oligo D6 ▼ | No binding |
| Oligo D8 △ | 9.16 ± 2.24 |
| Oligo D10 ⬢ | 0.50 ± 0.01 |
| Oligo D12 ◇ | 0.13 ± 0.03 |
| Oligo D14 ★ | 0.08 ± 0.02 |
| Oligo D15 ● | 0.30 ± 0.06 |
| Oligo D16 ◆ | 0.17 ± 0.07 |
| Oligo D18 ▽ | 0.41 ± 0.05 |
| Oligo D20 ■ | 0.93 ± 0.12 |

**Figure S24.** Binding affinities of the oligonucleotides **D6**-**D20**. Dose-response curves obtained using MST where the oligonucleotides without G4-ligands (still contains a 5´hexylamine). **D6**-**D20** were serial diluted to the G4 DNA 5´-labelled with a fluorescent tag. Dissociation constants ($K_D$) values and error bars correspond to two independent measurements.

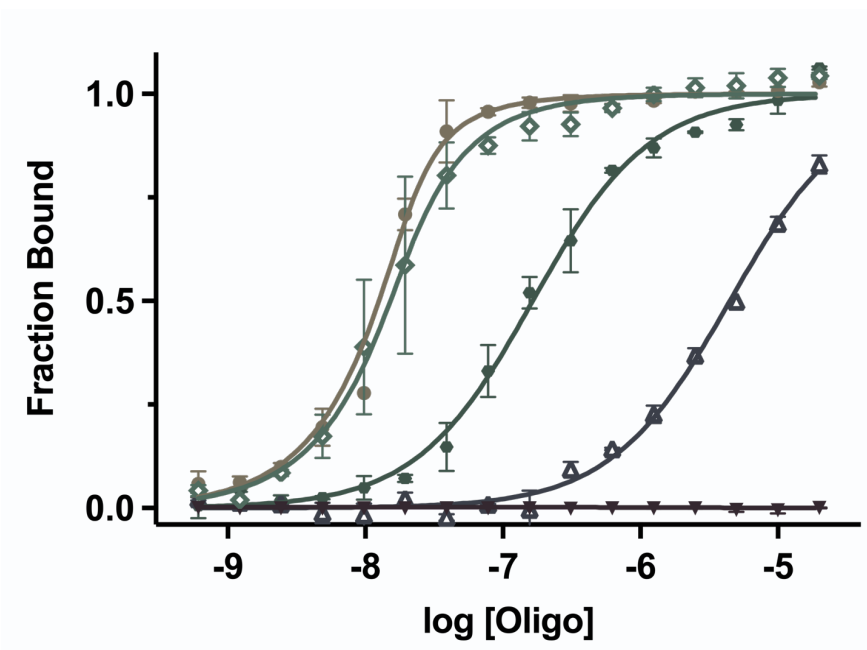


| Compound | $K_D$ (µM) |
|---|---|
| Varying length - PNA | |
| Oligo P6 ▼ | No binding |
| Oligo P8 △ | 4.46 ± 0.15 |
| Oligo P10 ⬢ | 0.16 ± 0.04 |
| Oligo P12 ◇ | 7 ± 6 (nM) |
| Oligo P15 ● | 3 ± 2 (nM) |

**Figure S25.** Binding affinities of the oligonucleotides **P6**-**P15**. Dose-response curves obtained using MST where the oligonucleotides without G4-ligands (still contains a 5´hexylamine). **P6**-**P15** were serial diluted to the G4 DNA 5´-labelled with a fluorescent tag. Dissociation constants ($K_D$) values and error bars correspond to two independent measurements.

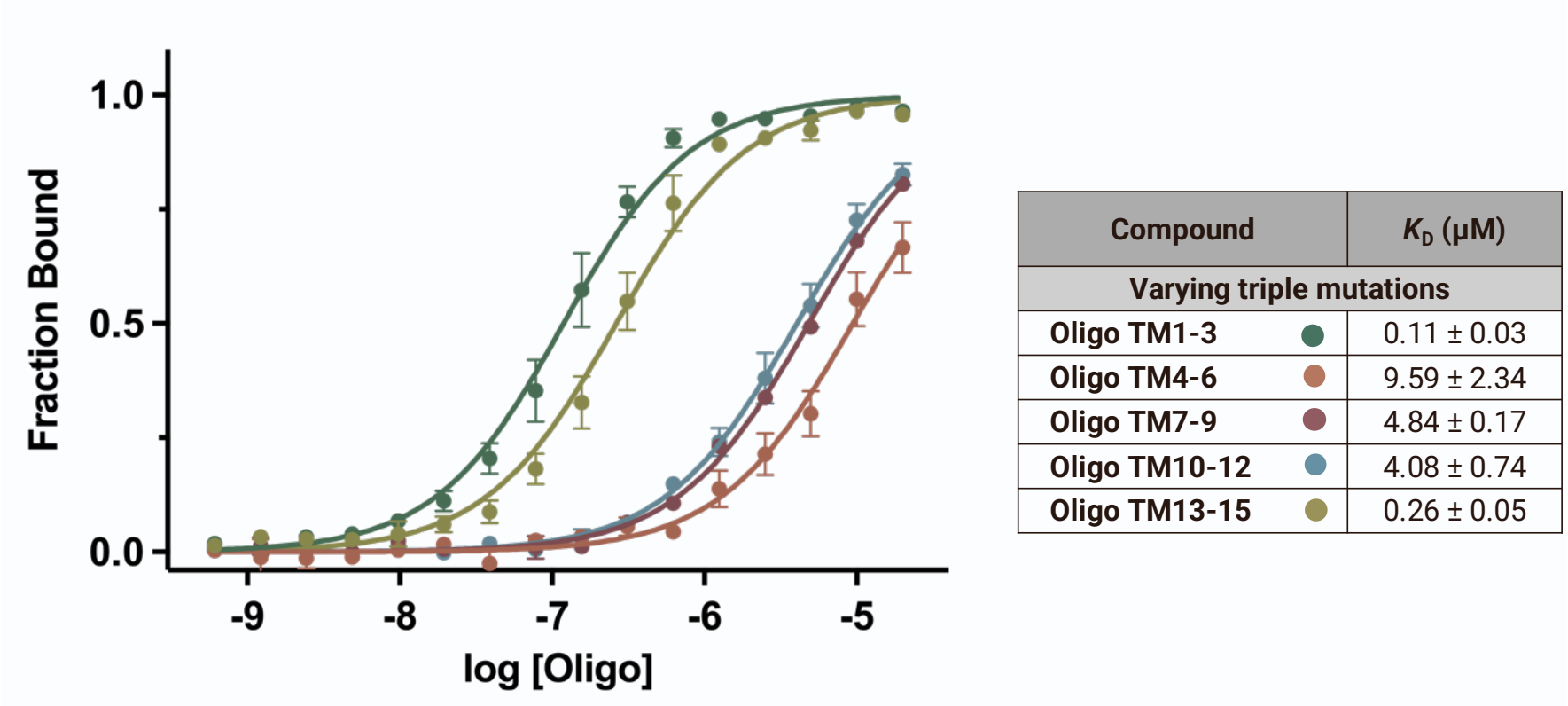


**Figure S26.** Binding affinities of the oligonucleotides **TM1-3, 4-6, 7-9, 10-12, 13-15.** Dose-response curves obtained using MST where the oligonucleotides without G4-ligands (still contains a 5´hexylamine). **TM1-3, 4-6, 7-9, 10-12, 13-15** were serial diluted to the G4 DNA 5´-labelled with a fluorescent tag. Dissociation constants ($K_D$) values and error bars correspond to two independent measurements.

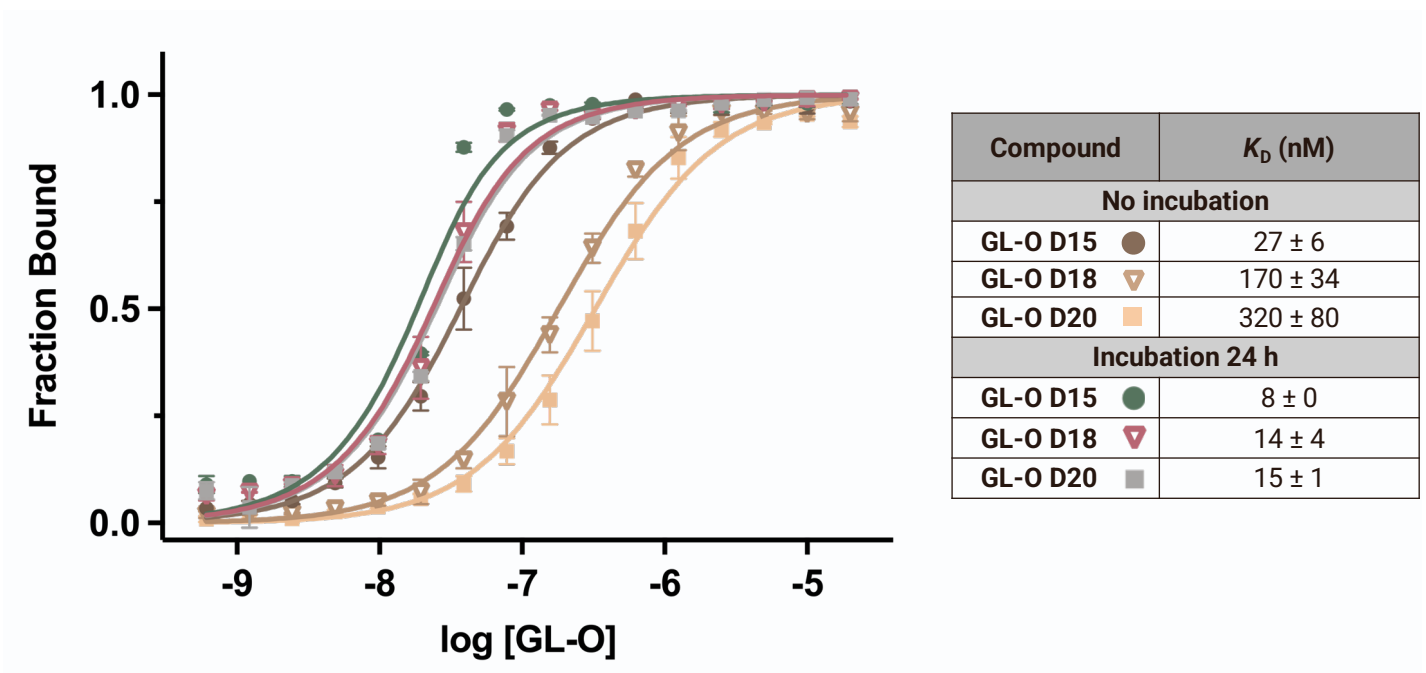


**Figure S27.** Binding affinities of the GL-Os **D15-D20** with or without incubation in room temperature for 24 h. Dose-response curves obtained using MST. **D15-D20** were serial diluted to the G4 DNA 5´-labelled with a fluorescent tag. Dissociation constants ($K_D$) values and error bars correspond to two independent measurements.

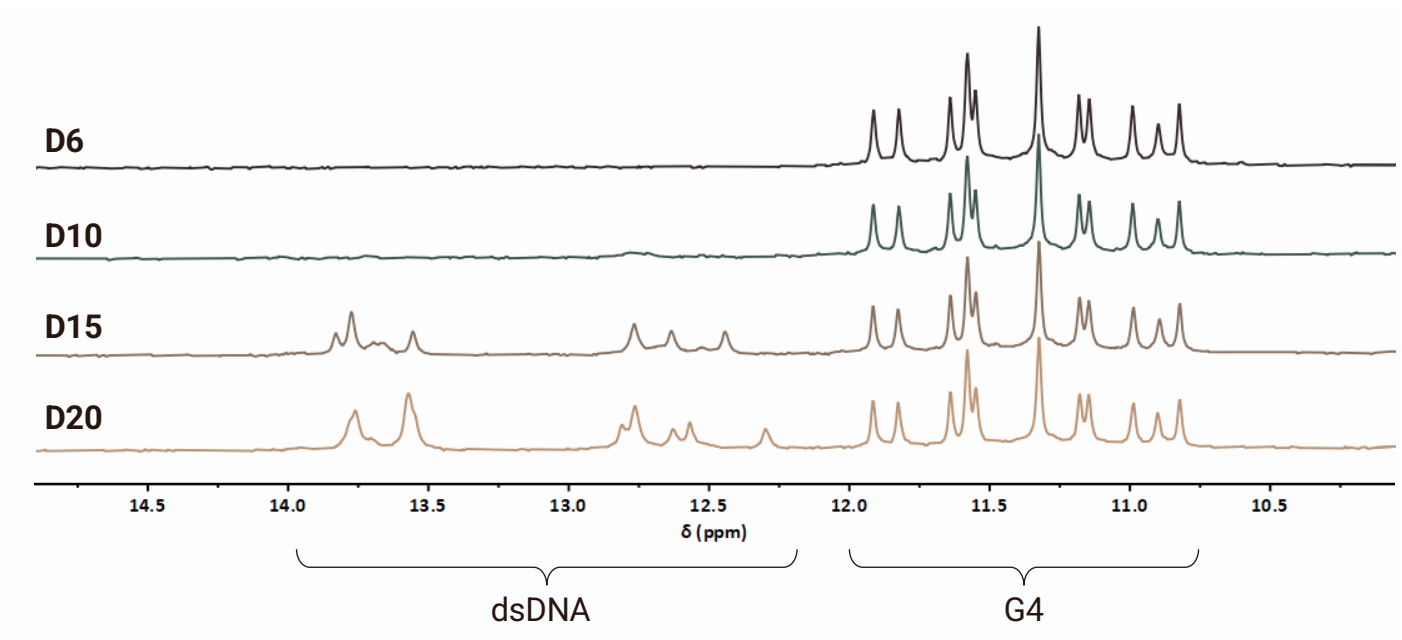


**Figure S28.** Binding interaction of the oligonucleotides **D6-D20** (still contains a 5´hexylamine) measure using $^{1}$H NMR. Spectra were recorded of *c-MYC* Pu24T with complementary flanking sequence in a 1:1 molar ratio at 25 °C. G4 imino signals appear between 10-12 ppm and double-stranded DNA signals appear between 12-14 ppm.

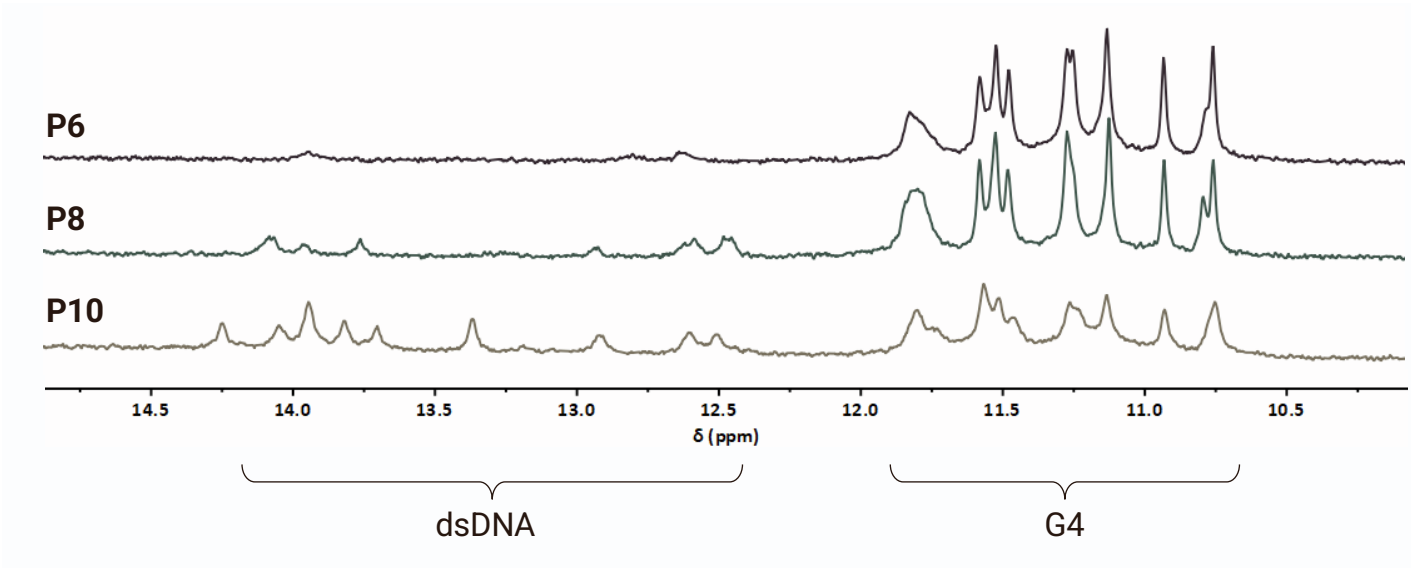


**Figure S29.** Binding interaction of the oligonucleotides **P6**-**P20** (still contains a 5´hexylamine) measure using $^{1}$H NMR. Spectra were recorded of *c-MYC* Pu24T with complementary flanking sequence in a 1:1 molar ratio at 25 °C. G4 imino signals appear between 10-12 ppm and double-stranded DNA signals appear between 12-14 ppm.

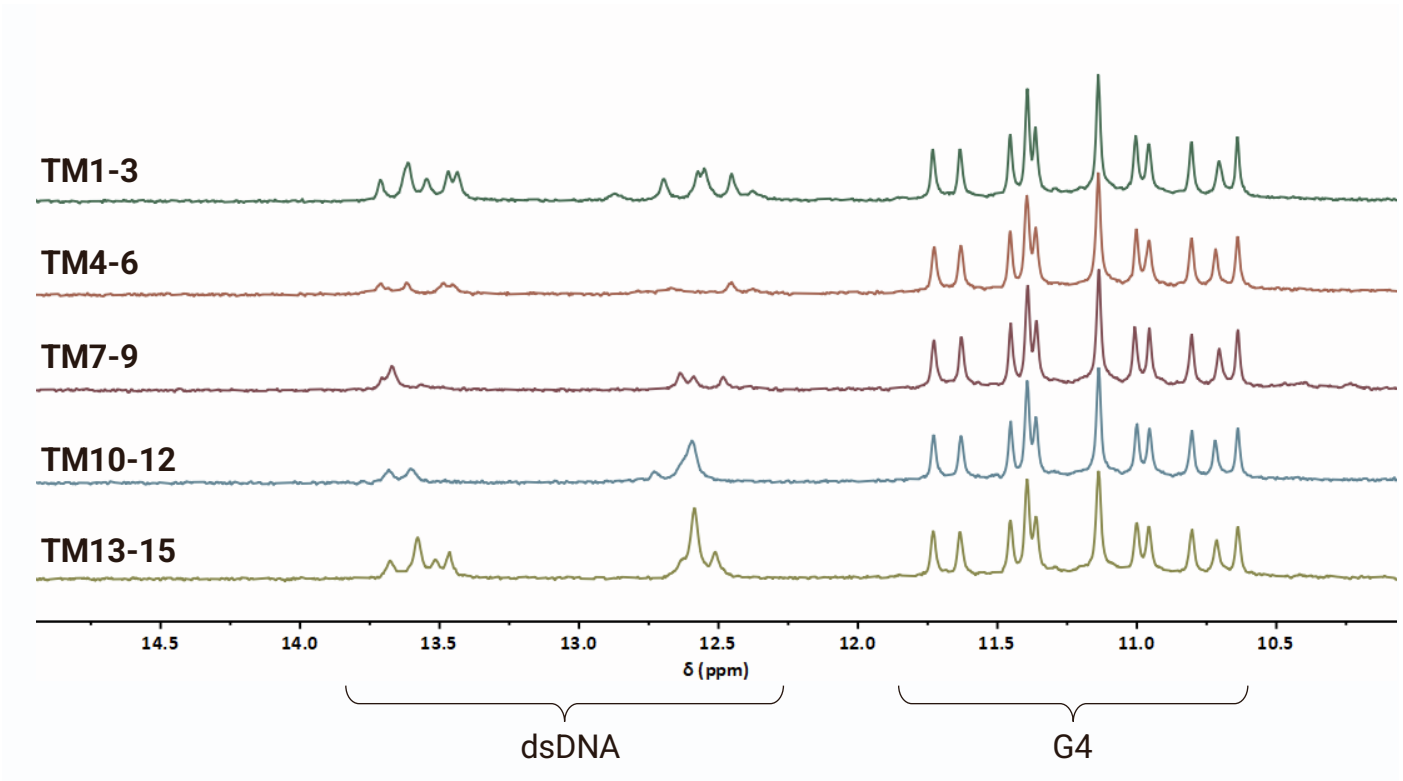


**Figure S30.** Binding interaction of the oligonucleotides **TM 1-3, 4-6, 7-9, 10-12, 13-15** (still contains a 5´hexylamine) measure using $^{1}$H NMR. Spectra were recorded of *c-MYC* Pu24T with complementary flanking sequence in a 1:1 molar ratio at 25 °C. G4 imino signals appear between 10-12 ppm and double-stranded DNA signals appear between 12-14 ppm.

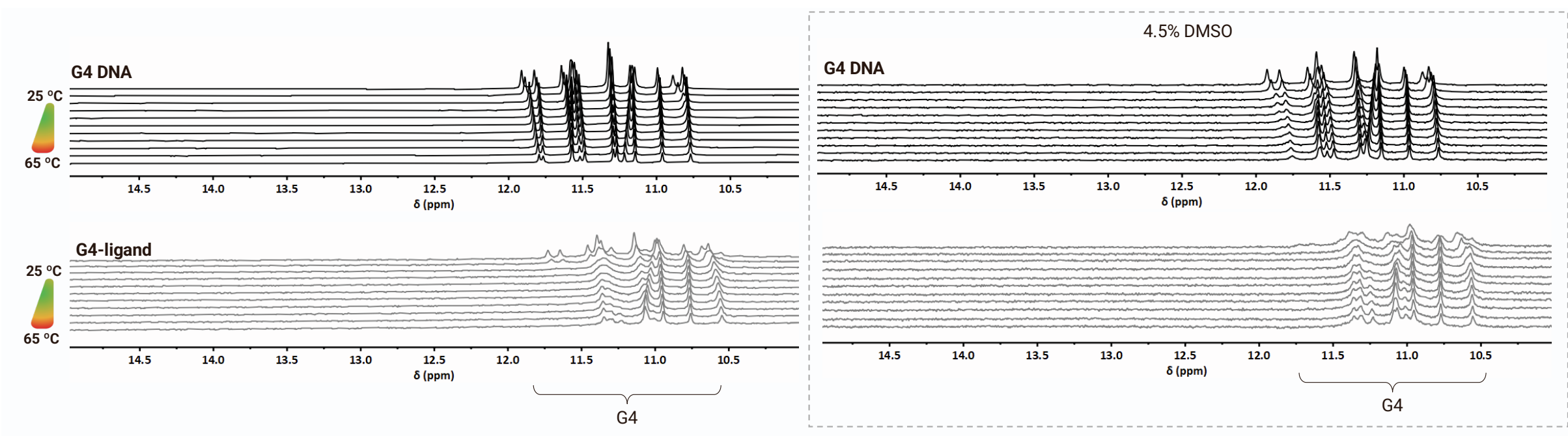


**Figure S31.** Thermal stabilization data of the G4 DNA alone and G4-ligand alone with and without DMSO using $^{1}$H NMR. Spectra were recorded of *c-MYC* Pu24T with complementary flanking at 25 and 35 °C and then with a  temperature ramp of 2.5 °C from 45-65 °C. G4 imino signals appear between 10-12 ppm and double-stranded DNA signals appear between 12-14 ppm.

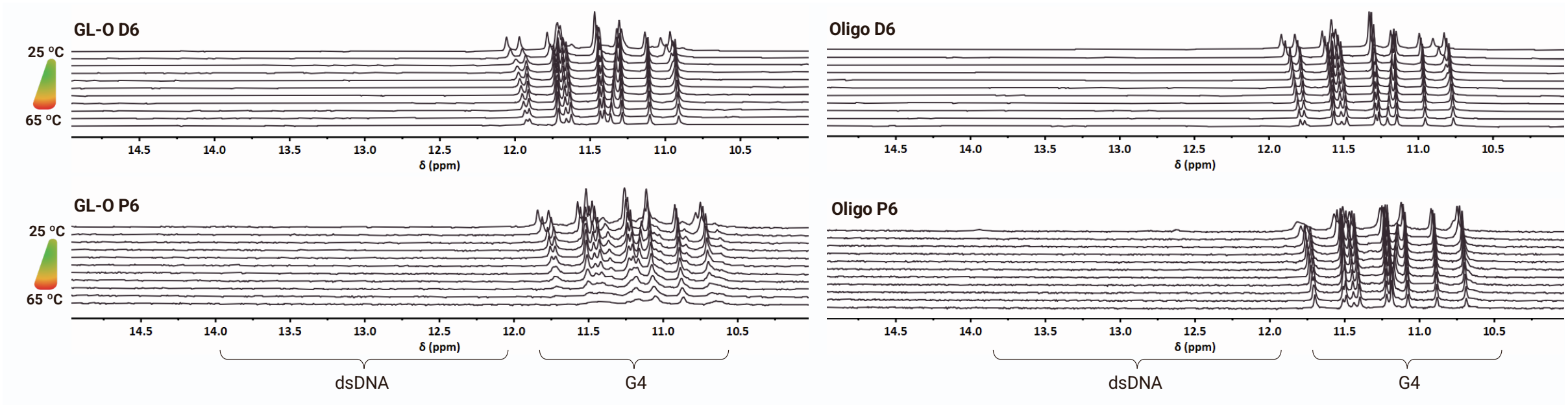


**Figure S32.** Thermal stabilization data of the GL-Os **D6** and **P6** with their unconjugated oligonucleotides using $^{1}$H NMR. Spectra were recorded of *c-MYC* Pu24T with complementary flanking at 25 and 35 °C and then with a temperature ramp of 2.5 °C from 45-65 °C. G4 imino signals appear between 10-12 ppm and double-stranded DNA signals appear between 12-14 ppm.

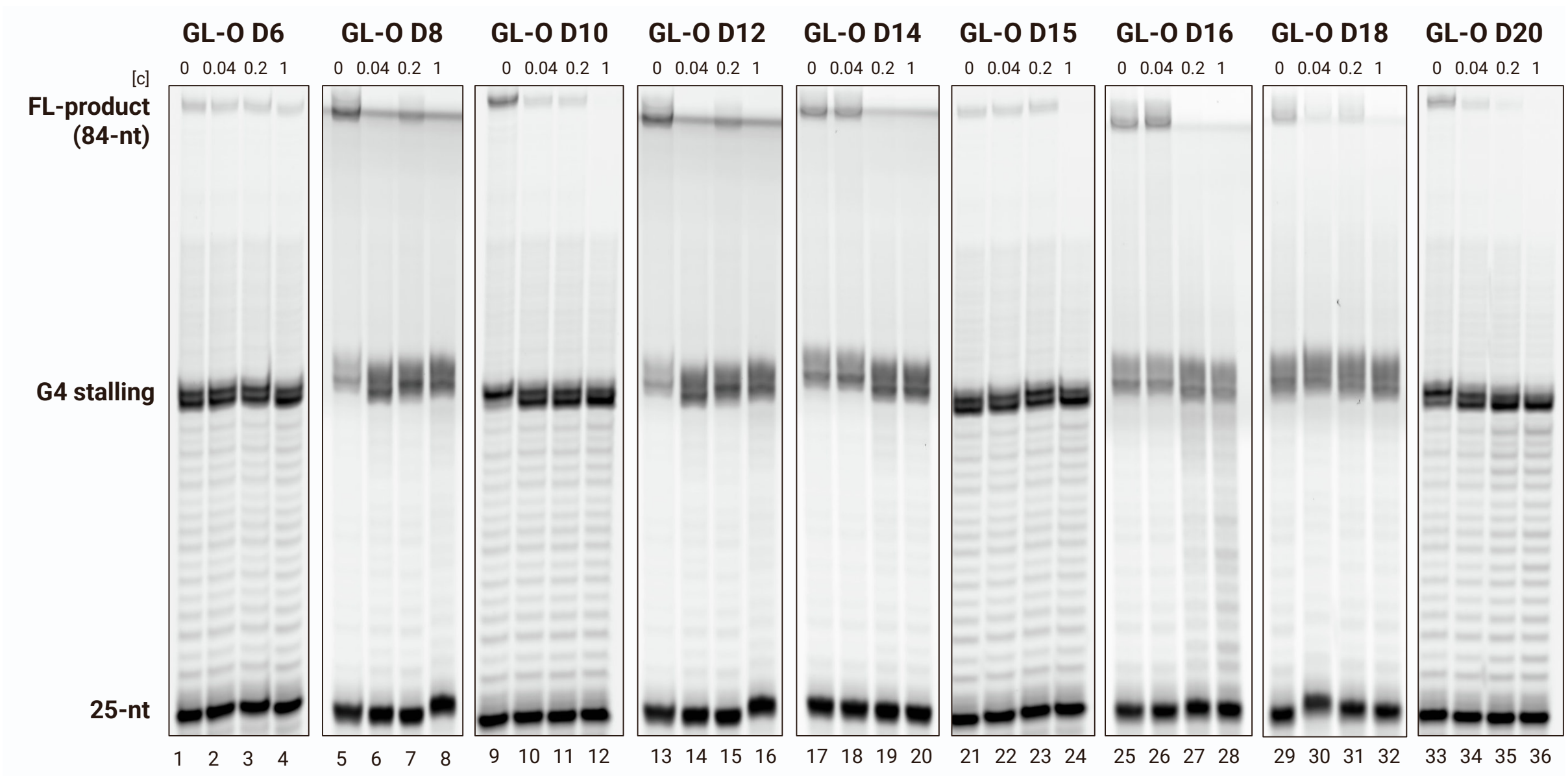


**Figure S33.** Taq Polymerase stabilization of the G4 DNA in presence of varying length oligonucleotide **GL-Os D6-D20**. Quantification of the Taq polymerase stop assay shown in as the full-length (FL, 84-nt) DNA product is expressed as a % of the full-length band intensity that was observed in the control reaction containing only the G4 template. Data represent the mean ± standard deviation from three independent experiments.

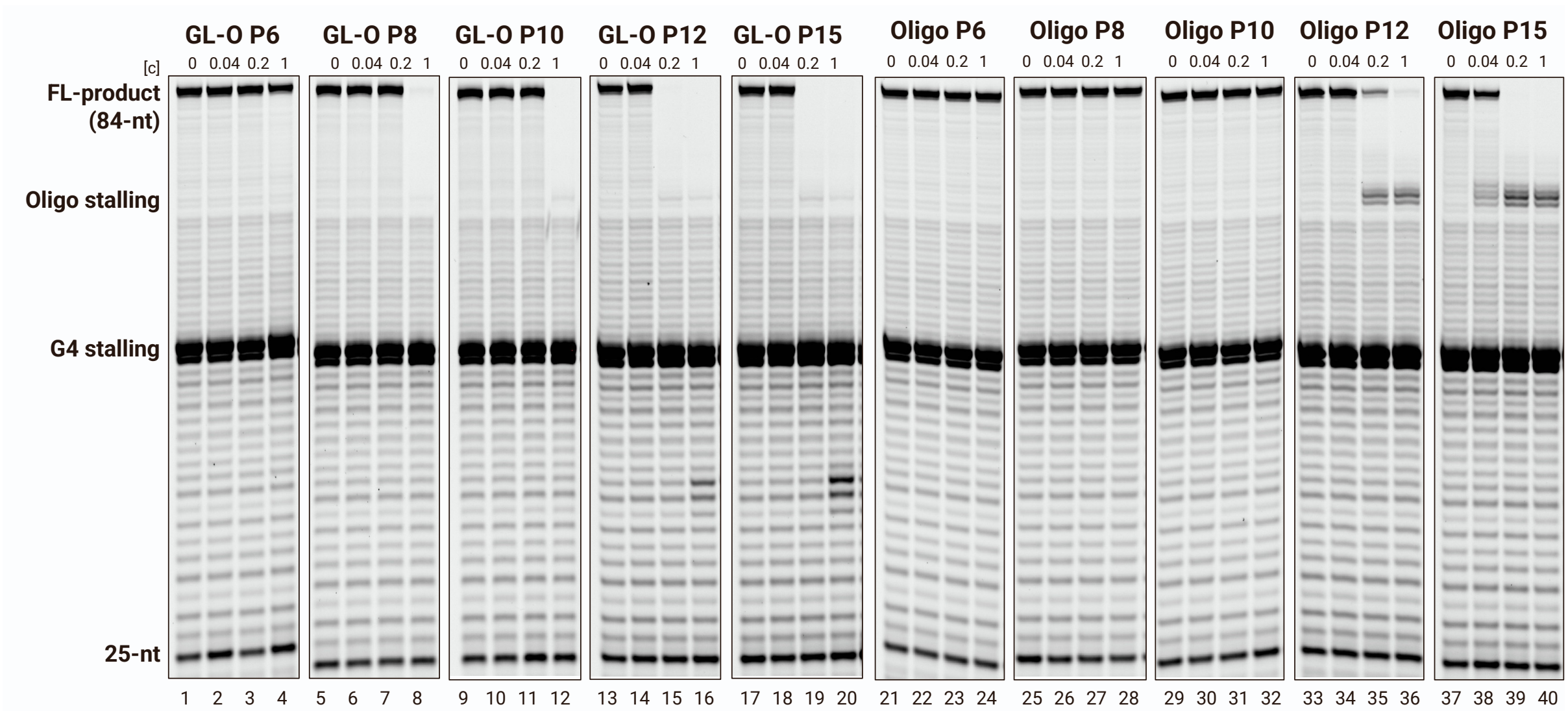


**Figure S34.** Taq Polymerase stabilization of the G4 DNA in presence of varying length oligonucleotide GL-Os **P6**-**P15** (lanes 1-20) and unconjugated oligonucleotides **P6**-**P15** (lanes 21-40). Quantification of the Taq polymerase stop assay shown in as the full-length (FL, 84-nt) DNA product is expressed as a % of the full-length band intensity that was observed in the control reaction containing only the G4 template. Data represent the mean ± standard deviation from three independent experiments.

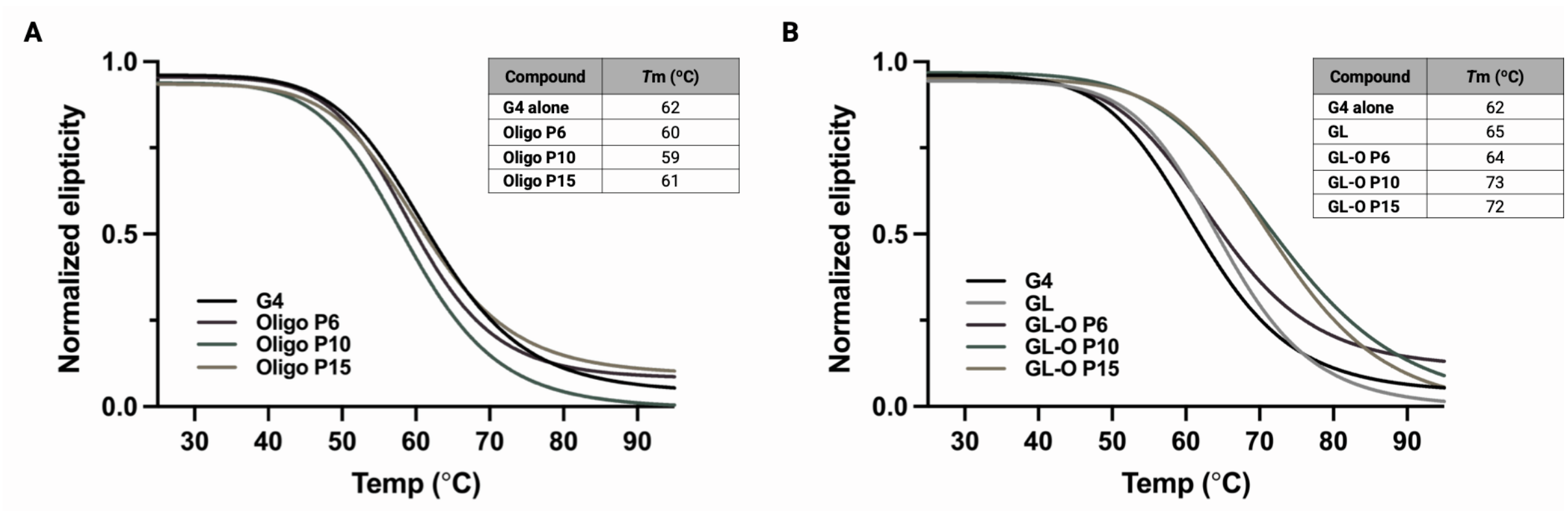


**Figure S35.** CD melting profiles of varying length oligonucleotide GL-Os **P6**-**P15** and unconjugated oligonucleotides **P6**-**P15.** Melting profiles were measured from 25-95 °C.

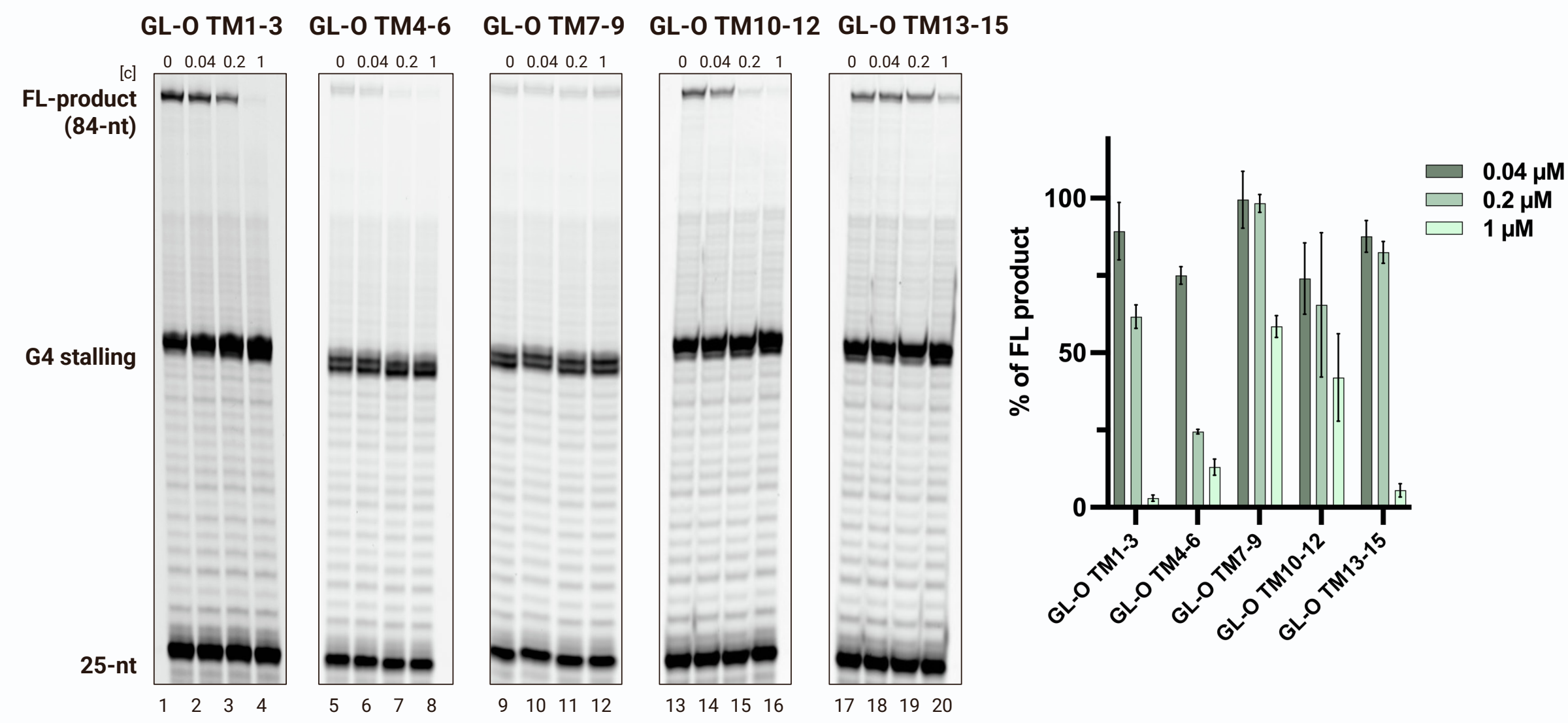


**Figure S36.** Taq Polymerase stabilization of the G4 DNA in presence of varying length oligonucleotide **GL-Os TM1-3**, **TM4-6**, **TM7-9**, **TM10-12** and **TM13-15**. Quantification of the Taq polymerase stop assay shown in as the full-length (FL, 84-nt) DNA product is expressed as a % of the full-length band intensity that was observed in the control reaction containing only the G4 template. Data represent the mean ± standard deviation from three independent experiments.

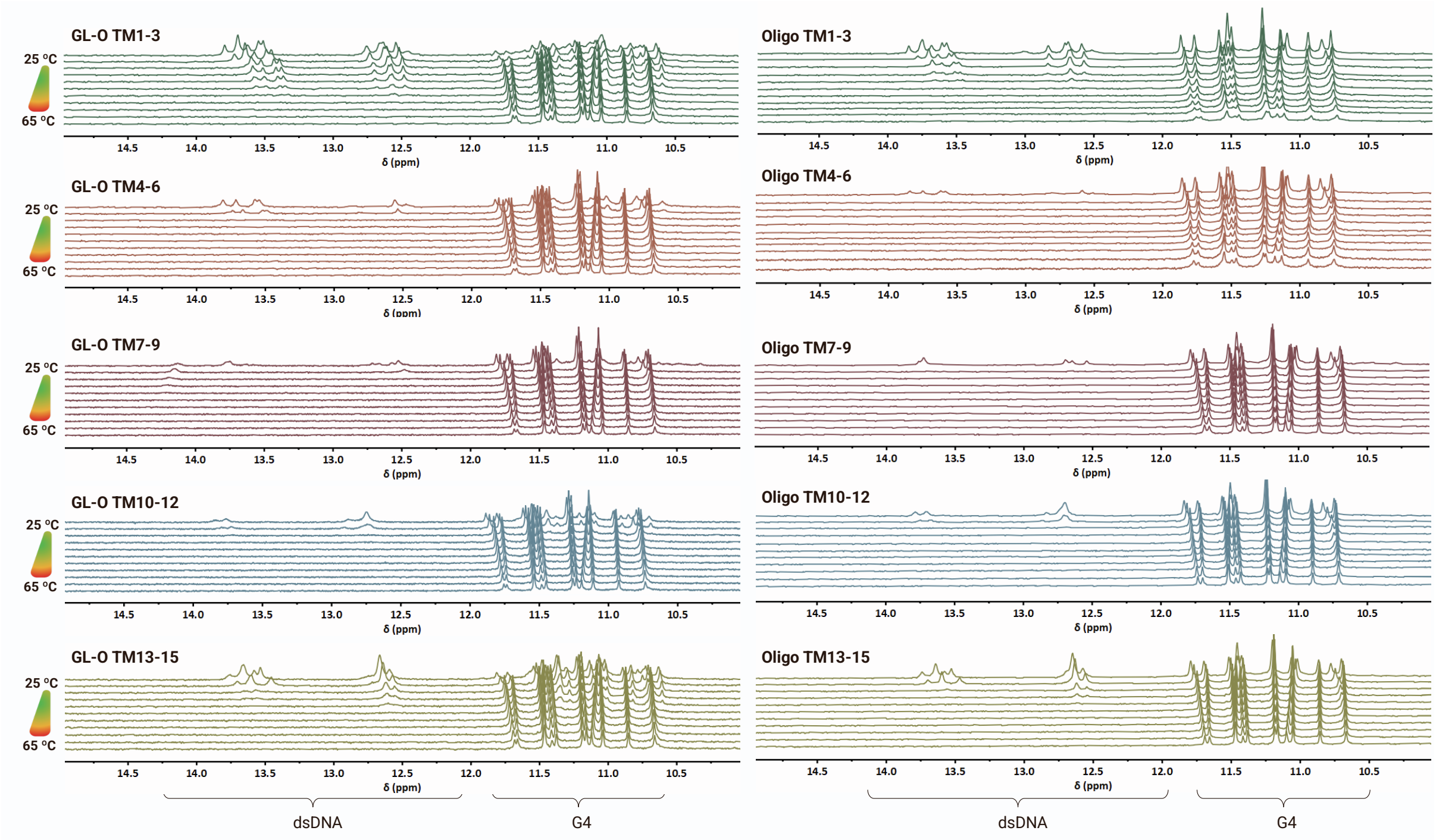


**Figure S37.** Thermal stabilization data of the GL-Os and unconjugated oligonucleotides **TM 1-3, TM4-6, TM7-9, TM10-12, TM13-15** using $^{1}H$ NMR. Spectra were recorded of *c-MYC* Pu24T with complementary flanking at 25 and 35 °C and then with a temperature ramp of 2.5 °C from 45-65 °C. G4 imino signals appear between 10-12 ppm and double-stranded DNA signals appear between 12-14 ppm.

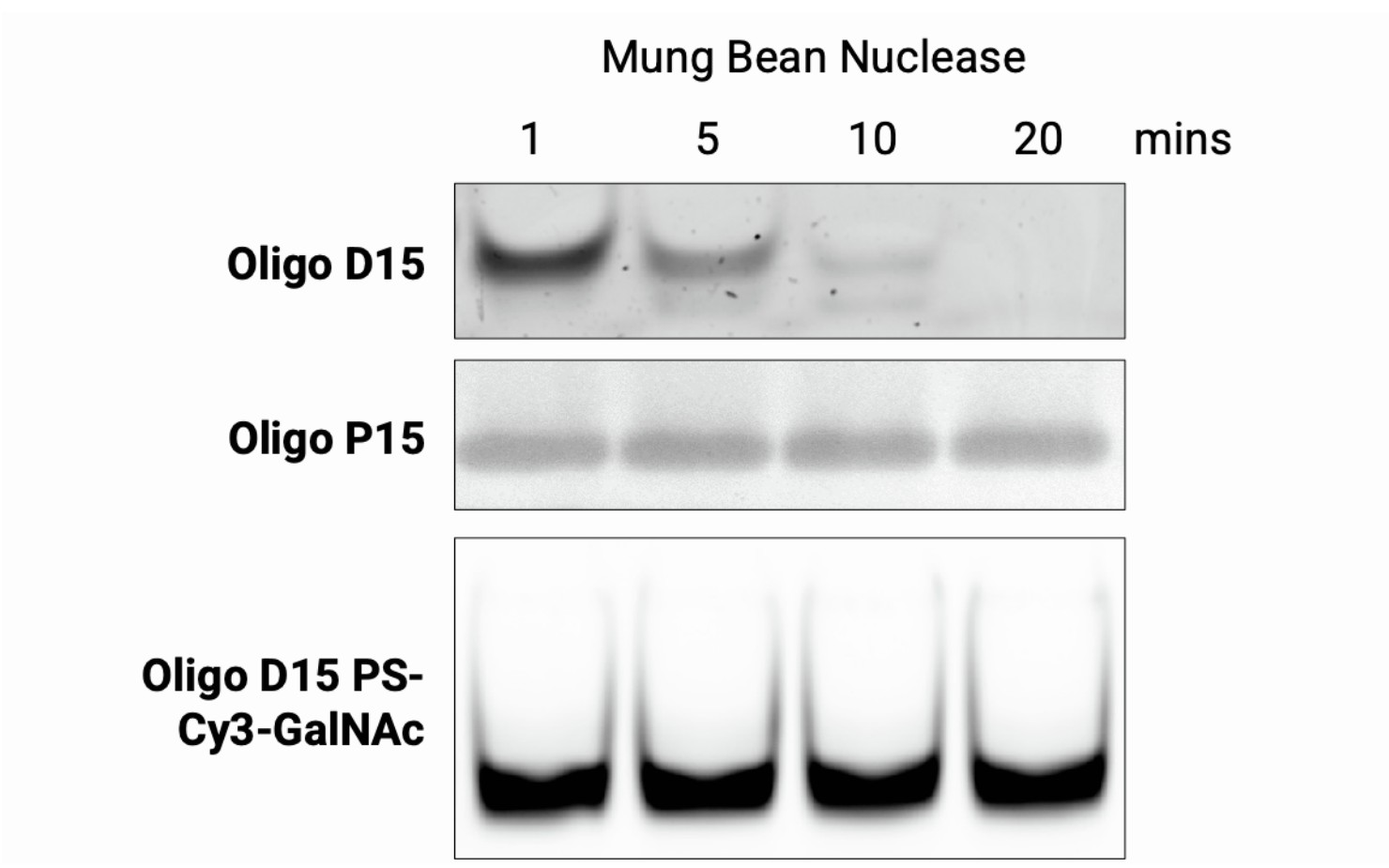


**Figure S38. *In vitro* digestion of unconjugated oligonucleotides by mung bean nuclease.** Unconjugated oligonucleotides were incubated with mung bean nuclease at 30 °C, and aliquots were quenched at the indicated time points. Digestion products were resolved by polyacrylamide gel electrophoresis: DNA oligonucleotides on 16% TBE gels stained with GelRed, and PNA oligonucleotides on 4–20% SDS–PAGE gels stained with InstantBlue. Gels were imaged on a ChemiDoc system (Bio-Rad).

***References***

1. Bhuma, N., Chand, K., Andréasson, M., Mason, J., Das, R.N., Patel, A.K., Öhlund, D., and Chorell, E. (2023). The effect of side chain variations on quinazoline-pyrimidine G-quadruplex DNA ligands. Eur. J. Med. Chem. *248*, 115103.
2. Berner, A., Das, R.N., Bhuma, N., Golebiewska, J., Abrahamsson, A., Andréasson, M., Chaudhari, N., Doimo, M., Bose, P.P., Chand, K., et al. (2024). G4-Ligand-Conjugated Oligonucleotides Mediate Selective Binding and Stabilization of Individual G4 DNA Structures. J. Am. Chem. Soc. *146*, 6926-6935.
3. Casper, J., Speir, M.L., Raney, B.J., Perez, G., Nassar, L.R., Lee, C.M., Hinrichs, A.S., Gonzalez, J.N., Fischer, C., Diekhans, M., et al. (2025). The UCSC Genome Browser database: 2026 update. Nucleic Acids Research *54*, D1331-D1335.
4. Langmead, B., and Salzberg, S.L. (2012). Fast gapped-read alignment with Bowtie 2. Nature Methods *9*, 357-359.
5. Li, H., Handsaker, B., Wysoker, A., Fennell, T., Ruan, J., Homer, N., Marth, G., Abecasis, G., Durbin, R., and Subgroup, G.P.D.P. (2009). The Sequence Alignment/Map format and SAMtools. Bioinformatics *25*, 2078-2079.
